# Analysis of TERT Isoforms across TCGA, GTEx and CCLE Datasets

**DOI:** 10.3390/cancers13081853

**Published:** 2021-04-13

**Authors:** Mathushan Subasri, Parisa Shooshtari, Andrew J. Watson, Dean H. Betts

**Affiliations:** 1 Department of Physiology and Pharmacology, The University of Western Ontario, London, ON N6A 5C1, Canada; msubasri@uwo.ca (M.S.); andrew.watson@schulich.uwo.ca (A.J.W.); 2 Ontario Institute for Cancer Research, Toronto, ON M5G 0A3, Canada; pshoosh@uwo.ca; 3 Department of Pathology and Laboratory Medicine, The University of Western Ontario, London, ON N6A 5C1, Canada; 4 Department of Computer Science, The University of Western Ontario, London, ON N6A 5C1, Canada; 5 The Children’s Health Research Institute—Lawson Health Research Institute, London, ON N6C 2R5, Canada; 6 Department of Obstetrics and Gynaecology, The University of Western Ontario, London, ON N6A 5C1, Canada

**Keywords:** telomere maintenance mechanism, telomerase reverse transcriptase, alternative splicing, Pan-Cancer

## Abstract

**Simple Summary:**

All cancers must maintain telomere length to achieve immortality and around 80% do so by reactivating the enzyme complex telomerase. The diverse regulatory mechanisms surrounding the enzymatic component, telomerase reverse transcriptase (TERT), are often exploited during tumorigenesis to achieve reactivation. Since TERT isoform expression and regulation is heterogenous in nature, we assessed changes in TERT alternative splicing patterns between the normal and neoplastic states across tissue subtypes. We confirmed gene-level TERT overexpression, as well as splicing shifts away from enzymatically non-functional isoforms in neoplastic tissue. Analysis of tissue and cancer-subtype specific TERT expression patterns uncovered heterogenous expression, regulation, and the potential impact of variable telomere maintenance on tumorigenesis. To guide future studies, we clustered cancer cell lines with tumors from related origin based on TERT isoform expression patterns.

**Abstract:**

Reactivation of the multi-subunit ribonucleoprotein telomerase is the primary telomere maintenance mechanism in cancer, but it is rate-limited by the enzymatic component, telomerase reverse transcriptase (TERT). While regulatory in nature, TERT alternative splice variant/isoform regulation and functions are not fully elucidated and are further complicated by their highly diverse expression and nature. Our primary objective was to characterize TERT isoform expression across 7887 neoplastic and 2099 normal tissue samples using The Cancer Genome Atlas (TCGA) and the Genotype-Tissue Expression Project (GTEx), respectively. We confirmed the global overexpression and splicing shift towards full-length TERT in neoplastic tissue. Stratifying by tissue type we found uncharacteristic TERT expression in normal brain tissue subtypes. Stratifying by tumor-specific subtypes, we detailed TERT expression differences potentially regulated by subtype-specific molecular characteristics. Focusing on β-deletion splicing regulation, we found the NOVA1 *trans*-acting factor to mediate alternative splicing in a cancer-dependent manner. Of relevance to future tissue-specific studies, we clustered cancer cell lines with tumors from related origin based on TERT isoform expression patterns. Taken together, our work has reinforced the need for tissue and tumour-specific TERT investigations, provided avenues to do so, and brought to light the current technical limitations of bioinformatic analyses of TERT isoform expression.

## 1. Introduction

Somatic cells have limited replicative capacity, also called the “Hayflick limit”, due to replication-mediated terminal DNA shortening [1]. During DNA replication, the leading strand is synthesized uninterruptedly from the 5′ terminus toward the 3′ unwinding replication fork. In contrast, the lagging strand is synthesized away from the replication fork in small 5′ to 3′ Okazaki fragments to satisfy the unidirectionality of DNA polymerases [2]. As a result, ~50–100 nucleotides are progressively lost during each cellular division from the 5′ end of the lagging daughter strand [3]. To prevent erosion of essential genomic sequences and activation of unwarranted DNA damage responses, human chromosomes are capped with 5–15 kilobases of tandemly repeated hexameric sequences (5′-TTAGGG-3′) [4]. Collectively, these repeats and their associated proteins form the nucleoprotein complex called telomeres [5,6]. While buffering the loss of integral DNA sequences, cellular division will irreversibly shorten telomeres, unless otherwise re-lengthened. The only proven telomere maintenance mechanisms (TMM) are telomerase-mediated lengthening or alternative lengthening of telomeres (ALT) [7]. Sustained replication-induced genomic degradation imposes a selective pressure permitting only the survival of cells acquiring one of these TMMs. Occurring in 1 out of 10^6^–10^8^ cells [8], ~85% of cancer cells (typically of epithelial origin) employ telomerase-mediated telomere maintenance while the remaining ~10–15% (typically of mesenchymal or neuroepithelial origin) utilize ALT [9,10,11]. 

Telomerase, a unique reverse transcriptase (RT) ribonucleoprotein, is made of two core subunits: telomerase reverse transcriptase (TERT) and telomerase RNA component (TERC) [12,13]. The TERC subunit serves as a complementary internal template, while TERT possess catalytic activity and mediate the de novo synthesis of telomeric repeats [14]. Both TERT and TERC are sufficient and necessary for activity in vitro [15]. Additional accessory proteins such as the H/ACA protein complex (NHP2, NOP10, GAR1 and Dyskerin) are associated with telomerase activity (TA) and are required for various regulatory processes in vivo [16,17,18]. Both TA and telomere length (TL) are negatively associated with cellular developmental potency. While germ and stem cells exhibit TA, most terminally differentiated somatic cells have short TL and infrequent TA [12]. Some highly mitotic somatic cell types display appreciable levels of TA. For example, keratinocytes from the basal epidermal layer, late proliferative stage epithelial cells of the endometrium, and leukocytes [19,20,21,22]. Historically, somatic cells were presumed to have zero TERT expression due to poor PCR primer orientation within spliced regions [23]. Now it is clear somatic cells do express TERT, but they are predominantly alternative splice variants (ASV) [23].

**Figure 1:**
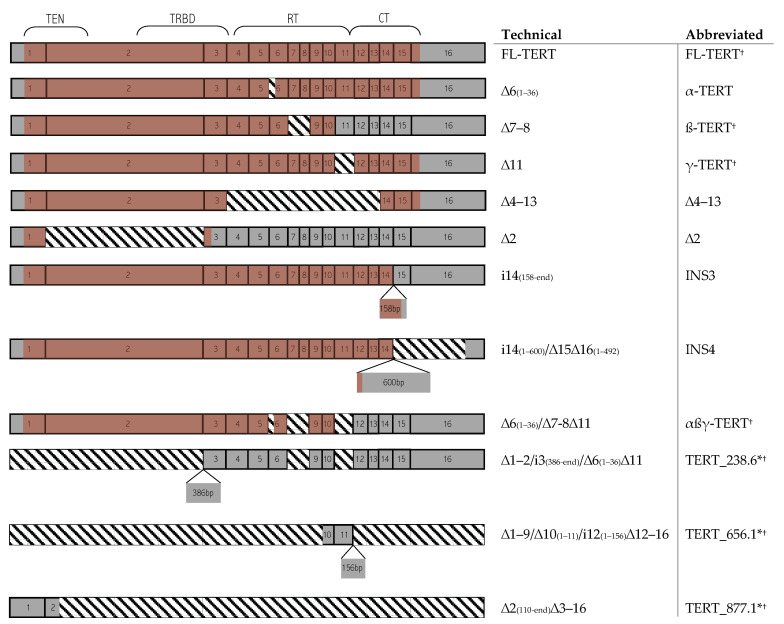
mRNA structure of commonly investigated TERT isoforms and those annotated in Ensembl Build 93. Full-length (FL)TERT consists of 16 exons that make up four domains: TEN, TRBD, RT, and CT. Splicing can involve deletions, which are marked with downward diagonal stripes; or insertions, which are marked with smaller boxes indicating insertional size and an arrowhead indicating the insertional point. ORFs are shown with a light red shade layered over the mRNA structure. Technical isoform name terminology includes “∆” representing deletions and “i” representing insertions. * Abbreviated names are taken from Ensembl transcript ID (TERT_238.6—ENST00000484238.6; TERT_656.1—ENST00000503656.1; TERT_877.1—ENST00000522877.1). ^†^ Indicates transcripts annotated in Ensembl Build 93. All Ensembl transcripts are either automatically generated from the Ensembl genebuild pipeline or manually annotated by human and vertebrate analysis and annotation (HAVANA), supported by transcriptional evidence either from complementary DNA, expressed sequence tags, or protein sequences. Telomerase essential N-terminal domain (TEN); Telomerase RNA binding domain (TRBD); C-terminal extension domain (CT); Central catalytic reverse transcriptase domain (RT); Open-reading frame (ORF).

Full-length (FL) TERT spanning 16 exons is the sole isoform retaining catalytic activity as most splice events perturb at least one of seven RT motifs in exons 4–11 [24]. Currently, 22 human TERT ASVs have been identified in vitro, comprised of various combinations of independent splicing events [23] (Figure 1). Since TERT is weakly expressed, alternative splicing is proposed to attenuate TA, but remains largely unelucidated compared to other transcriptional regulatory methods [25]. Complicating matters is the heterogenous expression patterns observed between and even within tumor types [24,26,27,28]. Tackling this issue, recent studies have utilized a Pan-Cancer bioinformatic approach towards telomere/telomerase research [29,30,31]. However, equivalently assessing matched normal tissue types and tumor-subtypes has not been conducted. By expanding the breadth and depth of inquisition, we may uncover novel conserved or specialized expression patterns that can be clinically exploited. Taken together, our work has elucidated some novel TERT expression patterns in normal and neoplastic tissue subtypes, potential mechanisms for these differences, avenues to explore them in vitro, and the major limitations of RNA-sequencing for the TERT gene and differential isoform expression. 

## 2. Results

### 2.1. TERT Expression across Tumor and Normal Tissues

**Figure 2:**
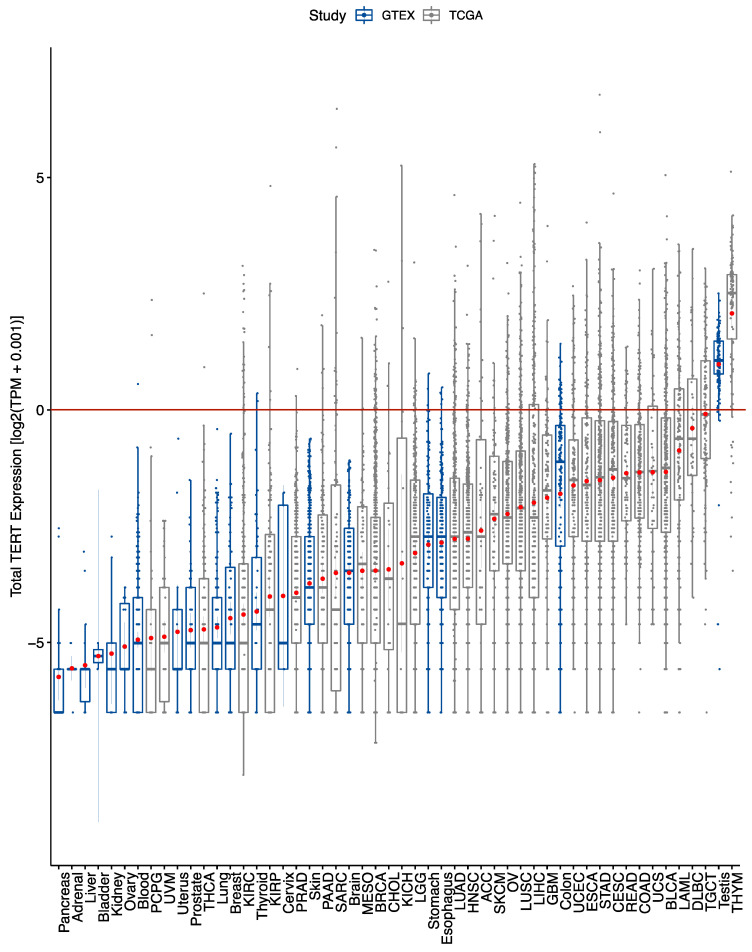
Variable total TERT expression observed across tumour and normal tissue types. Total TERT expression across TCGA tumours shows tumour-specific expression. Total TERT expression across GTEx normal tissue types shows the majority have low expression except for gastrointestinal and testicular tissue types. Box plots boxes denote the inter-quartile range as well as a bolded line representing the median. Extending from the boxes are minimum and maximum lines calculated from 1.5 times the interquartile range. Points outside this range are considered outliers. Within each box is a red point signifying the mean, as well as lines extending from this point representing a 95% confidence interval. Expression values in transcript per million (TPM) were transformed by a log_2_(TPM + 0.001) equation. Horizontal line at y = 0 indicates 1 TPM.

Overall, TERT gene expression was low, with mean expression values less than one transcript per million (TPM) in every cancer type except thymic tumours (Figure 2). Among the 33 cancer types, eight had at least 25% of samples expressing zero TERT (Figure S1). Specifically, these TERT-negative cancer types were related to adrenal, kidney, thyroid, brain or soft tissue origin. In contrast, among the 19 normal tissues types, most (9/16) had at least 50% of samples expressing zero TERT (Figure S2). The seven remaining normal tissue types with majority TERT-positive samples were blood, brain, colon, esophageal, skin, stomach, and testicular tissues. While brain cortical samples were used as the normal reference for gliomas, the highest TERT expression among normal brain tissue subtypes, was in basal ganglia structures (Figure S3). 

### 2.2. TERT Alternative Splice Variants

Most (19/33) tumour types expressed all seven isoforms, 11/33 expressed six isoforms, and 3/33 expressed five isoforms (Figure S4). In contrast, some normal tissue types only expressed 2/7 isoforms (adrenal), 3/7 isoforms (liver) or 4/7 isoforms (ovary, pancreas) (Figure S5). Neoplastic samples primarily expressed FL-TERT, TERT_238.6, and TERT_656.1 (Figure S4). The primary isoform expressed across all normal tissue types was TERT_238.6, followed by FL-TERT in 11/16 tissue types. From the remaining 5/16, adrenal tissue and hepatic tissue did not express FL-TERT at all (Figure S5). 

**Figure 3:**
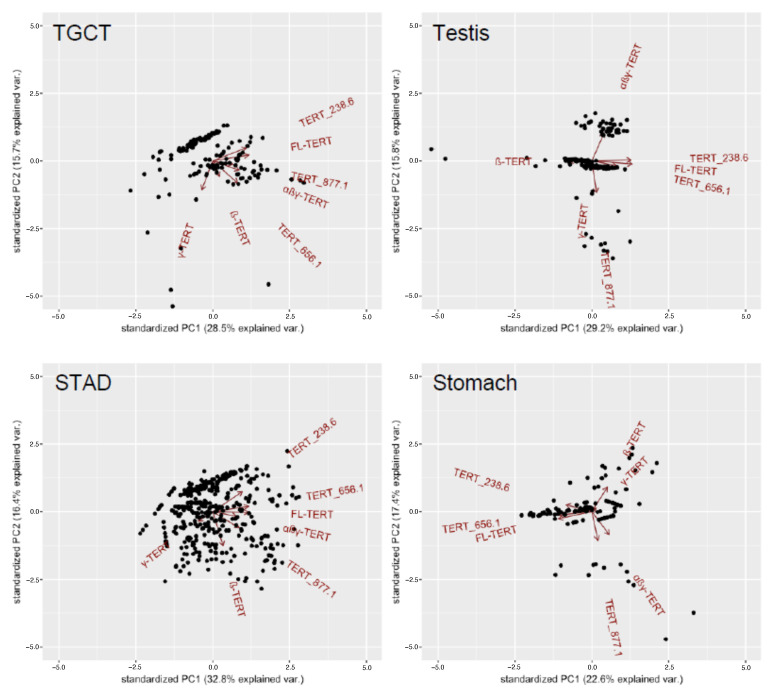
Expression relationships between TERT isoforms differ between tissue types and when in the neoplastic state. PCA biplots for testicular germ cell tumours (TGCT), stomach adenocarcinoma (STAD), and their respective matched normal tissues are shown as examples. Plotted using the first two principal components and show the loading vectors (arrows) of each TERT isoform variable expressed. The arrow length approximates the variance of the variable. The angle between arrows approximates the correlations between variables. Such that parallel arrows in the same direction have positive correlations, perpendicular arrows have no/weak correlations, and parallel arrows in the opposite direction have negative correlations.

PCA biplots across cancer types, with some exceptions, showed that FL-TERT, TERT_238.6, and TERT_656.1 had strong positive correlations with each other (Figure S6). Albeit differences in expression relationships, normal tissue types with greater total TERT expression, such testicular tissue and the gastrointestinal tract tissues, more closely approximated what was observed in tumour tissues. However, there were still clear differences in the relationships between isoform expression between different tissue types and between the neoplastic and non-neoplastic states (Figure 3).

**Figure 4:**
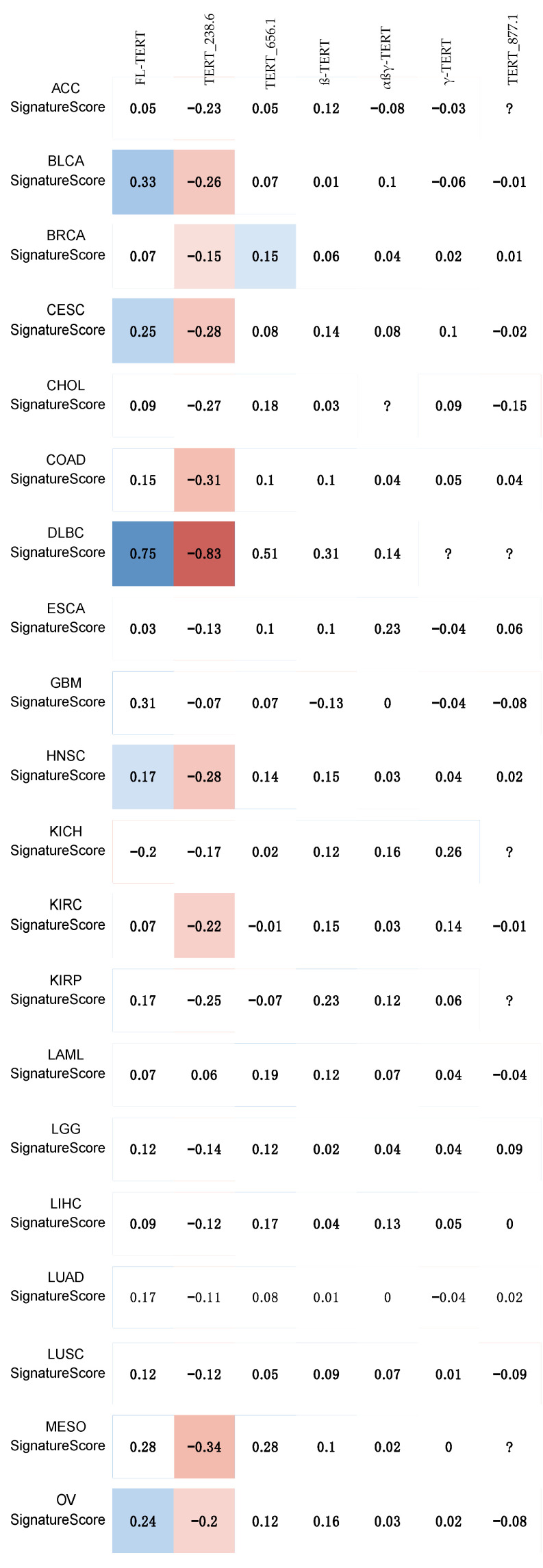

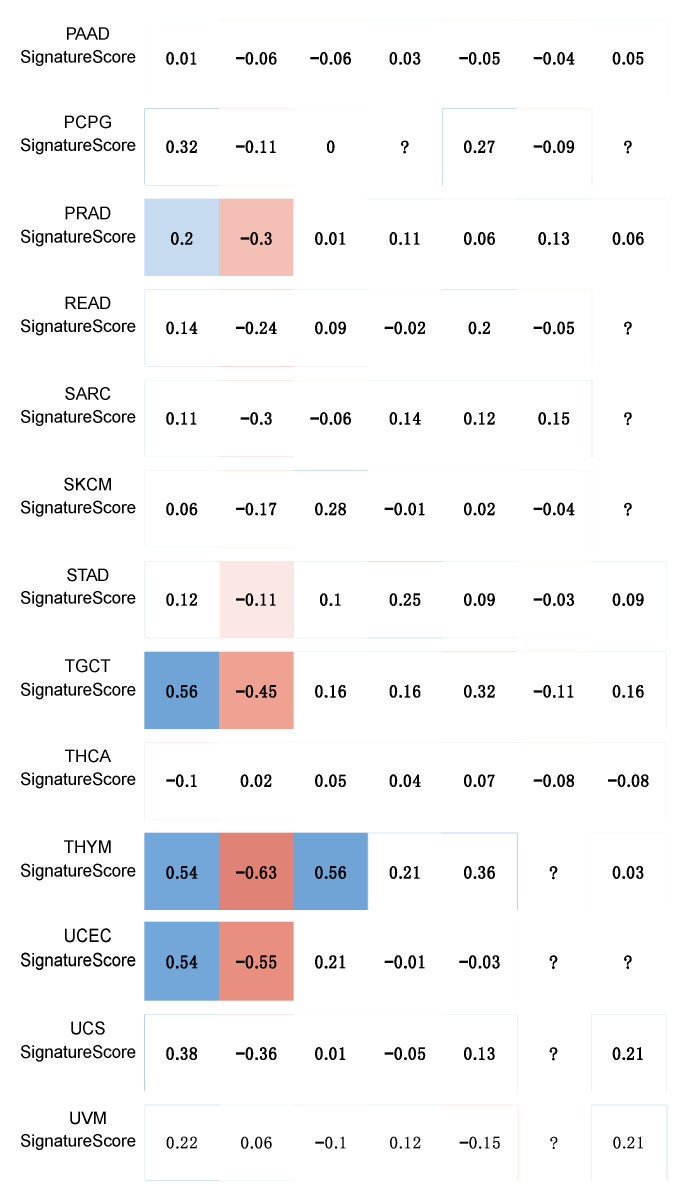
Telomerase activity signature score correlates to increased FL-TERT isoform percentage but decreased TERT_238.6 isoform percentage. FL-TERT isoform percentage (significant in 9/33 cancer types) had all positive correlations. TERT_238.6 isoform percentage had significant negative correlations in 14/33 cancer types and TERT_656.1 had significant positive correlations in 2/33 cancer types. β-TERT, αβγ-TERT, and γ-TERT isoform percentage had no significant correlations to signature score expression. Spearman correlations were computed and significance determined using a Bonferroni corrected *p*-value of <0.000216 (0.05/231 comparisons). Correlations were highlighted with colour only if significant, blue indicating a significant positive correlation, and red indicating a significant negative correlation. Spearman’s coefficients are displayed, and colour intensity also indicates the strength of correlation. Question marks (“?”) indicate no correlation could be computed between signature score and the respective TERT isoform.

**Figure 5:**
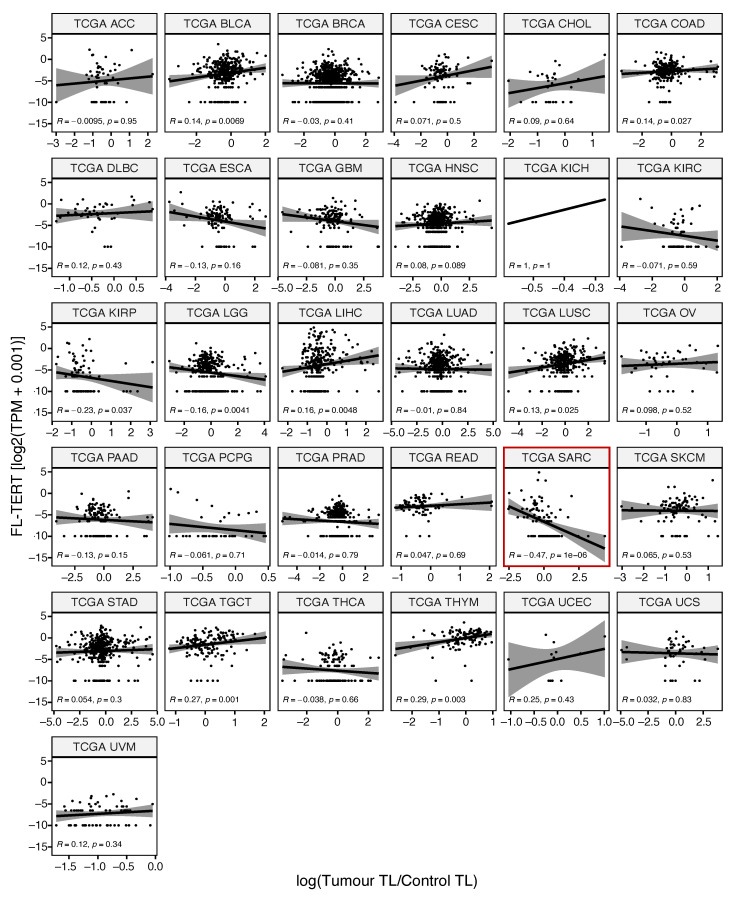
FL-TERT isoform expression has a significant negative correlation only with telomere length ratio in sarcomas. Only SARC had significant correlation, which was negative. Telomere length ratios and FL-TERT expression were log_10_ and log_2_(TPM + 0.001) transformed, respectively. Scatter plots were fitted with a linear regression line and 95% confidence interval. Spearman correlations were computed, and significance determined using a Bonferroni corrected *p*-value of <0.000230 (0.05/217 comparisons). Significant correlations are highlighted with a red border.

Comparing telomerase activity gene signature expression to TERT_238.6 isoform percentage found significant negative correlations in 14/33 (~42%) cancer types (Figure 4), aligned with previous findings of TA inhibition by β-deleted TERT isoforms [32,33]. Finally, comparing TERT isoform expression with relative telomere length, a surprising significant negative correlation between FL-TERT expression in sarcomas was found (Figure 5). Interestingly, TGCT and THYM had significant positive correlations between telomere length and TERT_238.6 expression, but not with FL-TERT expression (Figure S7). Seeing as these cancer types had the highest total TERT expression and TERT_238.6 was the predominant isoform in most samples, this relationship may simply be reflecting one of total TERT expression and telomere re-lengthening. No other correlations between TERT isoform expression and relative telomere length were observed (Figures S7–S12). 

### 2.3. Tumor-Specific Subtype-Dependent Differences in TERT Isoforms

To fully define TERT isoform expression heterogeneity, we evaluated the tumor-specific subtype-dependent differences. Several cancer types (14/29) presented at least one significant (*p* < 0.05) difference related to TERT expression or TL ratio. Cancer-specific molecular characterization papers of liver hepatocellular carcinoma (LIHC), lower grade glioma (LGG), and glioblastoma multiforme (GBM) have described mechanisms for their subtype-dependent differences in TERT expression [34,35]. For the remaining 11 cancer types, we postulated potential subtype-specific mechanisms by which TERT expression and TL are altered, and their resultant functions (Table 1). These mechanisms ranged from alterations of known TERT transcriptional influencers (i.e., NOTCH1, KRAS, FGFR3, ER), to heightened cellular potency, direct TERT alterations or larger genomic instabilities, TMM decision fate (i.e., telomerase-mediated or ALT), and confounding factors (i.e., immune cell infiltration). 

### 2.4. Cancer Cell Line TERT Isoform Expression Patterns

**Figure 6:**
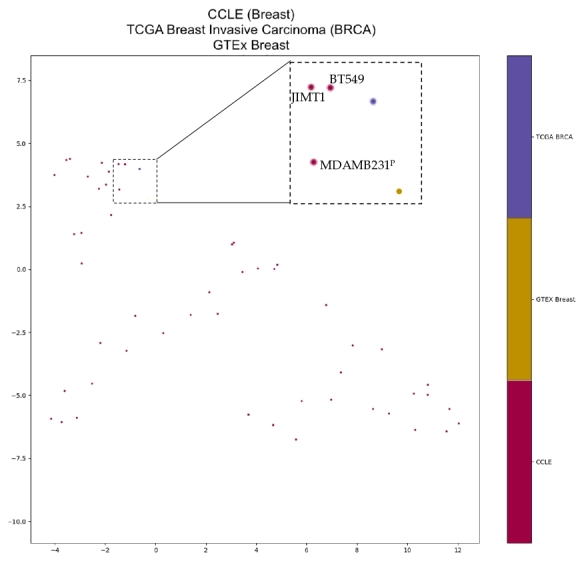
UMAP projection of breast CCLE cell lines, TCGA breast invasive carcinoma (BRCA), and GTEx breast using TERT isoform expression. Cell lines JIMT1 and BT549 were closest to average isoform percentage from TCGA BRCA. Cell line MDAMB231 was closest to average isoform percentage from GTEx breast. Cell line TERT promoter status taken from Ghandi et al., 2019. Superscript “P” indicates TERT promoter mutation, superscript “WT” indicates wild-type TERT promoter, and no superscript indicates no data available. Dashed-line box indicates a zoomed in region of interest with text labels of cell lines. UMAP Parameters: Manhattan distance, 8 Neighbours and 4 Components.

In addition to heterogeneity in TERT expression patterns across and within tumor types, cancer cell lines are also reported to exhibit TERT isoform expression heterogeneity [27,28]. Suitable cell line selection is essential to accurately reflect the TERT transcriptome observed in primary tumors. We used Uniform Manifold Approximation and Projection (UMAP) to project average TERT isoform expression patterns in primary tumors with Cancer Cell Line Encyclopedia (CCLE) for 19 cell origin types (Figures S13–S30). The UMAP projection for breast carcinoma and related cancer cell lines is shown (Figure 6). Several cancer cell lines aligned closely with primary tumor TERT transcriptomes, while others did not (Table 2). While not all cell lines were profiled for TERT promoter mutational status [65], those with mutational status were annotated. Aligned with the TERT promoter mutation frequency that is observed in primary tumors [66], most lung cancer cell lines were wild-type (WT) while skin cancer lines frequently harbored promoter mutations. 

## 3. Discussion

### 3.1. β-Deletion General Prevalence but Cancer-Specific Regulation

The necessity of telomere maintenance for neoplastic transformation has resulted in multiple studies characterizing TERT overexpression during tumorigenesis. Using the TCGA dataset, Barthel et al. observed TERT alterations in 95% of TERT expressing samples and regardless of alteration type, all TERT altered groups displayed higher TERT expression than wild-type [29]. Similarly, we observed holistic TERT overexpression in tumor samples. At an isoform level, while tumor samples had higher TERT_238.6 expression, they typically had lower TERT_238.6 isoform percentage, indicating a shift in splicing away from FL-TERT. We also appreciated the strong negative correlation between TERT_238.6 isoform percentage and TA signature score expression—a finding that coincides with the negative inhibition that β-TERT confers on TA [32]. Seeing as the β-deletion, harbored by TERT_238.6, is most robustly expressed, recent efforts have uncovered regulatory features governing β-splicing. Using a TERT mini-gene construct containing exons 5–10, three short intronic repeats essential for β-splicing were identified: intron 6 (B6 and DR6) and intron 8 (DR8) repeats [68]. Mechanistically, B6 undergoes RNA:RNA pairing with sequences in intron 8 to promote the β-deletion (removal of exons 7 and 8) by reducing the physical space between the splice sites at exon 6 and 9 [69]. Using varying combinations of these three cis-regulatory elements in mini-gene constructs and antisense oligonucleotides (ASO) to sterically hinder splice sites, B6 was considered necessary and sufficient for substantial β-deletion; DR6 potentiated β-deletion and DR8 were necessary for FL-TERT production [68]. However, a follow-up study using two lung cancer cell lines showed that DR8-mediated splicing control is cell-type specific, highlighting the complexity regarding TERT expression regulation [70]. With respect to *trans*-acting factors, the only experimentally supported factors to directly bind TERT pre-mRNA and direct splicing are NOVA Alternative Splicing Regulator 1 (NOVA1), Polypyrimidine-Tract Binding Protein 1 (PTBP1), and most recently RNA Binding Motif Protein 10 (RBM10) [70,71,72]. Firstly, using lung cancer cells, NOVA1 was shown to bind to a conserved motif in DR8 to promote FL-TERT splicing, TA, and telomere maintenance [70]. Moreover, PTPB1 was recruited by NOVA1 to DR8 motifs and also promote FL-TERT splicing, TA, and telomere maintenance [71]. Secondly, in pancreatic cancer, Xiao et al., used findings from the mini-gene constructs and mutation data from the TCGA dataset to identify and show that reduced RBM10 expression was significantly related to poorer survival [72]. Then, it was experimentally reported that RBM10 binds “GGU” motifs within the 5′ splice site of introns 7–8 of TERT pre-mRNA and promotes the exclusion of exons 7 and 8 to generate the β-deletion in pancreatic cell lines [72]. 

As such, we aimed to translate the NOVA1/PTBP1 findings to the TCGA RNA-sequencing dataset. We grouped samples into FL-TERT expressing (isoform percentage > 0) and non-expressing (isoform percentage = 0) for all 33 cancer types. Comparing the expression of NOVA1 between these two groups, we only observed significantly higher NOVA1 expression in the FL-TERT-expressing group for only lung adenocarcinoma (LUAD), but significantly lower expression in the STAD and breast invasive carcinoma (BRCA) FL-TERT-expressing groups (Figure S31). In contrast, PTBP1 was significantly higher in the FL-TERT-expressing group for 10/33 cancer types (Figure S32). It is possible that PTBP1 is a ubiquitous regulator of FL-TERT splicing, working in concert with another tissue-specific *trans*-acting factor to direct DR8 bindings. This role is fulfilled in lung cancers by NOVA1, but the opposite seems to occur for other cancer types regarding NOVA1. This underscores the tissue and cell-type specific regulation of TERT expression and splicing.

### 3.2. TMM Decision Fate

Our tumor-subtype evaluations highlighted the various potential modalities of TERT expression changes. Notably for SARC, the smooth muscle differentiated leiomyosarcomas (LMS) subtype was characterized by low TERT expression, high RB1 mutation frequency, and no association between α-thalassaemia/mental retardation syndrome X-linked (ATRX) mutations and telomere lengthening. ATRX and/or death domain-associated protein (DAXX) inactivation is commonly observed in ALT-positive tumors [73,74]. Together, they aid in the deposition of histone variant H3.3 into telomeres and while the resulting functions are unclear, ATRX/DAXX mutations are linked to telomere dysfunction [75]. Interestingly, a subset of tumors do not express TERT nor harbor ATRX/DAXX, but are enriched for retinoblastoma protein (RB1) mutations [29,57]. Therefore, the ALT phenotype may be activated through an independent pathway involving inactivation of RB1, but not ATRX/DAXX [57]. Whether decreased TERT expression is a consequence or cause for ALT fate determination is unclear. Furthermore, we investigated the relationship between TL and TERT isoforms and found SARC to have a significantly negative correlation between TL ratio and FL-TERT expression; a result previously observed in mesenchymal sarcomas [76]. Cancer cells are widely accepted to have shorter telomeres than matched normal tissue [29,77], which are attributed to telomerase reactivation selection pressure once telomeres are critically short. Upon reactivation, subsequent re-lengthening may function in a maintenance capacity. However, ALT-predominated tumors like SARC have elongated telomeres, attributed to the ALT process involving homologous recombination [78]. Therefore, sarcomas likely undergo a determinative process for employing ALT-mediated telomere maintenance, resulting in longer telomeres or telomerase-mediated telomere maintenance resulting in shorter telomeres. Changes in TERT-related pathways, such as gene amplifications in dedifferentiated liposarcomas (DDLPS) or FGFR3 activation in synovial sarcomas (SS), likely promote telomerase-mediated telomere maintenance. Whereas, those in ALT-related pathways, such as RB1 in LMS sarcomas, likely promote ALT-mediated telomere maintenance. Interestingly, the classical hypothesis that TMM decision fate is absolute has been challenged with evidence of both mechanisms coexisting in cancer cells. This suggests that TMM fate can be dynamic and parallels the dynamics of epithelial-mesenchymal transitions [77,79].

### 3.3. Heterogenous and Specific TERT Transcriptomes

Perturbed TERT gene expression, whether over-expressed, under-expressed or dysregulated, is associated with many disease processes. As such, TERT is regulated by various molecular modalities: pre-transcriptionally, post-transcriptionally, and post-translationally [25]. Our examination of TERT isoform expression patterns across tissue-types highlighted consensus FL-TERT and TERT_238.6 expression and splicing shifts during tumorigenesis. In addition, tissue-specific TERT transcriptomes were observed. Among normal brain tissue subtypes, basal ganglia structures had the highest TERT expression. TERT expression is important for neurodifferentiation and survival using rodent models [80]. While TA declines with brain development in humans [81], the expression and neuroprotection persists in adult rodent brain structures [82,83,84]. However, there are important species-dependent differences between human and rodent TERT. Notably, the rodent TERT promoter is significantly more active and rodent somatic tissues have elevated TA [85]. Nonetheless, TERT dysfunction may contribute to age-associated telomere shortening and neurodegenerative diseases involving the basal ganglia such as Parkinson’s and Huntington’s diseases [82,86]. While this paper focused on FL-TERT overexpression in the context of cancer, under-expression of FL-TERT and consequent telomere shortening is associated with telomeropathies including idiopathic pulmonary fibrosis, dyskeratosis congenita, and aplastic anemia [87]. While one potential molecular change that would result in reduced FL-TERT expression is splice site mutations, there are no direct relationships or functional consequences reported between increased TERT ASV expression and telomeropathy prevalence or disease severity. 

In contrast, splicing dysregulation as a mechanism of FL-TERT overexpression has been associated with cancer promotion and progression [24]. Our work further reinforces the cell-type specific nature of TERT splicing dysregulation presented in the literature. For example, the β-deletion has been shown or speculated to be regulated by RMB10 in pancreatic cancer [72]; NOVA1/PTBP1 in lung cancer [70,71]; SRSF11, hnRNPH2, and hnRNPL in breast cancer [32]; and MCPH1/BRIT1 in ovarian cancer [88]. The malignant consequences of FL-TERT overexpression are two-fold. In the first instance, cellular immortalization is crucial for cancer cell survival and unattainable without sustained telomere maintenance [8,9,10,11]. In the second instance, TERT has many proposed non-canonical extra-telomeric roles, primarily by regulating gene expression as a transcriptional factor/co-factor [89]. Of note, TERT is involved in Wnt/β-catenin [90,91] and Nuclear Factor kappa-light-chain-enhancer of activated B cells (NF-κB) signaling [92]. These pathways control phenotypes exacerbated in cancer, namely cell survival, proliferation and migration. Recent evidence has also shown that ALT tumours may have decreased metastatic potential due to the lack of non-canonical functions [93]. This notion is reinforced by studies demonstrating the positive relationships between TERT and vascular endothelial growth factor (VEGF) [94], epithelial-to-mesenchymal transition [95], up-regulation of growth-promoting genes and down-regulation of pro-apoptotic genes [96], and mitochondrial resistance to stress [97,98]. While the nuances of these pathways have yet to be teased out, it is clear a flourishing environment is conferred through TERT overexpression. 

Not as clear are the non-canonical extra-telomeric roles of TERT ASVs. To date, it has been shown that overexpression of β-TERT in breast cancer cells results in an anti-apoptotic chemoprotective phenotype [32], and overexpression of the ∆4–13 TERT variant in sarcoma cells induced proliferation via Wnt-signaling activation [23]. Therefore, it is plausible that some TERT ASVs retain the essential domains for similar non-canonical extra-telomeric consequences as FL-TERT but do so less efficiently—only presenting when artificially overexpressing beyond physiological abundances. Appreciating the existing limitations on functional research without TERT ASV-specific antibodies, it is still imperative to elucidate these possibly cancer-specific extra-telomeric functions so that therapeutic strategies to manipulate splicing away from FL-TERT can be developed, without realizing unintended adverse effects. 

To aid future investigations and navigate the heterogeneity of TERT transcriptomes, we attempted to find suitable cancer cell lines for primary tumor types. While our clustering was based solely on TERT expression, other groups have done similar associations using the whole “omic” data [67,99]. Particularly, Yu et al. utilized the whole transcriptome to identify a comprehensive panel (TCGA-110-CL) of cell lines for 22 tumor types [67]. Interestingly, 9/21 cancer types (excluding SKCM because Yu et al. used metastatic samples) had at least one cell line recommended by both TCGA-110-CL and our TERT-based clustering [67]. This reinforces the use of the outlined cancer cell lines in these nine tumor types for TERT isoform research. 

### 3.4. Limitations

While similar splice events to those in TERT_238.6, TERT_656.1, and TERT_877.1 have been observed [23], their complete transcripts have not been observed. TERT_238.6 has been cited only twice, and Slusher et al. referred to it simply as β-deleted TERT, despite also containing other splice events [31,100]. Using *NCBI ORFFinder* [101], the longest ORFs for TERT_238.6 spanned was ~250 amino acids, in comparison to FL-TERT made of 1132 amino acids. Thus, TERT_238.6, if transcribed, likely undergoes non-sense mediated decay (NMD), unlike β-TERT, which undergoes NMD [102] but is also translated [32]. TERT_656.1 and TERT_877.1 are both composed of approximately two exons. Recently, Sayed et al. used targeted long-read length RNA sequencing and found ∆5–15 (deletions of exons 5 through 15) and ∆4–15 (deletions of exons 4 through 15) were among the most abundant TERT transcripts. While short TERT transcripts have been observed, there are potential avenues for sequencing-based misinterpretation.

Particularly, poly(A) RNA selection results in a 3′ bias of selected RNA transcripts [103], the high 5′ guanine-cytosine content in exons 1 and 2 of TERT hinders accurate detection [104], and the scarcity of TERT expression requires large sequencing depth [105]. For these reasons, Barthel et al. opted to focus on sequencing and analysis of only exons 6–9, limiting coverage solely to the β-deletion. Moving forward, these reservations must be kept in mind until sequencing biases are mitigated, and an accurate transcript annotation is used to reflect TERT isoforms observed in vitro. Using a combination of targeted RNA enrichment and direct long-read RNA-sequencing from the 3′-poly(A) tail to 5′cap will allow for the most accurate identification and quantification of TERT isoforms [31,103]. 

Finally, the transcriptome annotations used for TCGA/GTEx and CCLE datasets were different, resulting in seven and eight annotated transcripts, respectively. However, the additional transcript used for CCLE was similar in RNA structure and identical in protein structure to β-TERT (Figure S11). Thus, reads were pooled with β-TERT for the CCLE dataset to match the TERT transcript annotation used for primary tumors. That is not to say the distribution of RNA sequencing reads would be congruent if the CCLE dataset was recomputed using a more recent TERT transcript annotation. 

## 4. Materials and Methods 

### 4.1. Datasets 

We obtained TCGA [106,107] and GTEx [108,109] RNA sequencing expression data from the UCSC Toil RNA-seq recompute data hub ([110], accessed on 1 May 2020) at the UCSC Xena server [111,112]. Toil pipeline used the GRCh38 reference genome and Gencode v23 (Ensembl Build 93) transcript annotation. TCGA samples were filtered for only primary solid and primary blood derived tumors (Figure S33). While the TCGA project also excised and sequenced adjacent normal tissue samples from some cancer patients [113], these “normal” tissue samples may be affected by neighboring tumor cells [114,115,116,117], which is why GTEx samples were used for matched normal tissues. Samples having zero transcripts per million (TPM) for total TERT expression were removed (Figure S33). The total number of annotated transcripts for the Toil Recomputed dataset was 198,620; resulting in an average transcript relative abundance of ~5 TPM. Tumor subtype data was taken from landmark cancer-specific papers (Table S1). CCLE transcript expression and phenotypic characteristics were downloaded from the CCLE database ([118], accessed on 1 May 2020). CCLE [65] RNA-seq data was computed using the GRCh37 reference genome and Gencode v19 (Ensembl Build 75) transcript annotation. In this dated annotation, there is an additional TERT transcript (ENST00000296820.5; we abbreviated as TERT_820.5). RNA sequencing reads attributed to TERT_820.5 were pooled with β-TERT (TERT_104.2) due to their similar mRNA structure and identical protein structure (Figure S34). 

### 4.2. Telomerase Activity Gene Signature Correlation Analysis 

Telomerase activity-related signature scores were calculated by summing a 43 gene set’s TPM expression values (Table S2). Briefly, Barthel et al. performed differential gene expression analysis on Gene Expression Ominibus microarray data from four telomerase positive and four telomerase negative de-differentiated liposarcoma samples. This analysis resulted in 1302 genes that were enriched (fold-change ≥ 1.5) in telomerase positive samples. After refinement, this list was reduced to 43 genes and validated, but did not reach significance in 11 urothelial cell carcinoma cell lines (Rho = 0.58, *p* = 0.07) [29]. Spearman’s correlation was used to identify any relationships with TERT isoform percentages. Using 7 isoforms and 33 cancer types, significance was determined with a Bonferroni corrected *p*-value of <0.000216 (0.05/231 comparisons).

### 4.3. Telomere Length Correlation Analysis

Telomere length (TL) ratio values were taken from Barthel et al., where detailed methods can be found [29]. LAML and mesothelioma (MESO) did not have telomere length data. Briefly, TL was quantified using TelSeq [119] for both TCGA tumor samples and either matched normal tissue (NT) or matched normal blood (NB) leukocytes. Tumor TL was divided by matched normal TL to generate a TL ratio. The majority of normal samples were NB rather than NT, except for kidney samples. Comparing within tissues found NT samples had significantly longer telomere length estimates in bladder, liver, lung, and stomach tissue than NB samples (Figure S35). Therefore, TL ratios calculated using NT were excluded, with the exception of kidney samples because of the small sample size among NB calculated TL ratios. Spearman’s correlation was used to identify any relationships with TERT isoform percentages. Using 7 isoforms and 31 cancer types, significance was determined with a Bonferroni corrected *p*-value of <0.000230 (0.05/217 comparisons).

### 4.4. Clustering Analysis 

Principal component analysis (PCA) was used for each cancer type and matched normal tissue to visualize the differences in isoform expression patterns. The variables included were the seven TERT transcripts’ TPM expression values. The PCA plots were made using the first two principal components. Uniform Manifold Approximation and Projection (UMAP) was used for clustering [120] with CCLE samples. UMAP projections require input variables for number of neighbors, number of components, and the distance metric. The “Manhattan” distance was used, defined as the sum of the lengths of line segments in a rectangular grid between points. A python script iterated through neighbor values of 2, 4, 8, 16, 32, 64, and 128, as well as component values of 2, 3, 4, 5, and 8 for each neighbor value to generate a maximum of 35 projections. A representative projection was chosen.

## 5. Conclusions

Sequencing costs have improved drastically over the last two decades, allowing for large scale projects such as TCGA, GTEx, and CCLE. With the plethora of “omic” data obtained, researchers have been empowered more than ever to elucidate the drivers of neoplastic transformation and progression, and provide clinically actionable guidance on diagnoses, prognoses, and therapeutic interventions. Herein, we initiate the characterization of the TERT transcriptome and outline possible research avenues in various normal and neoplastic tissue types. While descriptive in nature, our work emphasizes the cancer and cell-specific approach that future functional research should undertake. The next challenge will be integrating the multiple facets of TERT regulation and removing the ambiguity of isoform-level RNA sequencing while sustaining the large-scale feasibility necessary to uncover cell-type specific nuances and novel cancer therapeutic strategies.

## Figures and Tables

**Table 1:** Significant tumor-specific subtype-dependent differences in TERT expression or telomere length.

Tumour Type	Subtype	Category	Subtype Characterization ^1^	Potential Rationale ^1^
**BLCA**	Basal Squamous	↑ FL-TERT	High expression of stem-like markers [36]	Cellular potency is positively associated with TERT expression and TA activity [37,38]
	Neuronal	↑ αβγ-TERT	High frequency of RB1 mutations, proliferative cell state, increased expression of neural and neuroendocrine genes. Worst survival outcome [36].	N/A.
**BRCA**	C1	↓ TERT ↓ TL Ratio	Enriched for one or more positive hormone receptors and improved survival outcome [39].	ER promotes TERT expression by binding to TERT promoter [40]. However, ER expression is inversely correlated with TERT expression [41]. Possible negative feedback control system.
**COAD/STAD**	CIN	↑ TERT	Chromosomal instability [42].	Aneuploidy is associated with telomere deficiency [43,44,45,46,47,48] but increased TERT expression and TA [49]. Aneuploidy-induced telomere replication stress can be alleviated by TA [50].
	GS	↓ TERT	Genome stability [42].	Aneuploidy is associated with telomere deficiency [43,44,45,46,47,48] but increased TERT expression and TA [49]. Aneuploidy-induced telomere replication stress can be alleviated by TA [50].
**HNSC**	Basal	↓ FL-TERT ↓ TERT ↓ TL Ratio	Enriched NOTCH1 inactivation, decreased SOX2 expression and HRAS-CASP8 co-mutations [51].	NOTCH1 activation results in increased TERT expression and TA in dental follicle cells [52].
**LUAD**	C2	↑ FL-TERT	Exclusively PP tumors. Enriched for KRAS mutations and STK11 inactivation [53].	KRAS mutation increases TERT expression, TA and TL in immortalized bronchial epithelial and lung adenocarcinoma cells [54].
**LUSC**	Primitive	↑ FL-TERT Isoform %	Limited differentiating qualities [55]	N/A
**SARC**	C1	↓ TERT	Primarily LMS tumors with higher frequency of RB1 mutations and no association between TL and ATRX alterations, unlike UPS and MFS tumors [56].	UPS and MFS employ ALT via ATRX alterations, but LMS potentially does via loss of RB1 [29,57]
	C2	↑ TERT	Primarily DDLPS tumors. Sub-cluster of DDLPS tumors based on somatic copy number alteration found to have worse survival and TERT amplification [56].	TERT expression is gene-dosage dependent [58]. TERT amplification events are rare but is associated with the highest TA [29].
	C4	↑ TERT	Exclusively SS tumors. High FGFR3, miR-183 expression and PDE4A promoter methylation [56].	FGFR3 gain-of-function mutations and TERT promoter mutations significantly co-occur in bladder cancer [59].
**TGCT**	Embryonal	↑ TERT	NSE tumor subtype that arises from early gonadal stem cells and exhibits gonadal morphology [60].	NSE tumors have increased TERT expression, TL and stemness gene expression [61]. TERT expression and TA decline with TCGT differentiation status [62].
**THYM**	C1, C3	↑ TERT	Higher lymphocyte content [63].	Normal lymphocytes have endogenous TERT expression [64].

^1^ TA = telomerase activity; TL = telomere length; ATRX = α-thalassaemia/mental retardation syndrome X-linked; DAXX = death domain-associated protein; ALT = alternative lengthening of telomeres; RB1 = retinoblastoma protein; ER = estrogen receptor; GI = gastrointestinal; PP = proximal proliferative; LMS = smooth muscle differentiated leiomyosarcoma; UPS = undifferentiated pleomorphic sarcoma; MFS = myxofibrosarcoma; DDLPS = dedifferentiated liposarcoma; SS = synovial sarcoma; SE = seminoma; NSE = non-seminoma. ↑ TERT: increased TERT; ↓ TERT: decrease TERT.

**Table 2:** Cancer cell lines clustered to related tumors based on TERT isoform expression patterns.

Category	Tumor Type	Cancer Cell Line ^1^
*Biliary Tract*	CHOL	HUCCT1 ^†^, SNU869 ^†^
*Hematopoietic & Lymphoid Tissue*	LAML DLBC	OCIAML5 ^†^ SUPM2, KMH2, HL60, RS411, LOUCY
*Central Nervous System & Autonomic Ganglia*	LGG GBM PCPG	IOMMLEE, TM31 ^†^, LNZ308 IOMMLEE, TM31, LNZ308 TM31, LNZ308, DKMG
*Breast*	BRCA	JIMT1, BT549, MDAMB231 ^P^
*Large Intestine*	COAD	CL11 ^WT^, SNU1197 ^WT^, CW2 ^WT^
*Endometrium*	CESC UCEC UCS	JHUEM2, JHUEM7 JHUEM2, JHUEM7 JHUEM2, JHUEM7
*Esophagus*	ESCA	TE11 ^WT †^, KYSE510, KYSE410 ^P^, COLO680N
*Kidney*	KIRC KIRP KICH	CAKI1 ^WT^ CAKI1 ^WT^ CAKI1 ^WT^
*Liver*	LIHC	HEPG2 ^P,†^, LI7
*Lung*	LUAD LUSC	NCIH2030 ^WT^, DMS152, NCIH727 ^WT^, MORCPR NCIH596 ^WT^, NCIH2228 ^WT^, NCIH1755 ^WT^, CORL47 ^WT^
*Ovary*	OV	COV362 ^WT †^, JHOM1, TOV112D, OVK18 ^WT^
*Pancreas*	PAAD	CAPAN1 ^WT †^
*Pleura*	MESO	NCIH2052 ^P^, ISTMES2 ^WT †^
*Prostate*	PRAD	PC3 ^WT^
*Skin*	SKCM	SKMEL1, SKMEL28 ^P^, HT144 ^P^, HS695T ^P^
*Soft Tissue*	SARC UCS	EW8, CADOES1 ^WT^, RD EW8, CADOES1 ^WT^, RD
*Stomach*	STAD	MKN7 ^WT^, IM95 ^WT^, OCUM1
*Thyroid*	THCA	TT2609C02
*Urinary Tract*	BLCA	UBLC1, UMUC2, CAL29 ^†^, RT4 ^†^

^1^ “WT” indicates wild-type TERT promoter status; “P” indicates mutated TERT promoter status; no superscript indicates TERT promoter status was not profiled [65]. ^†^ Indicates cell lines recommended by TCGA-110-CL [67].

## Data Availability

The data presented in this study are available from online databases or specific papers, as outlined in the methods section and Supplementary Materials (Table S1).

## Supplementary Materials

The following are available online at https://www.mdpi.com/article/10.3390/cancers13081853/s1. Figure S1: Most TCGA tumour types have the majority of samples expressing TERT. Figure S2: Most GTEx normal tissue types have the majority of samples not expressing TERT. Figure S3: Total TERT expression is highest in basal ganglia brain structures in normal (GTEx) tissues. Figure S4: Cancers primarily express FL-TERT or TERT_238.6 followed by TERT_656.1. Figure S5: Normal tissues primarily express TERT_238.6 followed by different isoforms depending on tissue type. Figure S6: Normal tissue types with greater total TERT expression approximate the isoform correlation framework observed in tumour tissues. Figures S7–S12: TERT isoform’s relationships to telomere length ratio across cancer types. Figure S13–S30: Tissue type-specific UMAP projections of TCGA, GTEx and CCLE samples. Figure S31: NOVA1 expression is significantly higher in the FL-TERT expressing group of only LUAD. Figure S32: PTBP1 expression is significantly higher in the FL-TERT expressing group of many cancer types. Figure S33: Data filtering workflow from the total TOIL recomputed dataset to only TERT-positive TCGA and matched GTEx samples, Figure S34: Ensembl annotations of TERT isoforms used in Toil recomputed dataset (Build 93) and CCLE dataset (Build 75). Figure S35: Comparison of telomere length from normal peripheral blood and normal adjacent tissue for each TCGA tissue type. Table S1: Selected subtypes for each TCGA cancer type identified through independent molecular profiling. Table S2: Telomerase activity score signature score gene list.
