# Supplementary material for: Analysis of TERT Isoforms across TCGA, GTEx and CCLE Datasets
Source: Cancers (Basel). 2021 Apr 13;13(8):1853. doi: 10.3390/cancers13081853 (PMC8070023; doi:10.3390/cancers13081853)
Supplement: Supplementary file 1 [file cancers-13-01853-s001.zip › Supplementary Figures and Tables.docx]

**SUPPLEMENTARY FIGURES AND TABLES**

**
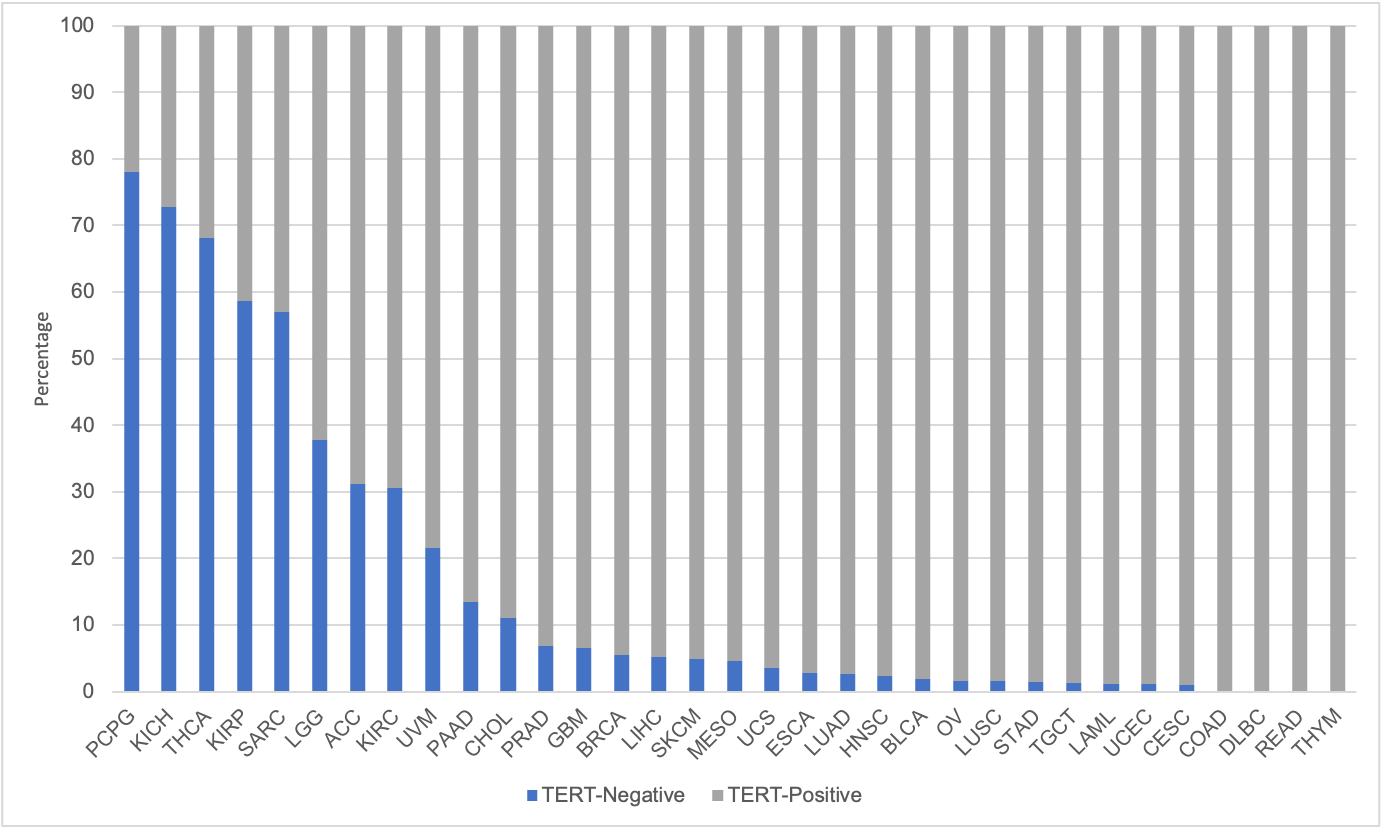
**

**Figure S1. Most TCGA tumour types have the majority of samples expressing TERT.** Stacked bar plot summarizing the frequency of TERT-negative samples in each cancer type. Blue colour representing TERT-negative samples and gray representing TERT-positive samples.

**
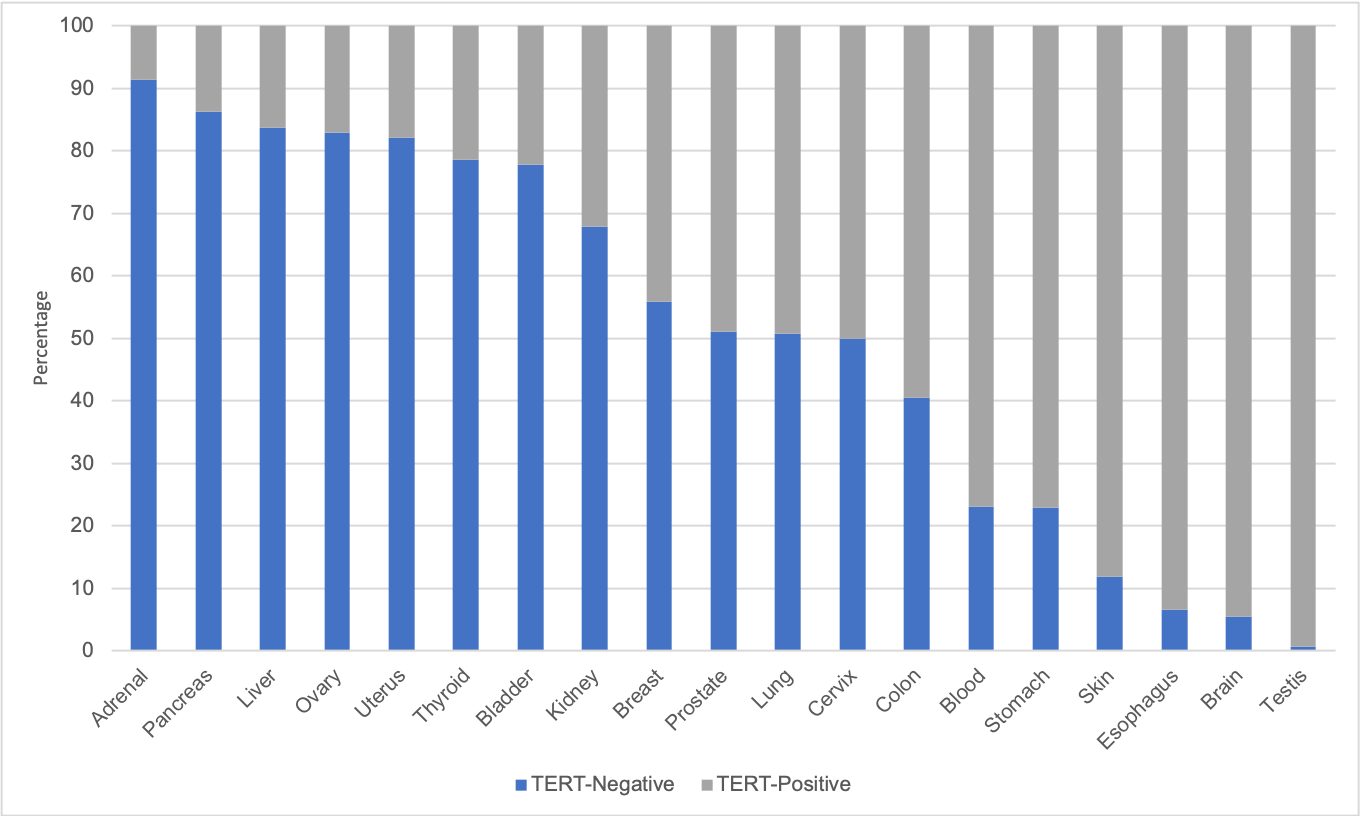
**

**Figure S2. Most GTEx normal tissue types have the majority of samples not expressing TERT.** Stacked bar plot summarizing the frequency of TERT-negative samples in each cancer type. Blue colour representing TERT-negative samples and gray representing TERT-positive samples.


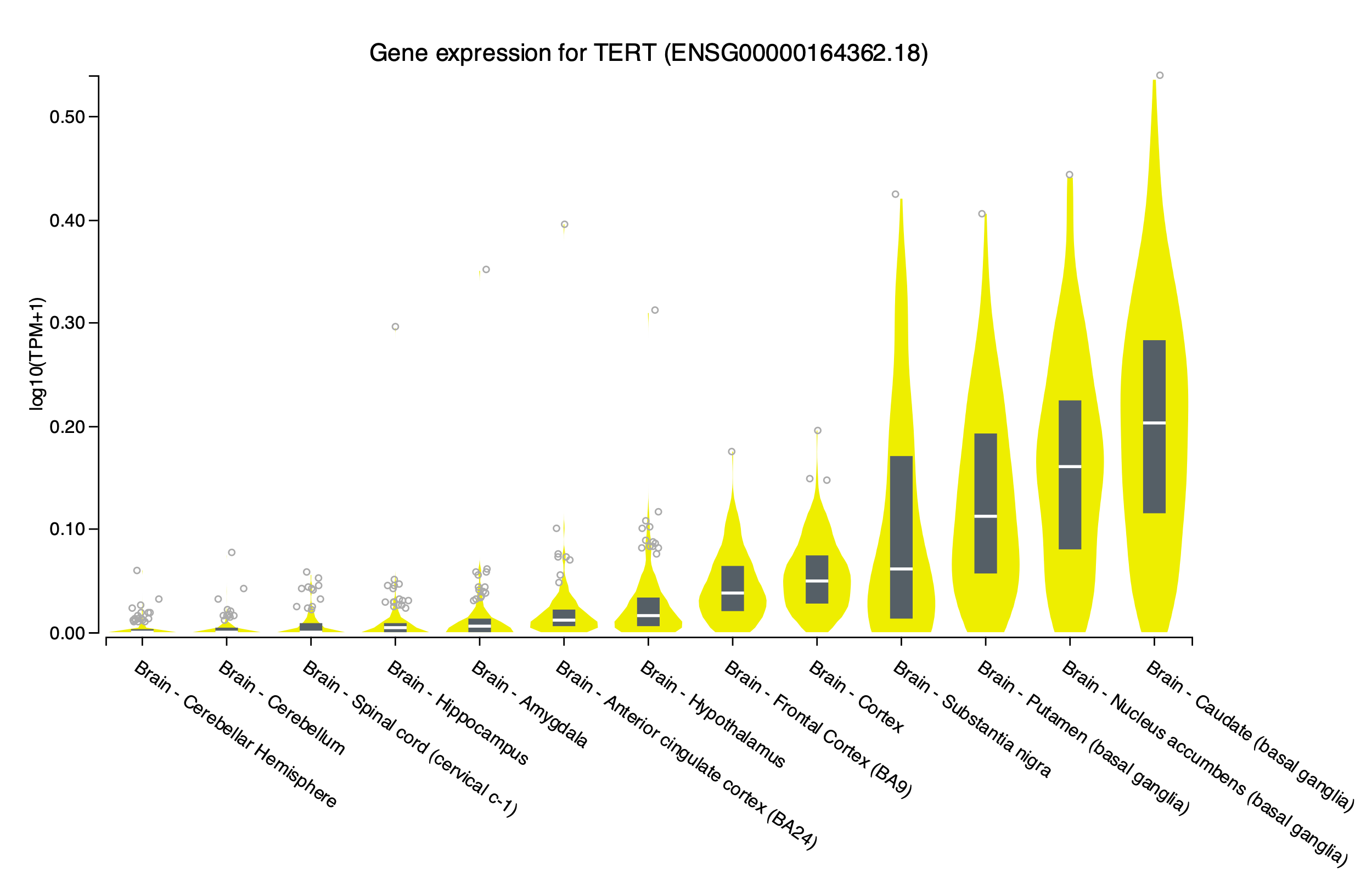


**Figure S3. Total TERT expression is highest in basal ganglia brain structures in normal (GTEx) tissues.** Total TERT expression in log_10_(TPM+1) scale, across normal human brain tissue subtypes. Expression values are shown in TPM, calculated from a gene model with isoforms collapsed to a single gene. No other normalization steps have been applied. Box plots are shown as median and 25th and 75th percentiles; points are displayed as outliers if they are above or below 1.5 times the interquartile range. Made using the GTEx portal (https://gtexportal.org/).

**Figure S4. TCGA cancer-type specific TERT isoform expression.** Primary TERT isoforms expressed in all cancer types were full-length TERT (TERT_581.9) and TERT_238.6. TERT_656.1 was the third most expressed transcript in every cancer type. Following this, the general expression pattern was ß-TERT (not expressed in PCPG), αßγ-TERT (not expressed in CHOL), γ-TERT (not expressed in 5/33 cancer types) and finally TERT_877.1 (not expressed in 10/33 cancer types). Box plots boxes denote the inter-quartile range as well as a bolded line representing the median. Extending from the boxes are minimum and maximum lines calculated from 1.5 times the interquartile range. Points outside this range are considered outliers. Within each box is a red point signifying the mean, as well as lines extending from this point representing a 95% confidence interval. Expression transcript per million (TPM) values were transformed by a log2(TPM + 0.001) equation.

**Figure S5. GTEx tissue-type specific TERT isoform expression.** The primary TERT isoform expressed in all normal tissue types was TERT_238.6. Following this FL-TERT was commonly the second highest expressed isoform. The exceptions were ovarian (expressed ß-TERT higher), adrenal tissue (expressed αßγ-TERT higher and did not express FL-TERT), cervical tissue (expressed TERT_656.1 higher), pancreatic tissue (expressed both ß-TERT and TERT_656.1 higher) and liver tissue (expressed both ß-TERT and TERT_656.1 higher and did not express FL- TERT). Box plots boxes denote the inter-quartile range as well as a bolded line representing the median. Extending from the boxes are minimum and maximum lines calculated from 1.5 times the interquartile range. Points outside this range are considered outliers. Within each box is a red point signifying the mean, as well as lines extending from this point representing a 95% confidence interval. Expression transcript per million (TPM) values were transformed by a log2(TPM + 0.001) equation.

COAD

BRCA

ACC

Adrenal

Breast

Colon


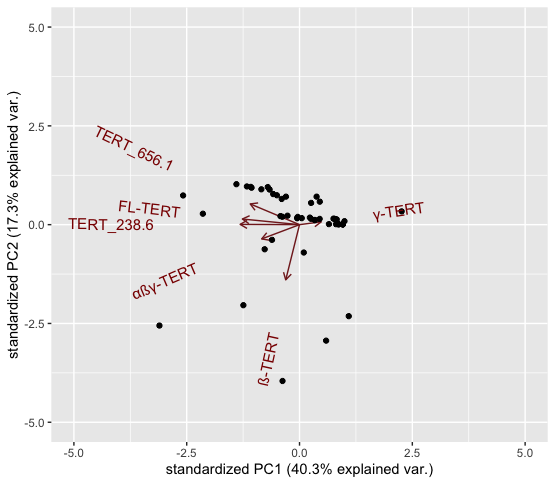

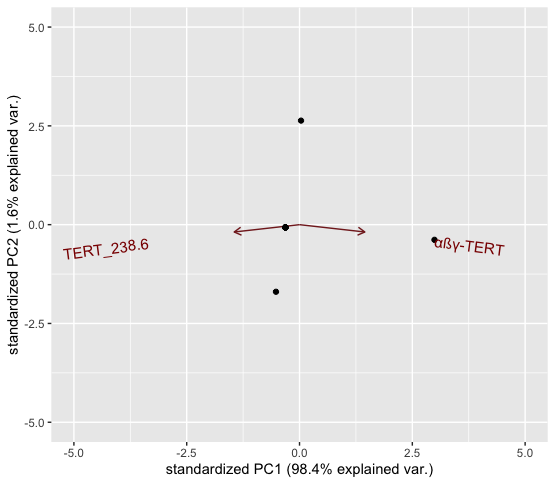


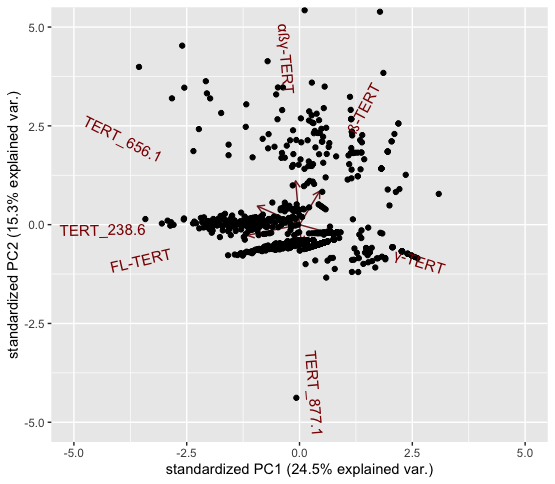

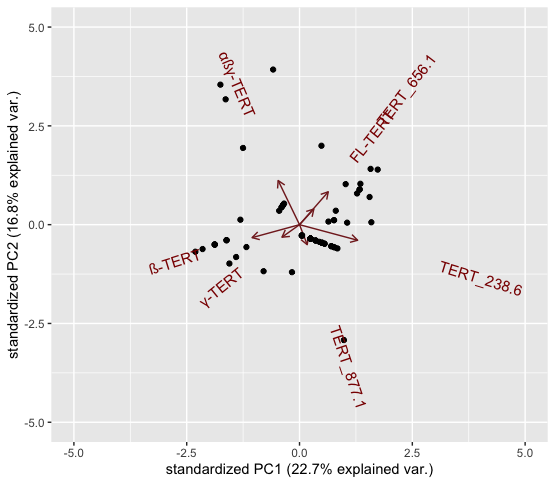


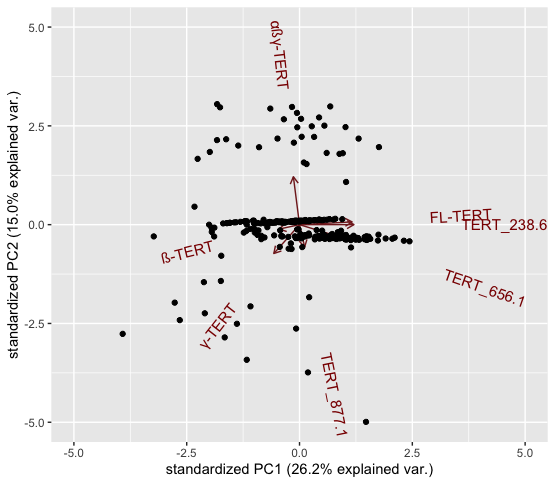

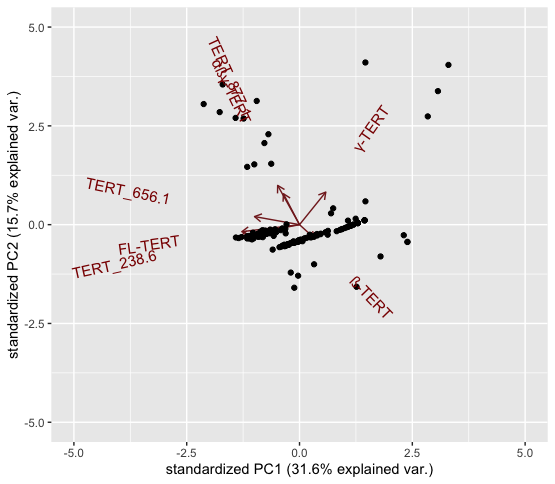


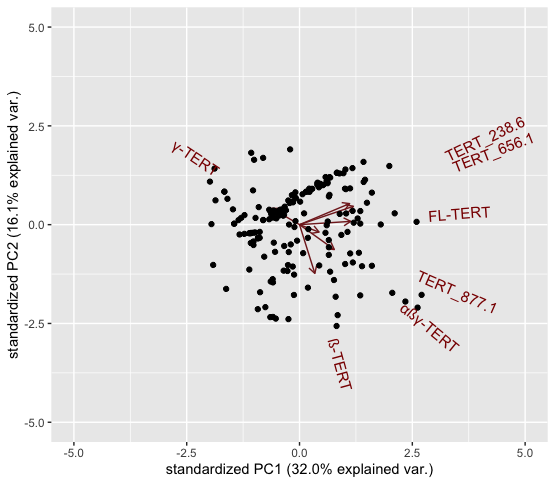

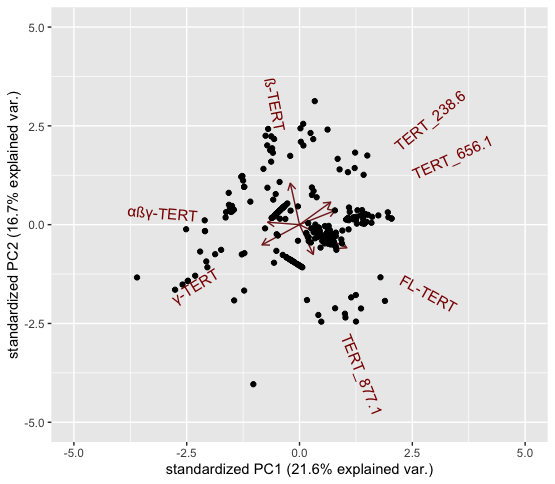


Ovary

Liver

Esophagus

ESCA

LIHC

OV


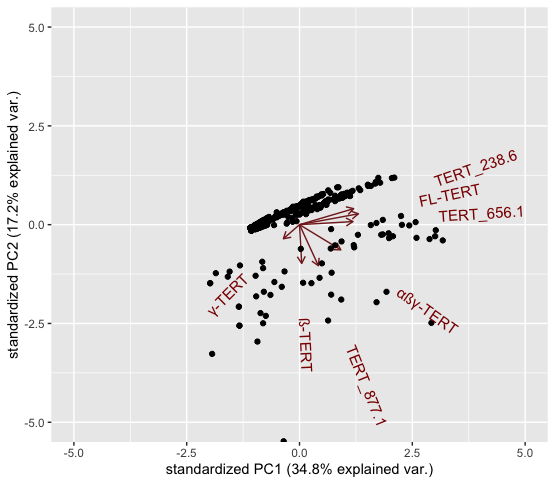

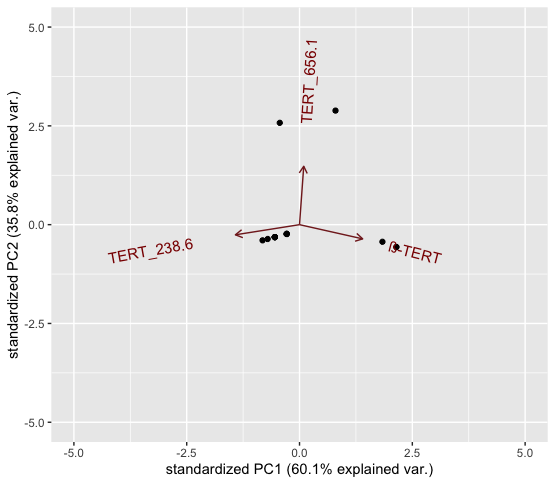


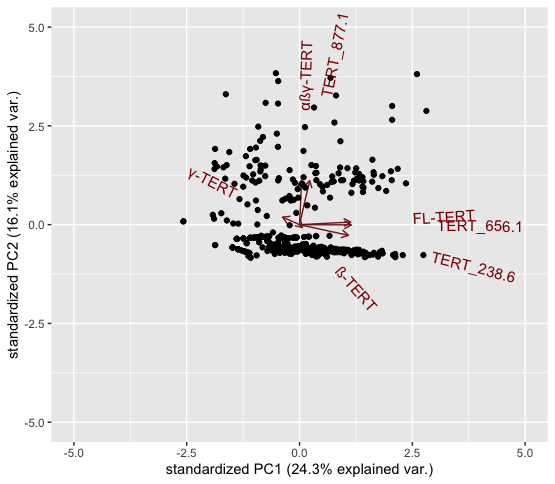

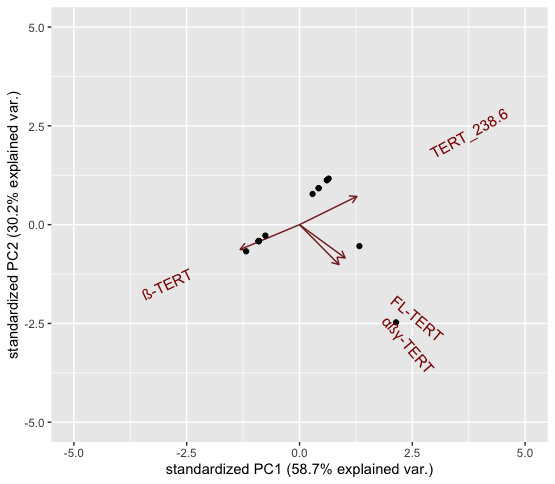


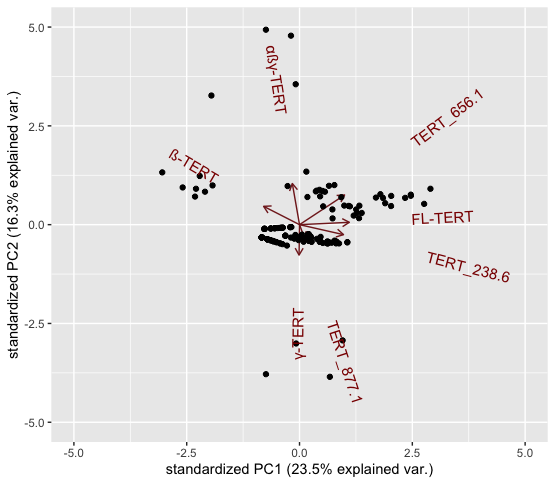

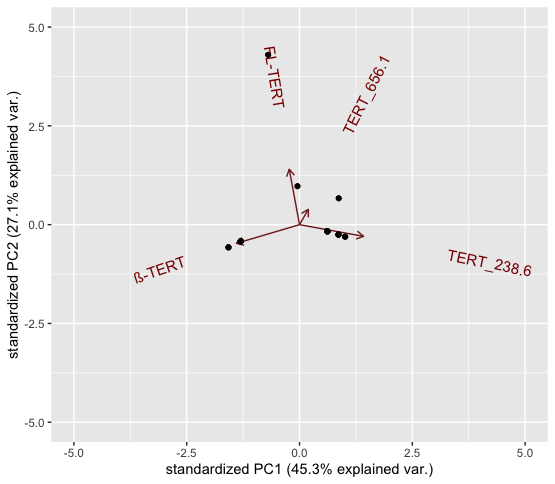


SKCM

PRAD

PAAD

Pancreas

Prostate

Skin


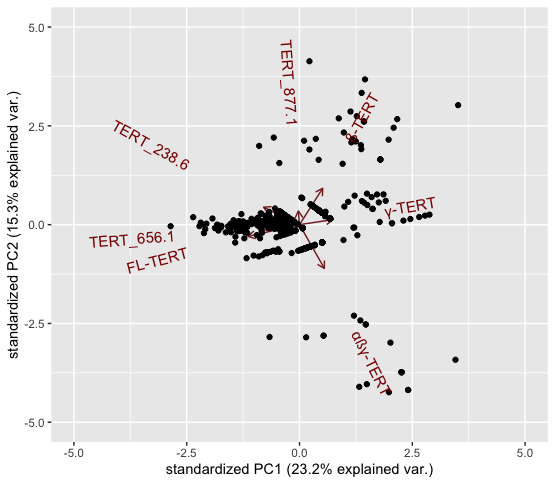

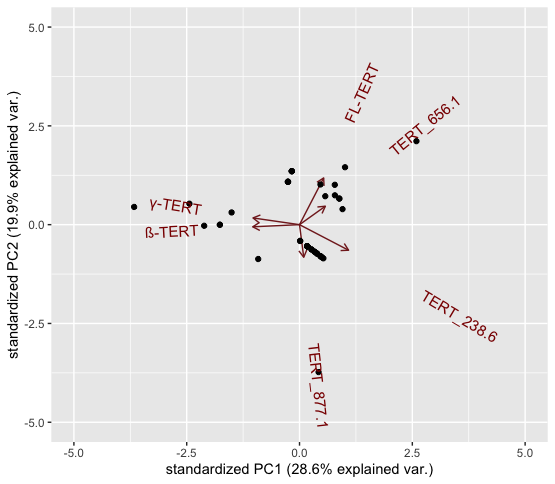


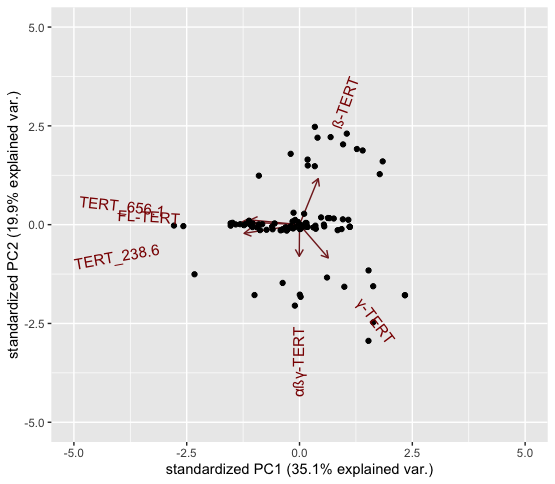

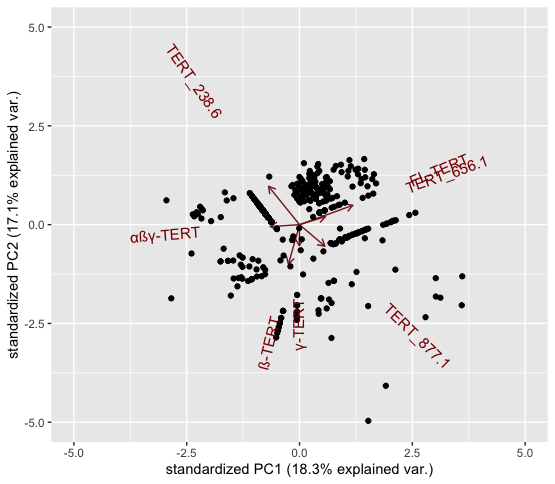


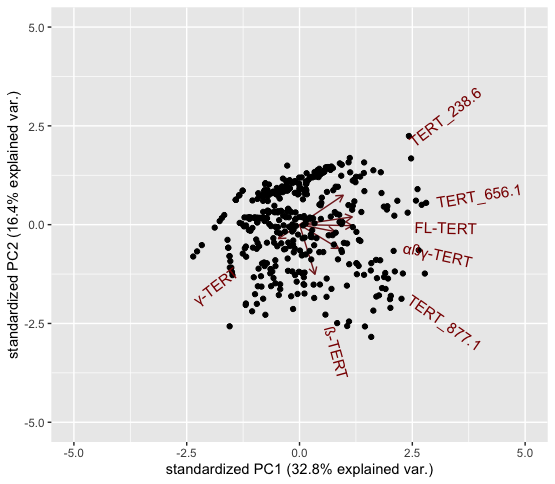

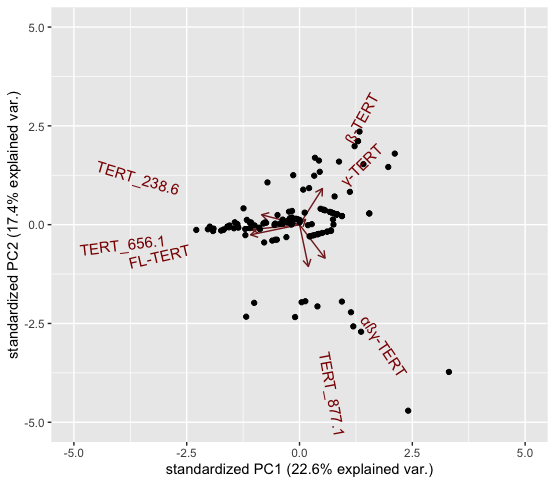


THCA

TGCT

STAD

Stomach

Thyroid

Testis


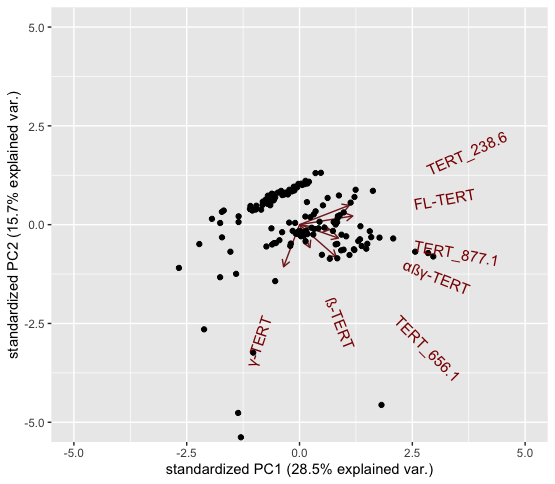

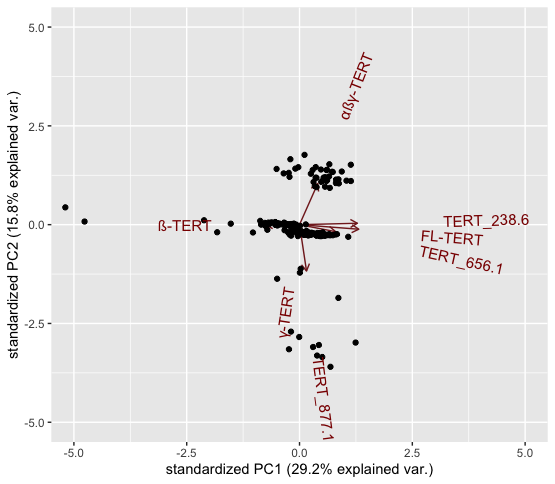


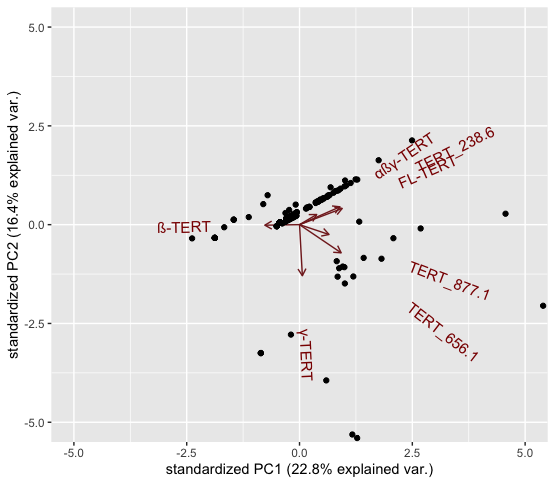

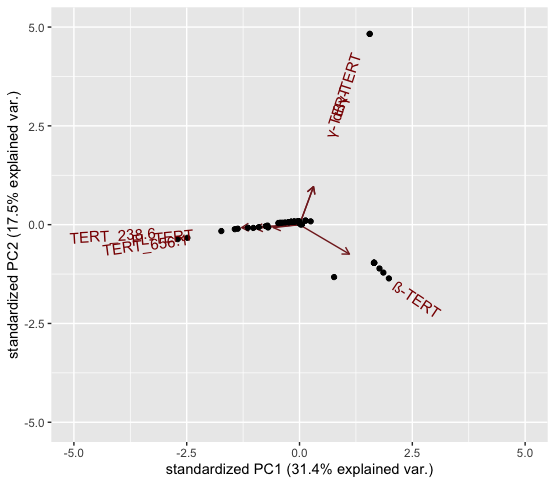


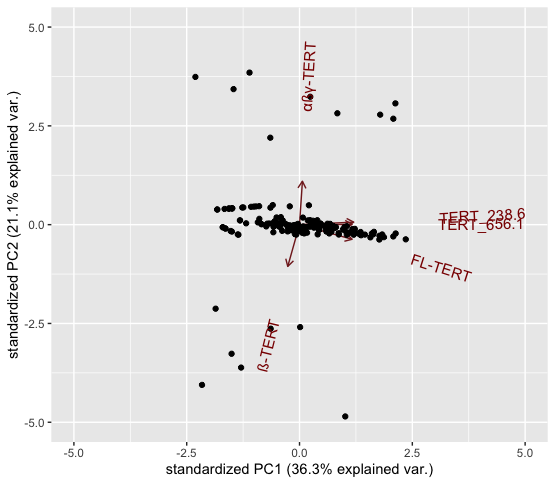

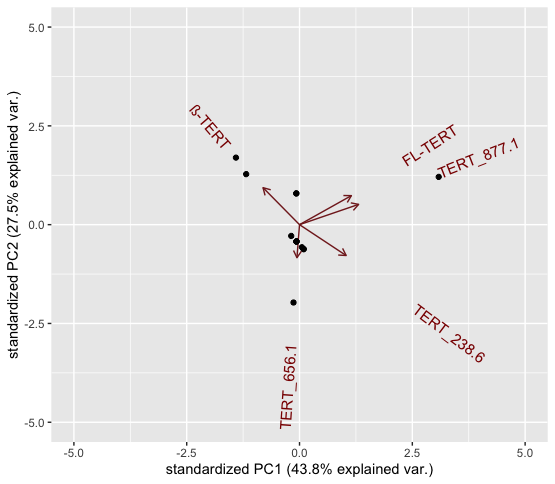


Uterus

UCEC

Blood

LUAD

LUSC

GBM

LGG

DLBC

LAML

Brain

Lung


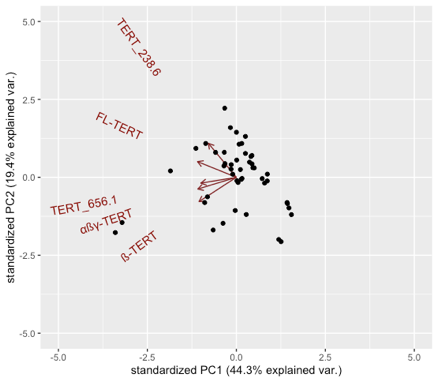

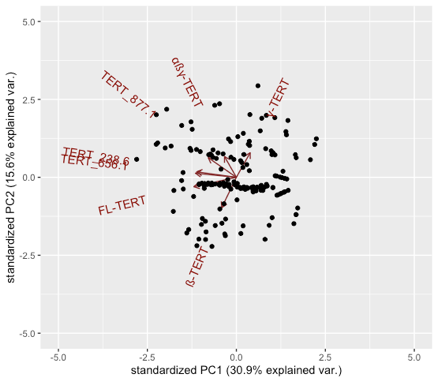

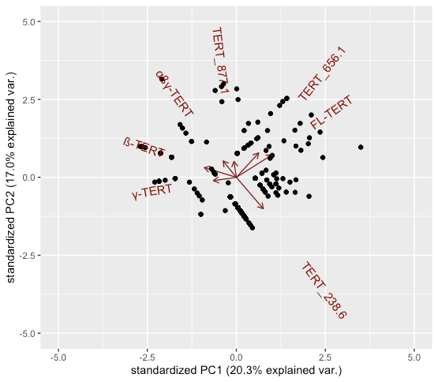


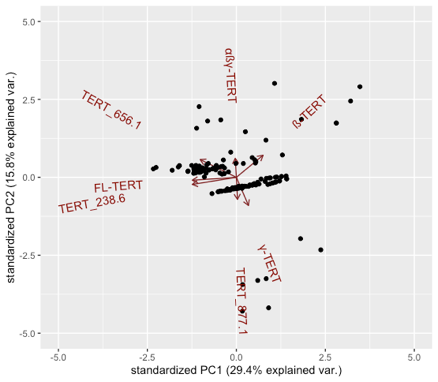

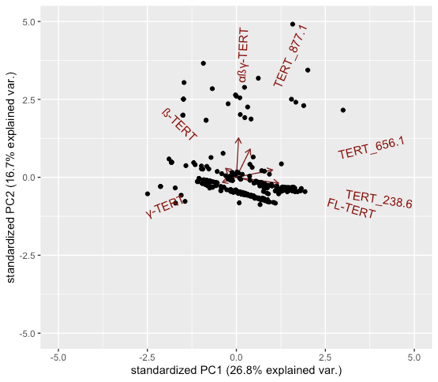

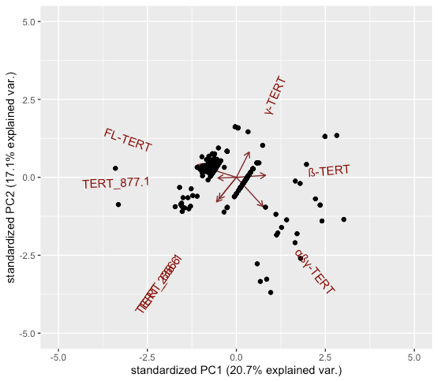


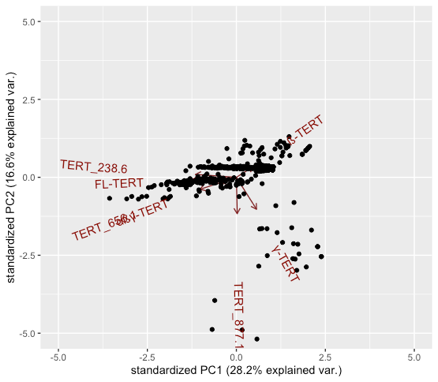

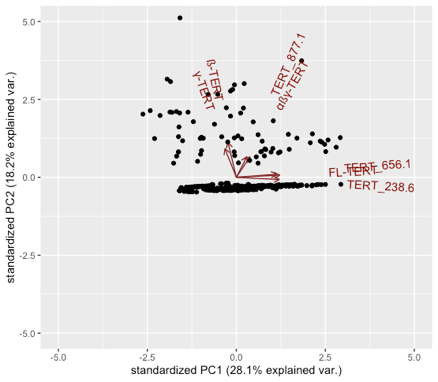

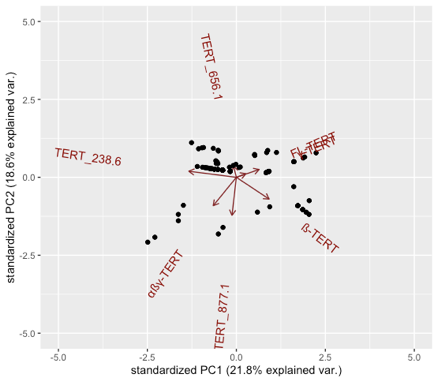


**Figure S6. Normal tissue types with greater total TERT expression approximate the isoform correlation framework observed in tumour tissues.** PCA biplots for each tumour and normal tissue type are plotted using the first two principal components and show the loading of each TERT isoform variable (arrow) expressed. The arrow length approximates the variance of the variable. The angle between arrows approximates the correlations between variables. Such that parallel arrows in the same direction have positive correlations, perpendicular arrows have no/weak correlations, and parallel arrows in the opposite direction have negative correlations.

**Figure S7. TERT_238.6 expression correlation to telomere length ratio.** Telomere length ratios and TERT_238.6 expression were log_10_ and log_2_(TPM + 0.001) transformed, respectively. Spearman correlations were computed, and significance determined using a Bonferroni corrected p-value of < 0.000230 (0.05/217 comparisons). Significant correlations are highlighted with a red border.

**Figure S8. ß-TERT expression correlation to telomere length ratio.** Telomere length ratios and ß-TERT expression were log_10_ and log_2_(TPM + 0.001) transformed, respectively. Spearman correlations were computed, and significance determined using a Bonferroni corrected p-value of < 0.000230 (0.05/217 comparisons). Significant correlations are highlighted with a red border.

**Figure S9. αßγ-TERT expression correlation to telomere length ratio.** Telomere length ratios and αßγ-TERT expression were log_10_ and log_2_(TPM + 0.001) transformed, respectively. Spearman correlations were computed, and significance determined using a Bonferroni corrected p-value of < 0.000230 (0.05/217 comparisons). Significant correlations are highlighted with a red border.

**Figure S9. γ-TERT expression correlation to telomere length ratio.** Telomere length ratios and **γ**-TERT expression were log_10_ and log_2_(TPM + 0.001) transformed, respectively. Spearman correlations were computed, and significance determined using a Bonferroni corrected p-value of < 0.000230 (0.05/217 comparisons). Significant correlations are highlighted with a red border.

**Figure S11. TERT_656.1 expression correlation to telomere length ratio.** Telomere length ratios and TERT_656.1 expression were log_10_ and log_2_(TPM + 0.001) transformed, respectively. Spearman correlations were computed, and significance determined using a Bonferroni corrected p-value of < 0.000230 (0.05/217 comparisons). Significant correlations are highlighted with a red border.

**Figure S12. TERT_877.1 expression correlation to telomere length ratio.** Telomere length ratios and TERT_877.1 expression were log_10_ and log_2_(TPM + 0.001) transformed, respectively. Spearman correlations were computed, and significance determined using a Bonferroni corrected p-value of < 0.000230 (0.05/217 comparisons). Significant correlations are highlighted with a red border.


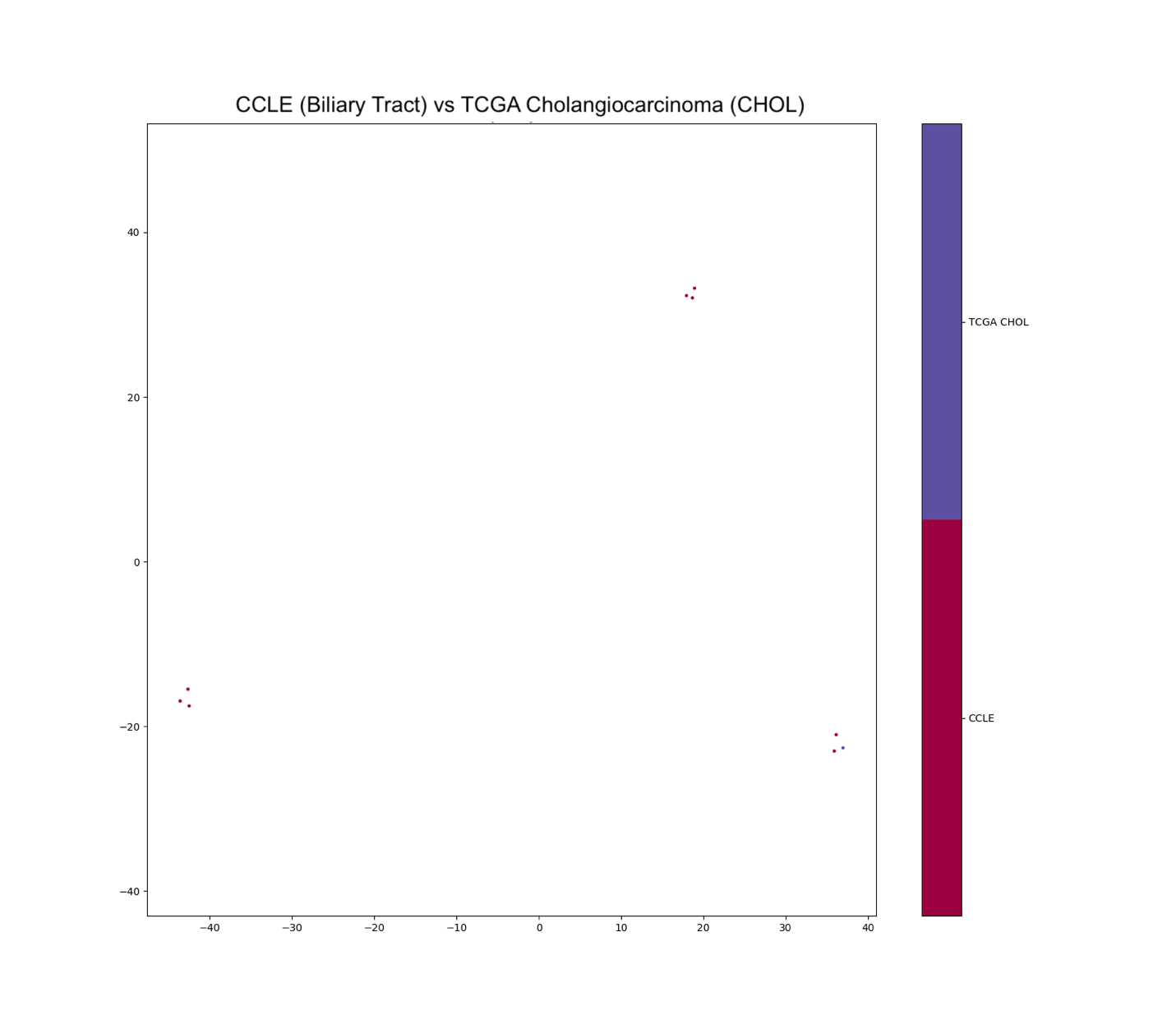

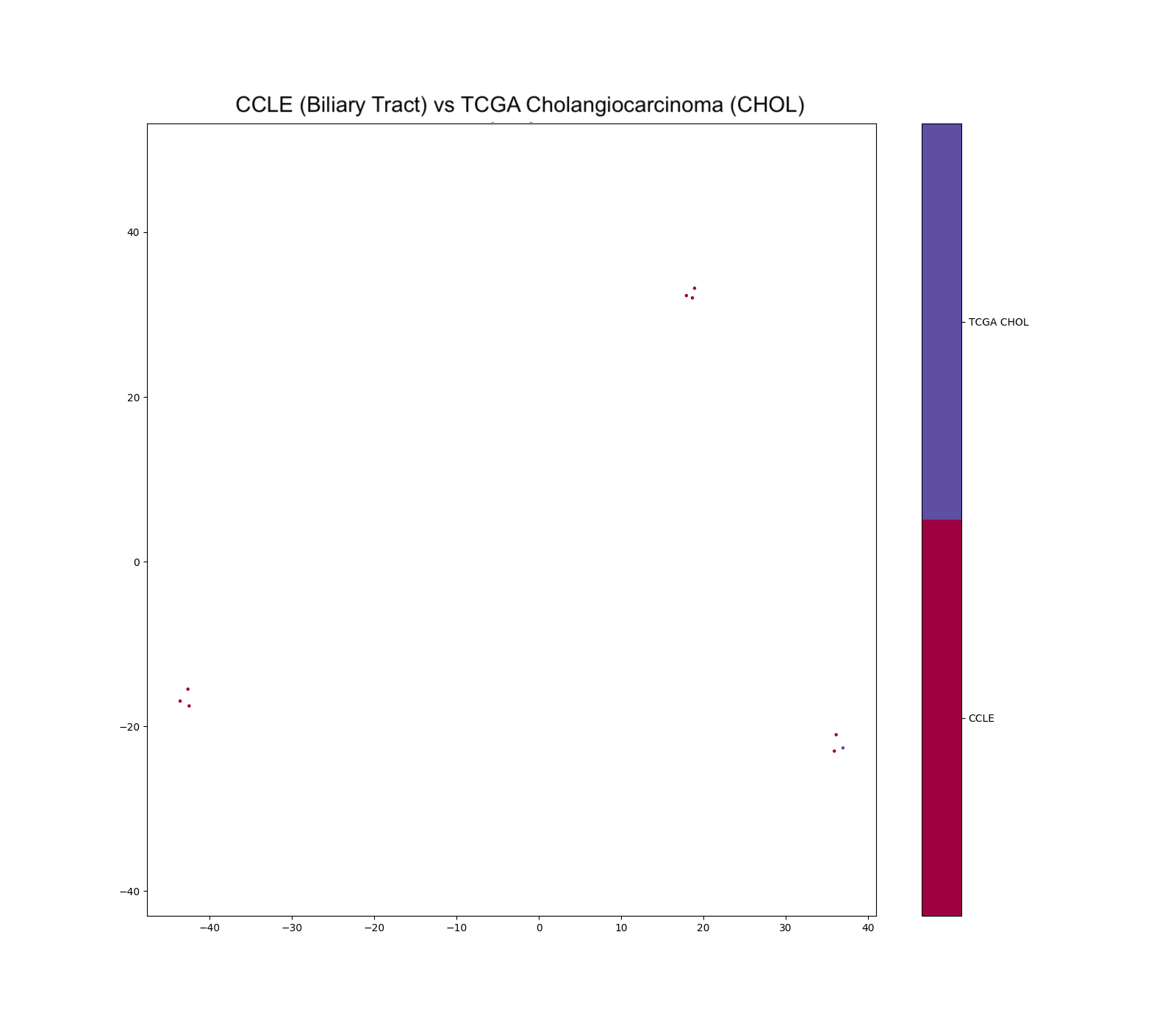


HUCCT1

SNU869

**Figure S13. UMAP projection of biliary tract CCLE cell lines and TCGA Cholangiocarcinoma (CHOL) using TERT isoform expression.** Cell lines HUCCT1 and SNU869 were closest to average isoform percentage from CHOL tumours. Dashed-line box indicates a zoomed in region of interest with text labels of cell lines. Cell line TERT promoter status taken from *Ghandi et al., 2019.* Superscript “P” indicated TERT promoter mutation, superscript “WT” indicates wild-type TERT promoter, and no superscript indicates no data available. Parameters: Manhattan distance, 2 neighbours and 3 components.


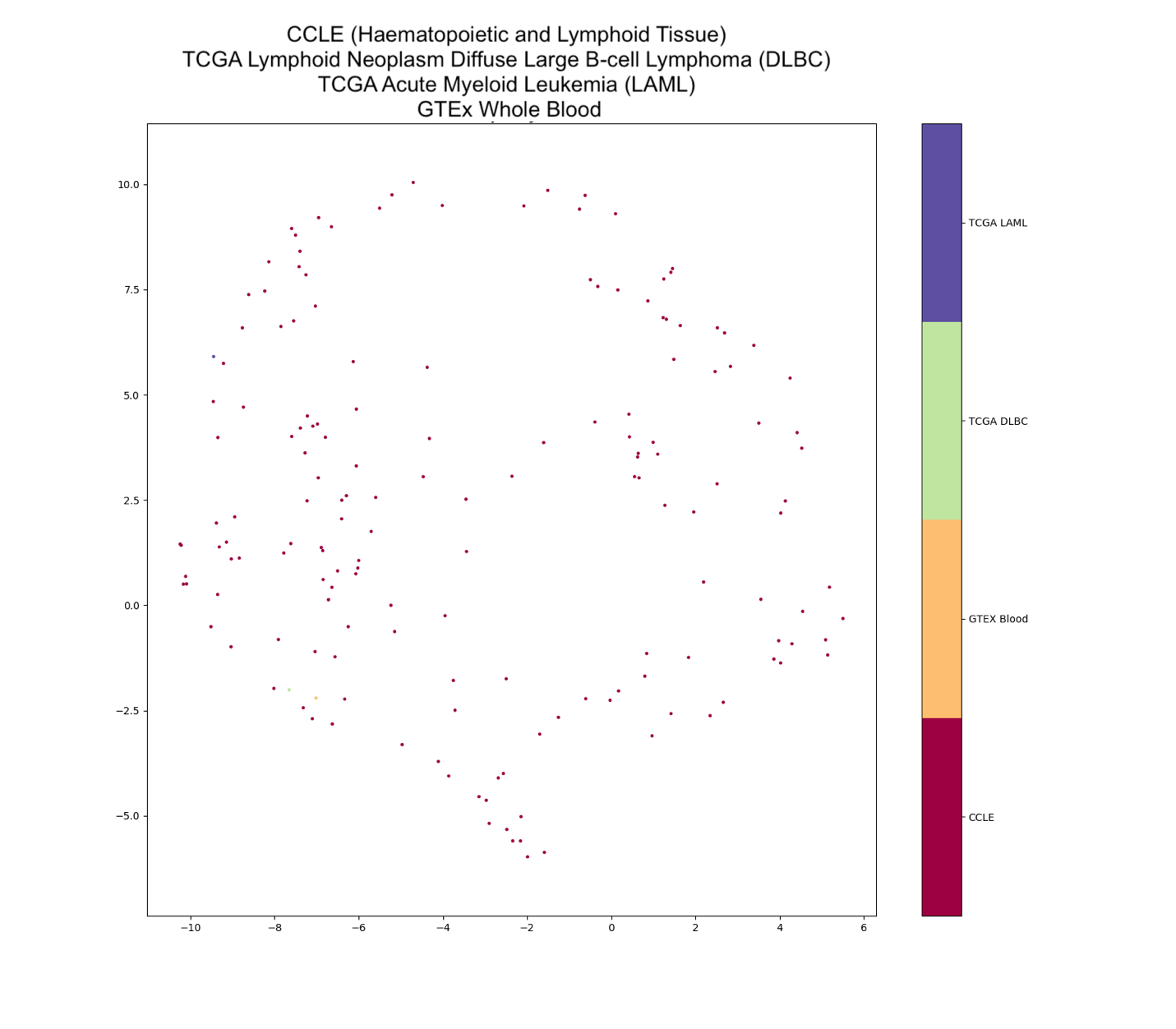

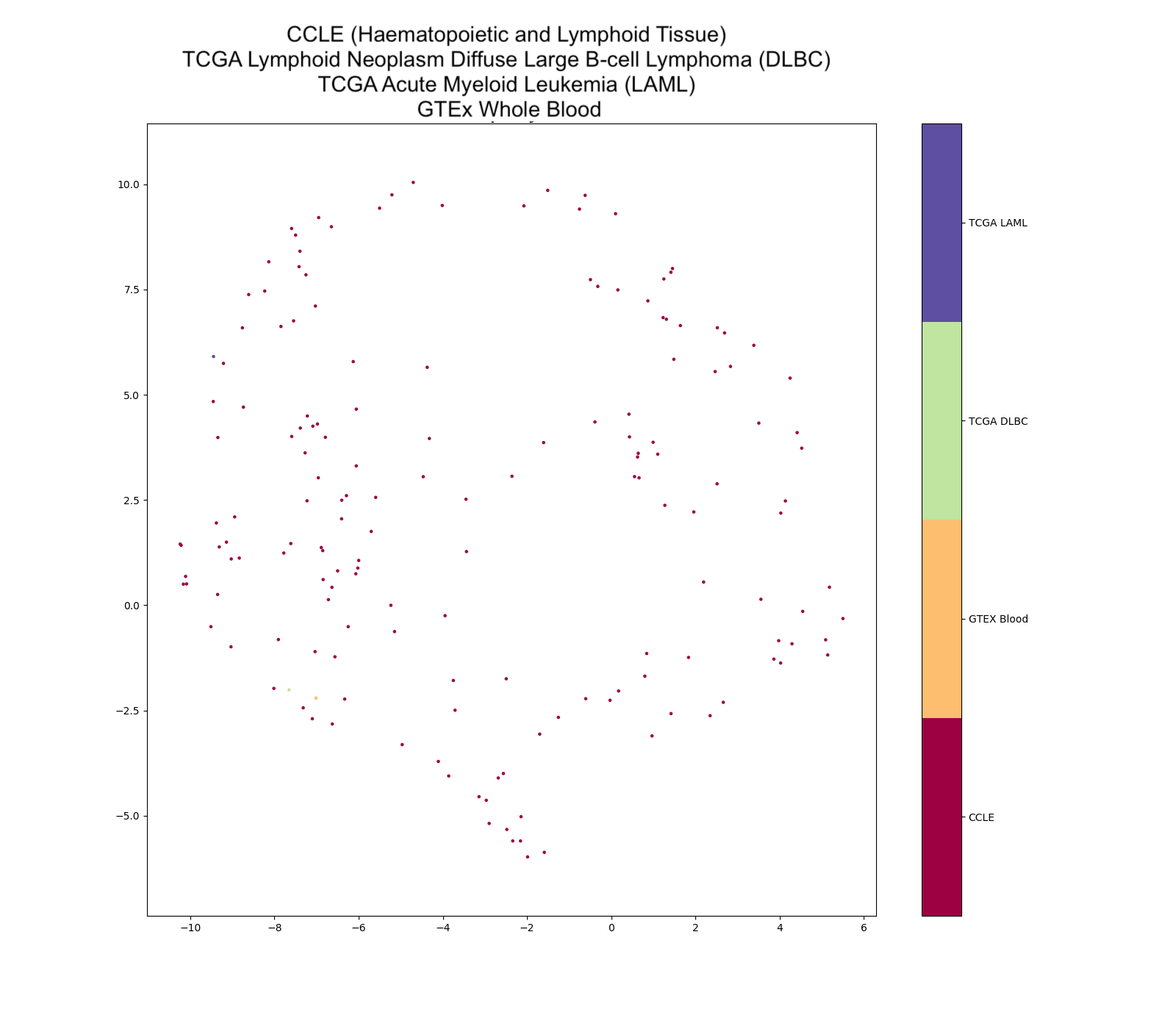

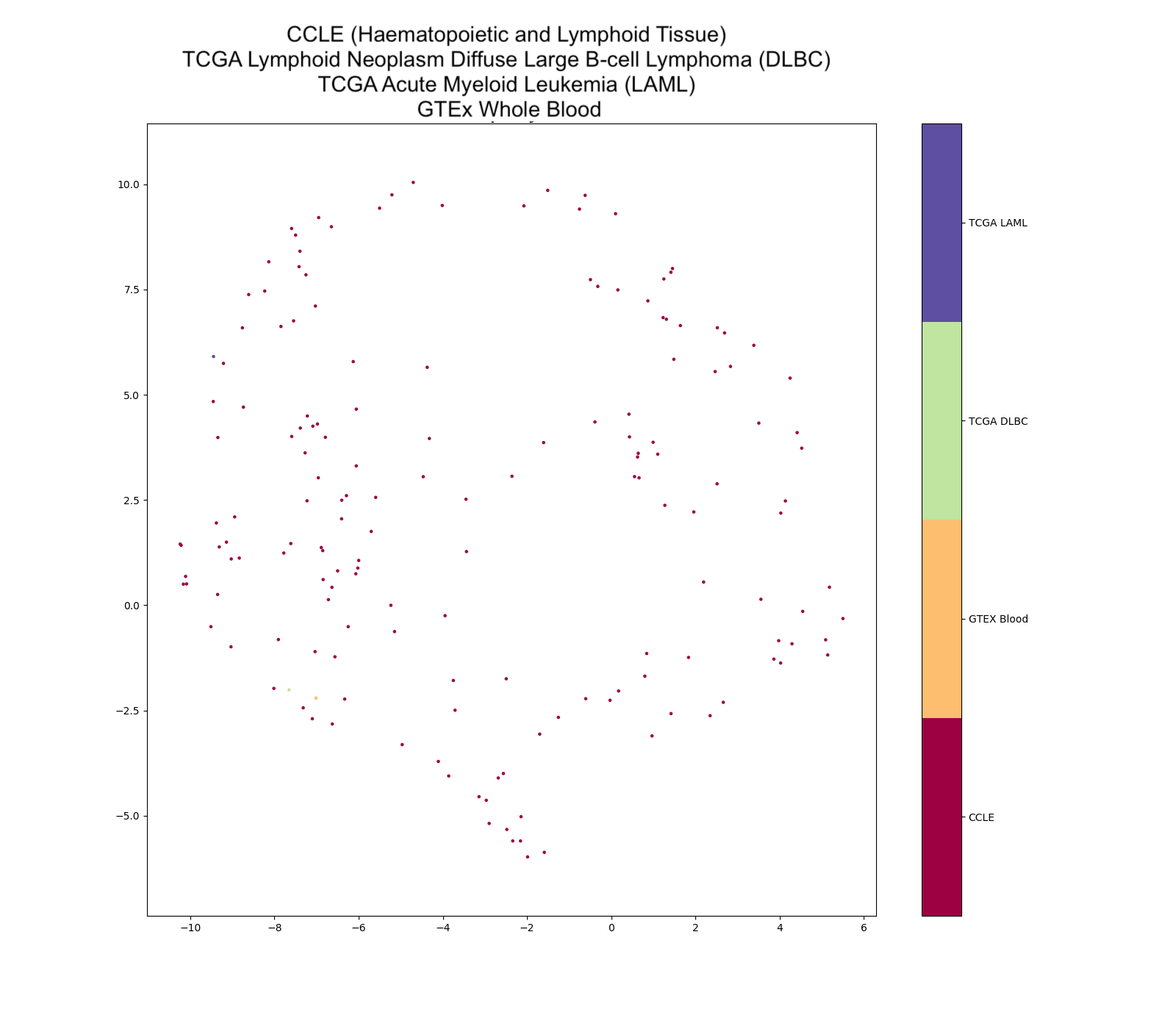


SUPM2

RS411

KMH2

HL60

LOUCY

OCIAML5

**Figure S14. UMAP projection of haematopoietic and lymphoid tissue CCLE cell lines, TCGA lymphoid neoplasm diffuse large B-cell lymphoma (DLBC), TCGA acute myeloid leukemia (LAML) and GTEx whole blood using TERT isoform expression.** Cell line OCIAML5 was closest to average isoform percentage from TCGA LAML. Cell lines SUPM2, KMH2, HL60, RS411 and LOUCY were closest to average isoform percentage from TCGA DLBC and GTEx blood. Cell line TERT promoter status taken from *Ghandi et al., 2019.* Superscript “P” indicated TERT promoter mutation, superscript “WT” indicates wild-type TERT promoter and no superscript indicates no data available. Dashed-line box indicates a zoomed in region of interest with text labels of cell lines. Parameters: Manhattan distance, 16 neighbours and 4 components.


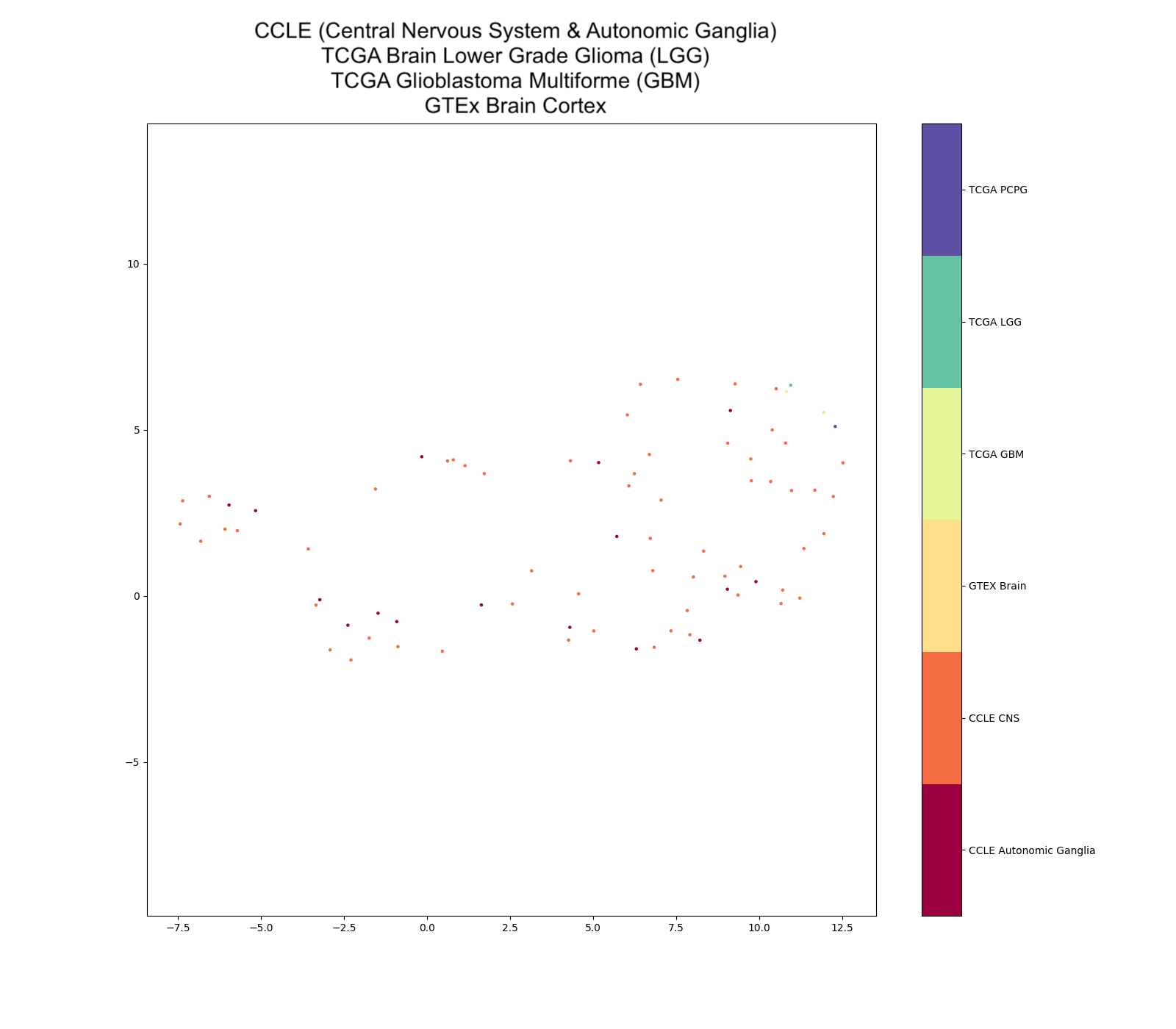

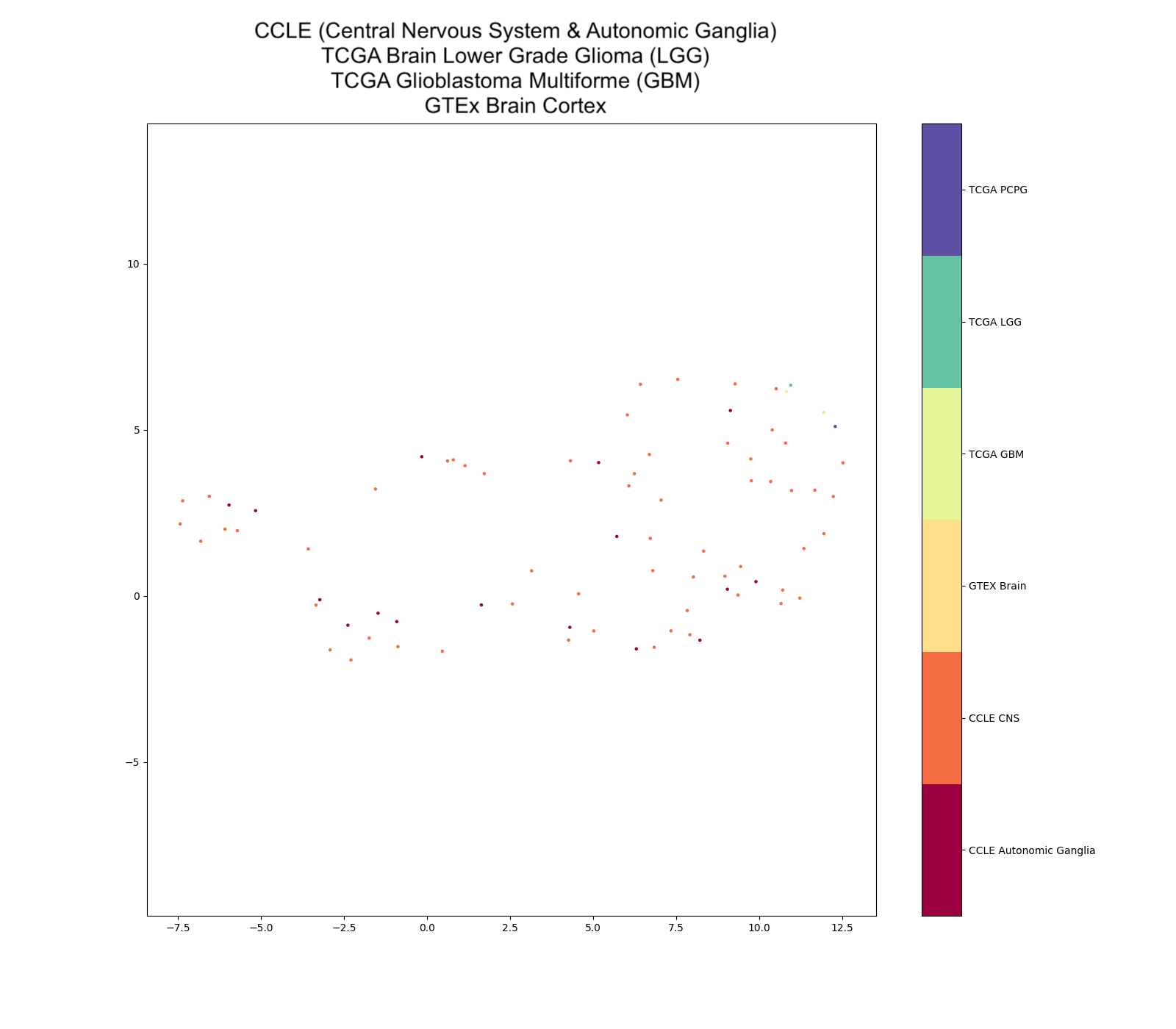


DKMG

LNZ308

TM31

IOMMLEE

**Figure S15. UMAP projection of central nervous system and autonomic ganglia CCLE cell lines, TCGA brain lower grade glioma (LGG), TCGA glioblastoma multiforme (GBM) and GTEx brain cortex using TERT isoform expression.** Cell line IOMMLEE was closest to average isoform percentage from TCGA LGG and GBM. Cell lines TM31, LNZ308 and DKMG were closest to average isoform percentage from TCGA PCPG and GTEx brain. Cell line TERT promoter status taken from *Ghandi et al., 2019.* Superscript “P” indicated TERT promoter mutation, superscript “WT” indicates wild-type TERT promoter and no superscript indicates no data available. Dashed-line box indicates a zoomed in region of interest with text labels of cell lines. Parameters: Manhattan distance, 8 neighbours and 4 components.


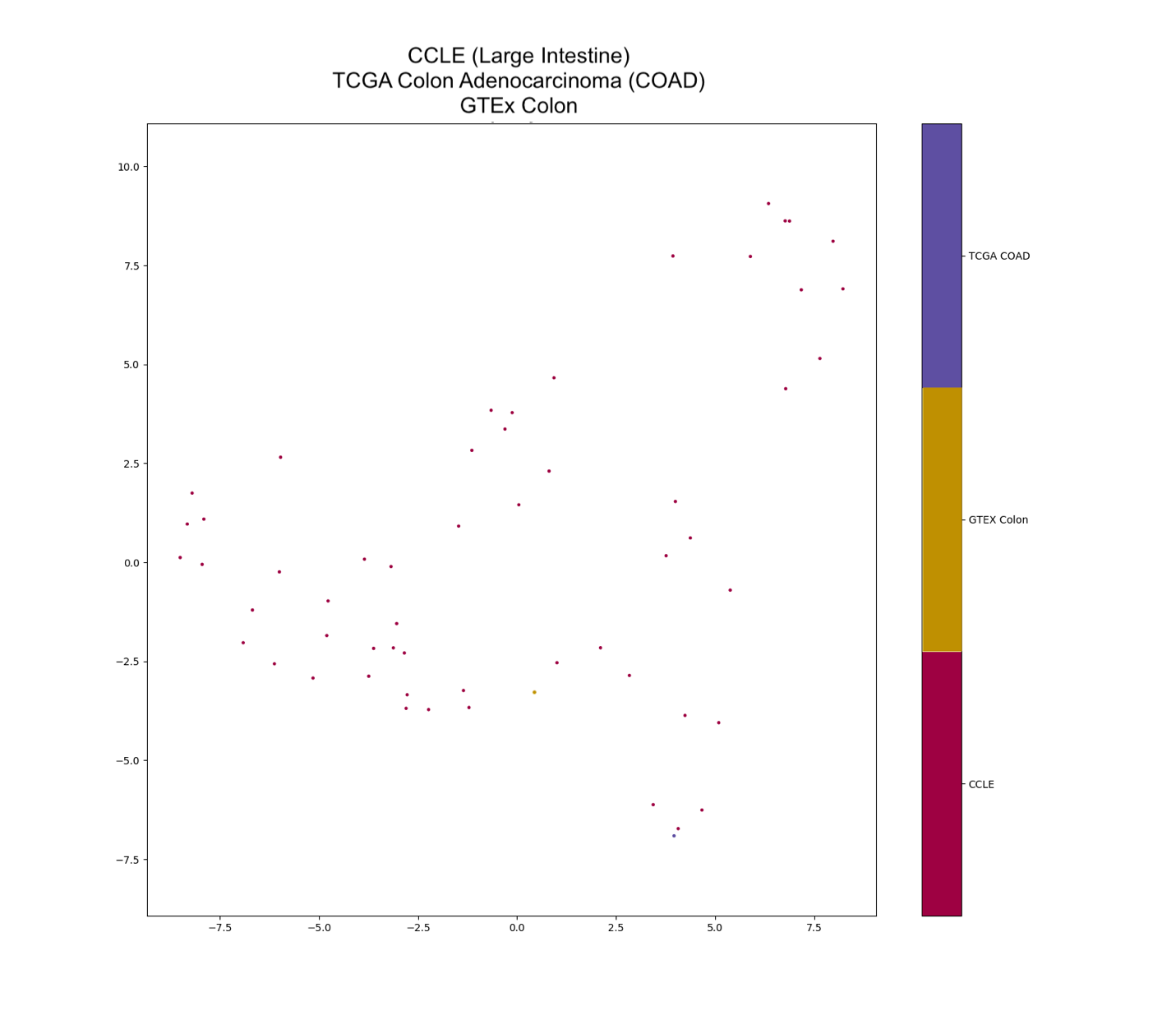

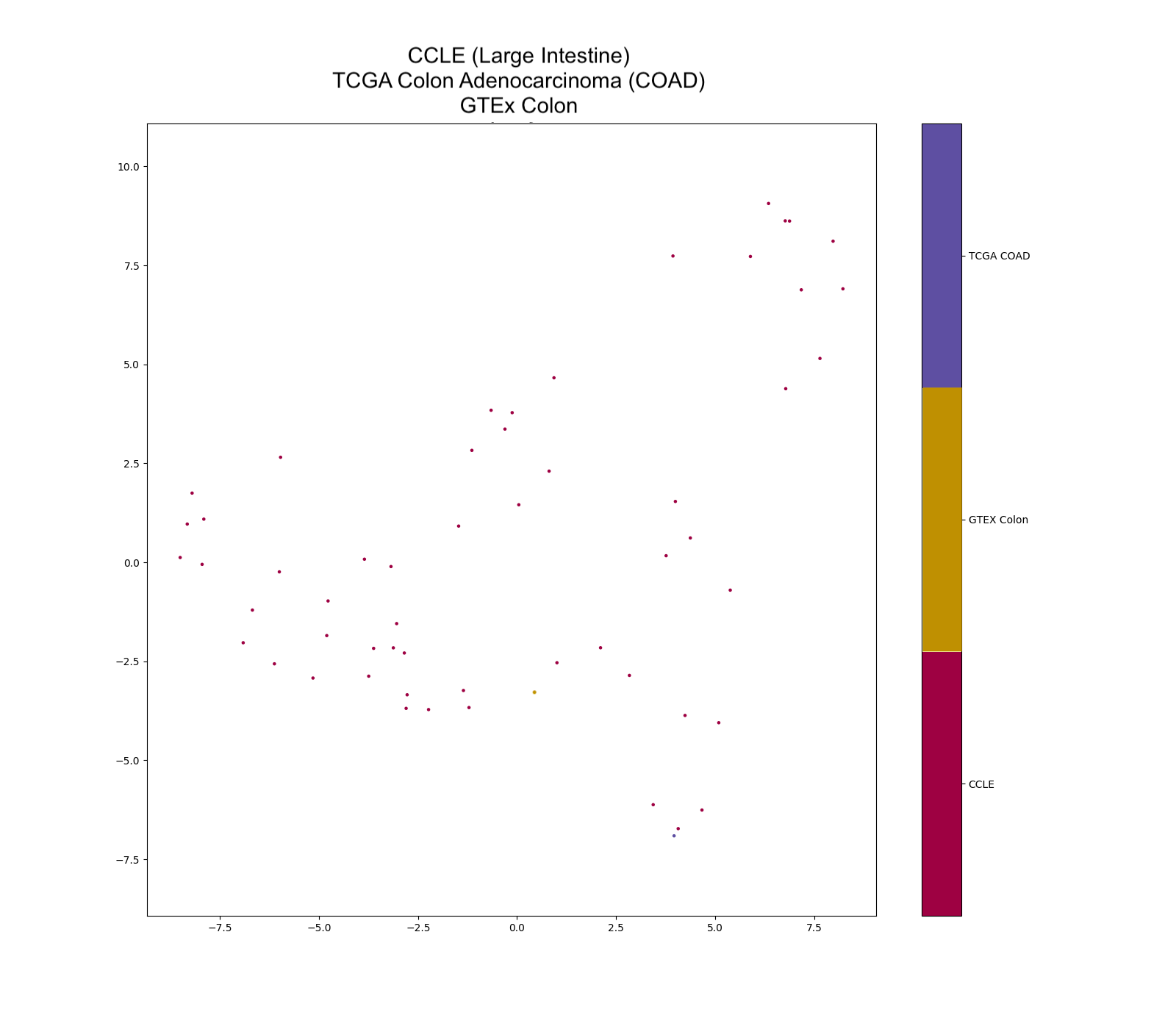

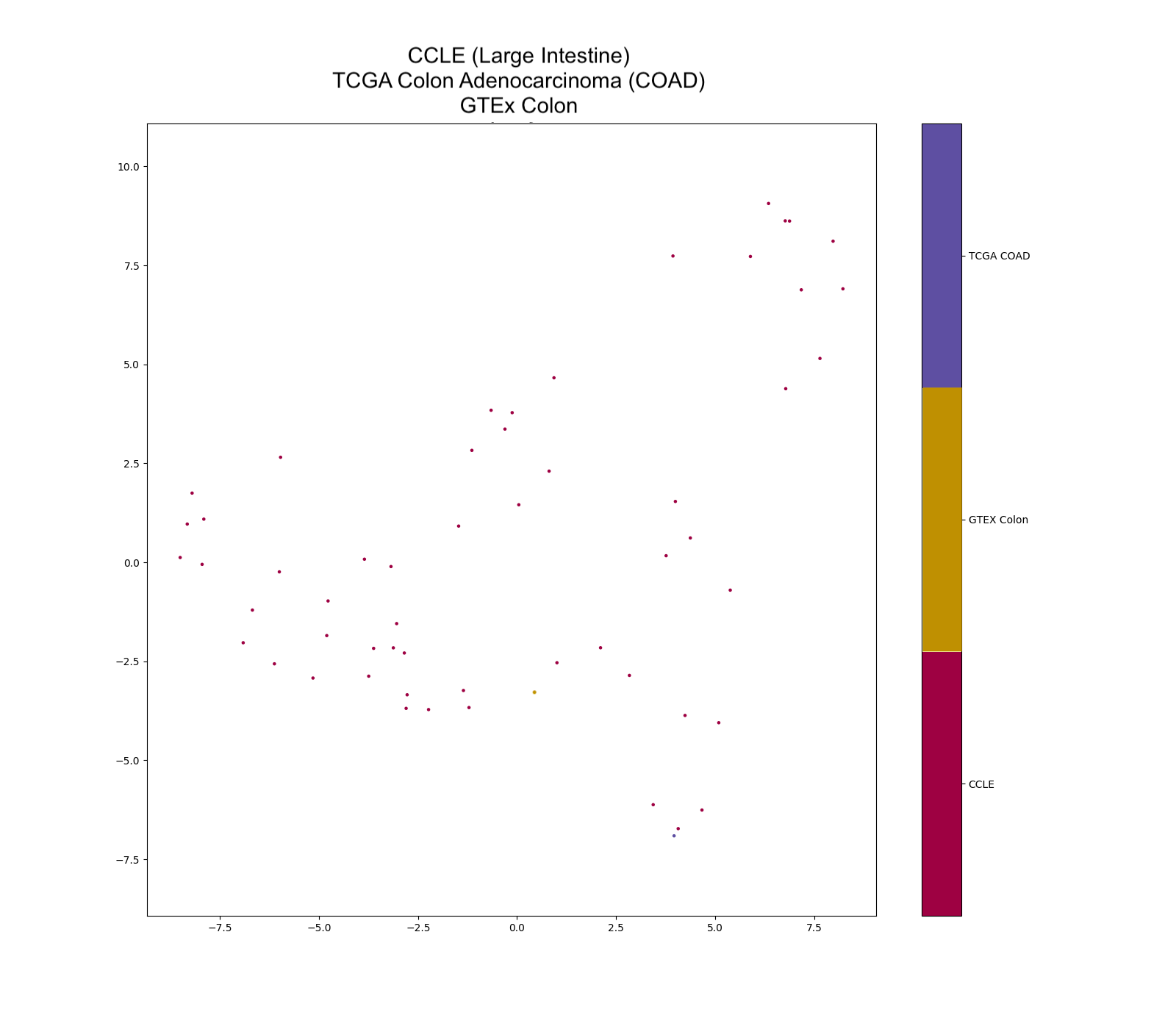


CL11^WT^

CW2^WT^

SNU1197^WT^

LS1034^WT^

**Figure S16. UMAP projection of large intestine CCLE cell lines, TCGA colon adenocarcinoma (COAD) and GTEx colon using TERT isoform expression.** Cell line CL11 was closest to average isoform percentage from TCGA COAD, accompanied by cell lines CW2 and SNU1197. Cell line LS1034 was closest to average isoform percentage from GTEx colon. Cell line TERT promoter status taken from *Ghandi et al., 2019.* Superscript “P” indicated TERT promoter mutation, superscript “WT” indicates wild-type TERT promoter and no superscript indicates no data available. Dashed-line box indicates a zoomed in region of interest with text labels of cell lines. Parameters: Manhattan distance, 8 neighbours and 4 components.


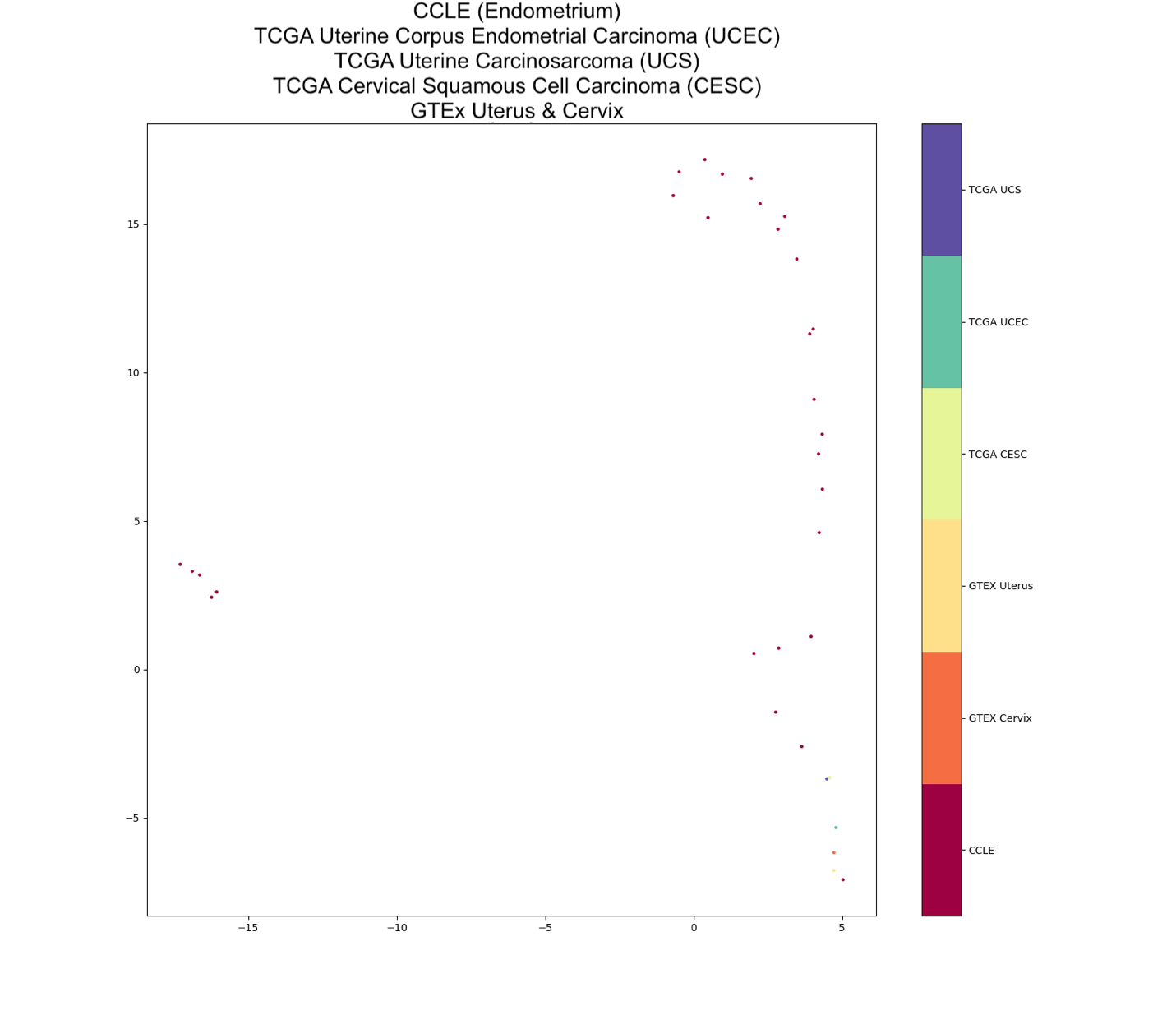

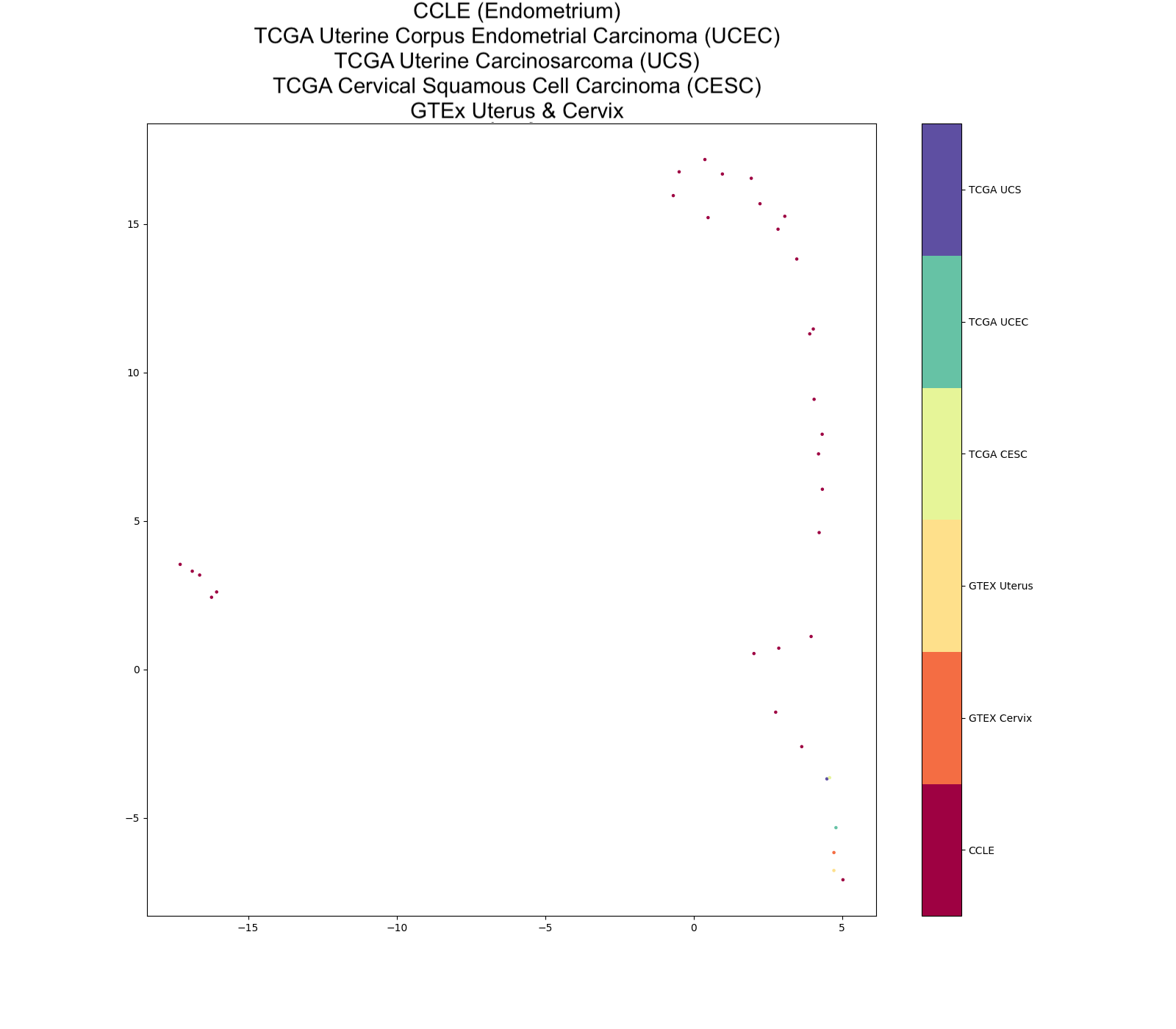


JHUEM7

JHUEM2

**Figure S17. UMAP projection of endometrium CCLE cell lines, TCGA uterine corpus endometrial carcinoma (UCEC), uterine carcinosarcoma (UCS) and cervical squamous cell carcinoma (CESC), and GTEx uterus and cervix using TERT isoform expression.** Cell line JHUEM7 was closest to average isoform percentage from GTEx tissues. Cell line JHUEM2 was closest to average isoform percentage from TCGA tissues. TCGA UCEC was also close to JHUEM7. Cell line TERT promoter status taken from *Ghandi et al., 2019.* Superscript “P” indicated TERT promoter mutation, superscript “WT” indicates wild-type TERT promoter and no superscript indicates no data available. Dashed-line box indicates a zoomed in region of interest with text labels of cell lines. Parameters: Manhattan distance, 4 neighbours and 4 components.


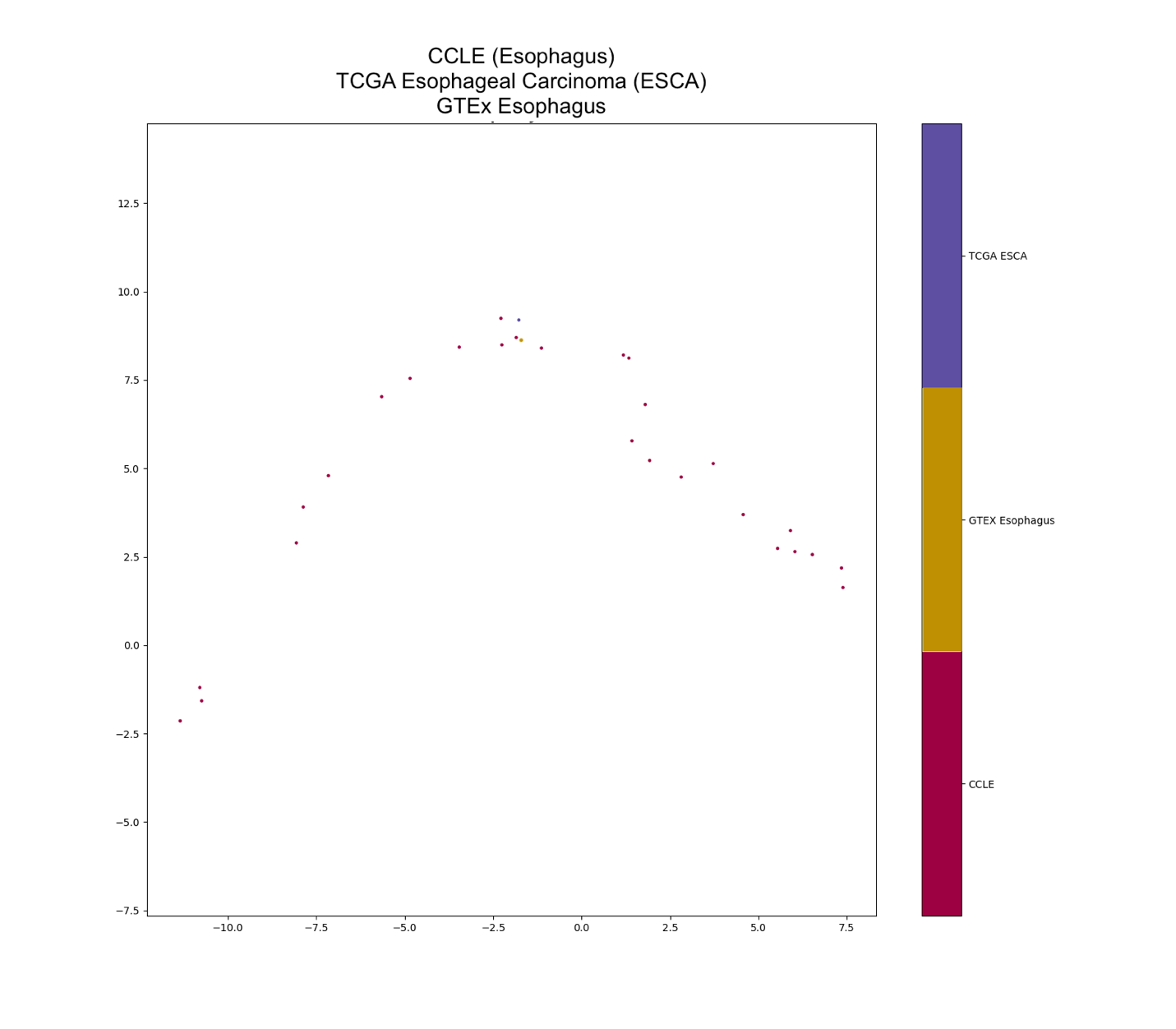

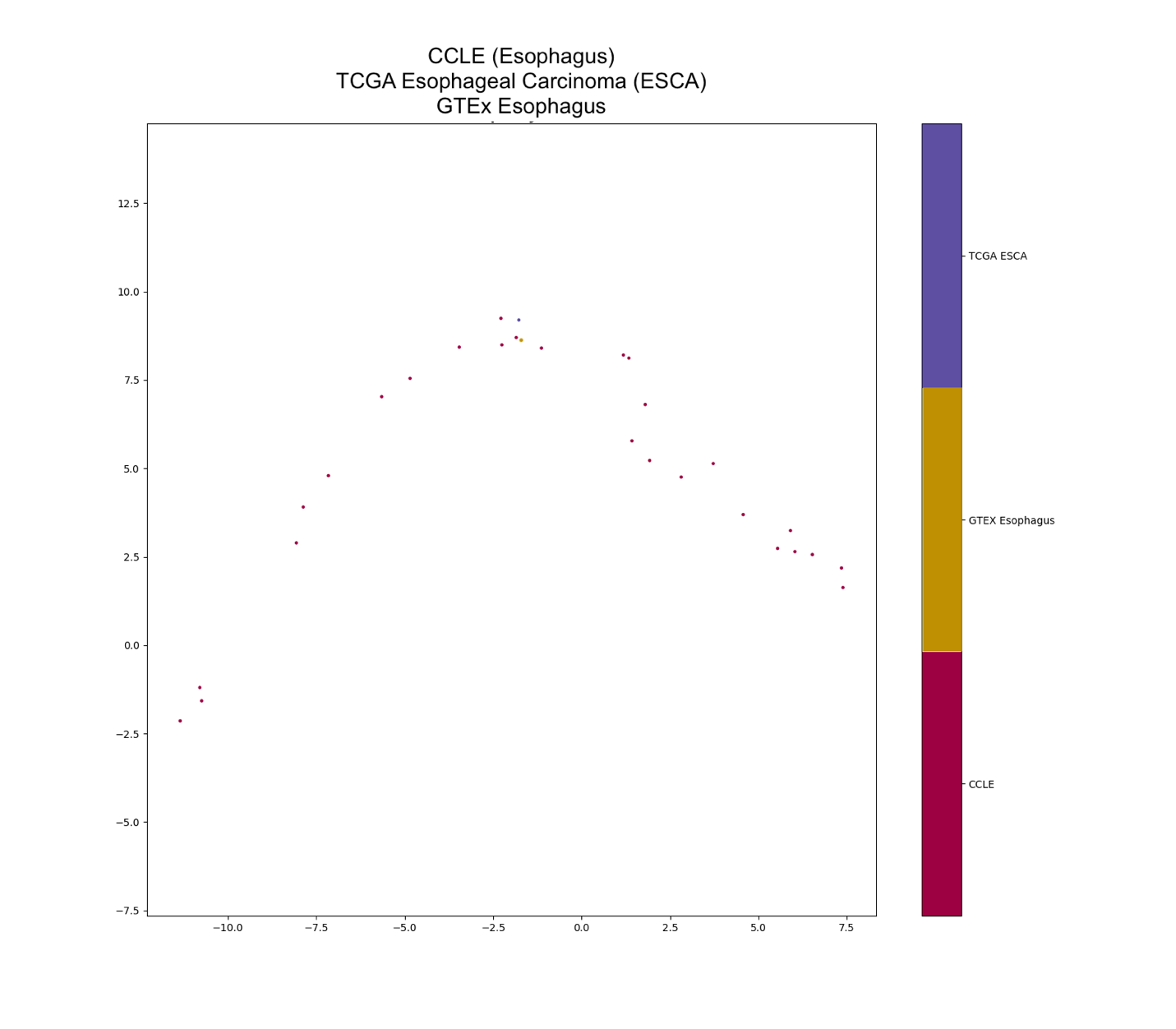


TE11^WT^

KYSE510

COLO680N

KYSE410^P^

**Figure S18. UMAP projection of esophagus CCLE cell lines, TCGA esophageal carcinoma (ESCA) and GTEx esophagus using TERT isoform expression.** Cell lines TE11, KYSE510, KYSE410 and COLO680N were closest to average isoform percentage from TCGA ESCA and GTEx esophagus. Cell line TERT promoter status taken from *Ghandi et al., 2019.* Superscript “P” indicated TERT promoter mutation, superscript “WT” indicates wild-type TERT promoter and no superscript indicates no data available. Dashed-line box indicates a zoomed in region of interest with text labels of cell lines. Parameters: Manhattan distance, 4 neighbours and 4 components.


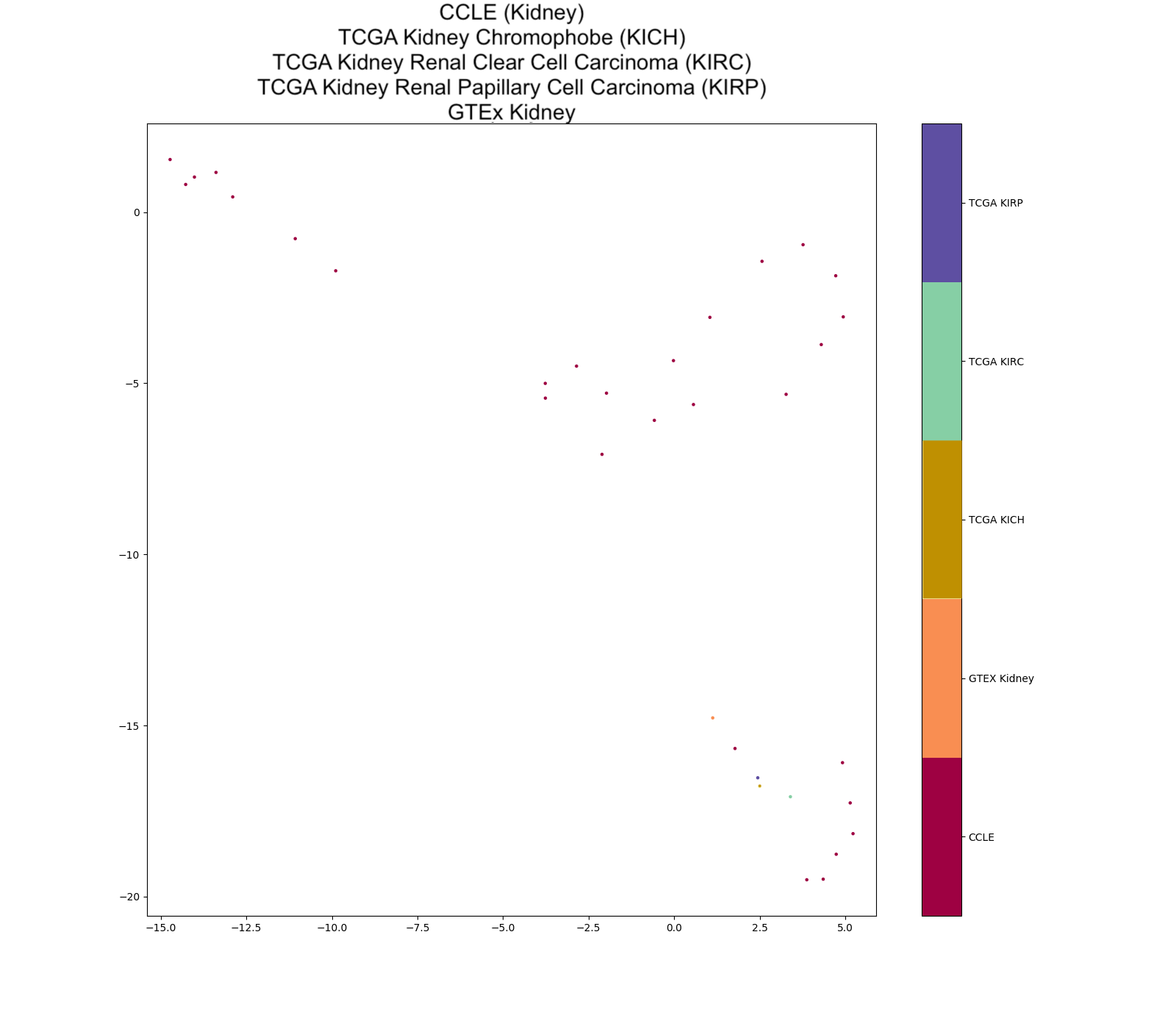

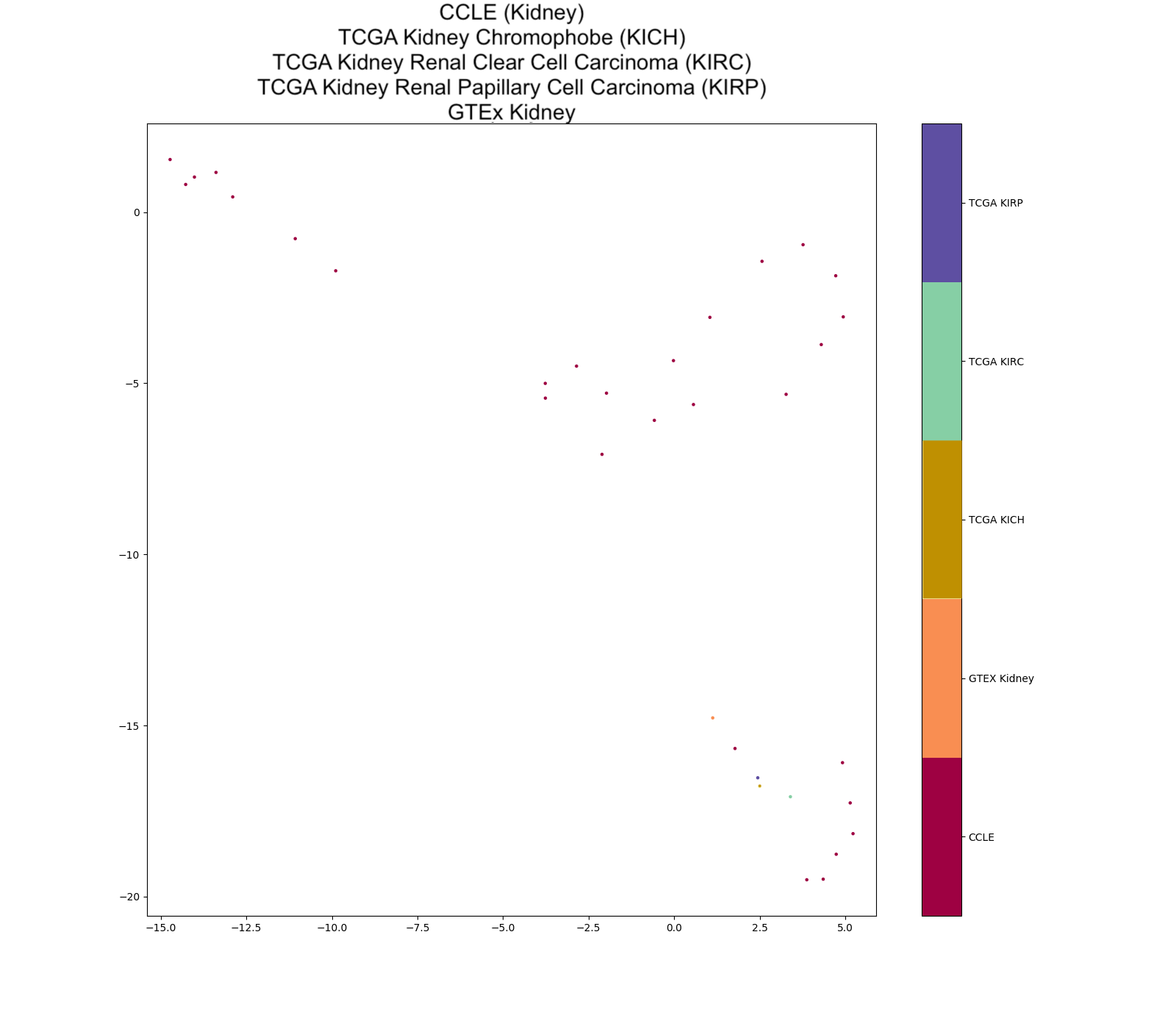


CAKI1^WT^

**Figure S19. UMAP projection of kidney CCLE cell lines, TCGA kidney chromophobe (KICH), kidney renal clear cell carcinoma (KIRC), kidney renal papillary cell carcinoma (KIRP) and GTEx kidney using TERT isoform expression.** Cell line CAKI1was closest to average isoform percentage from tumour and normal kidney tissues. Cell line TERT promoter status taken from *Ghandi et al., 2019.* Superscript “P” indicated TERT promoter mutation, superscript “WT” indicates wild-type TERT promoter and no superscript indicates no data available. Dashed-line box indicates a zoomed in region of interest with text labels of cell lines. Parameters: Manhattan distance, 4 neighbours and 4 components.


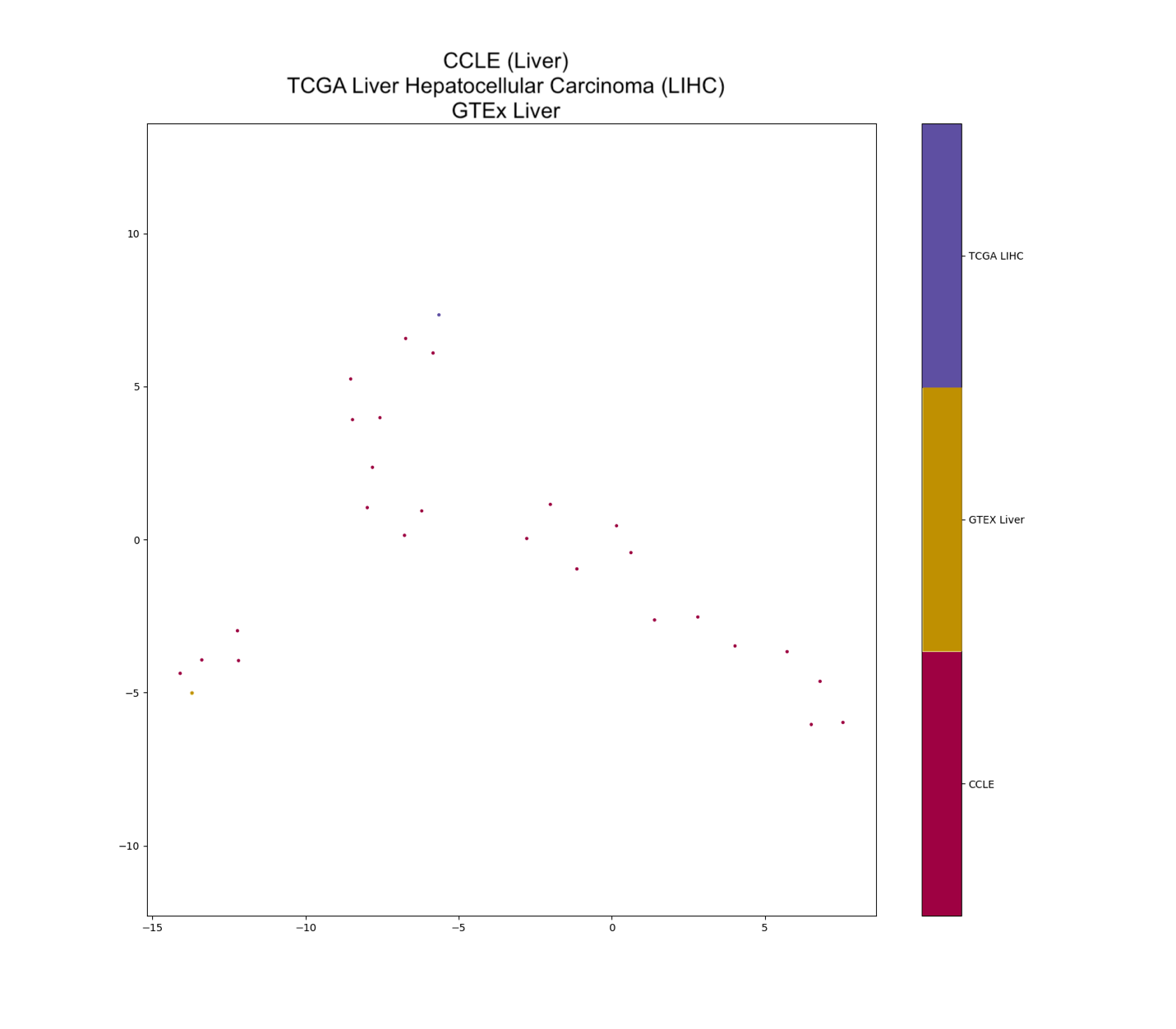

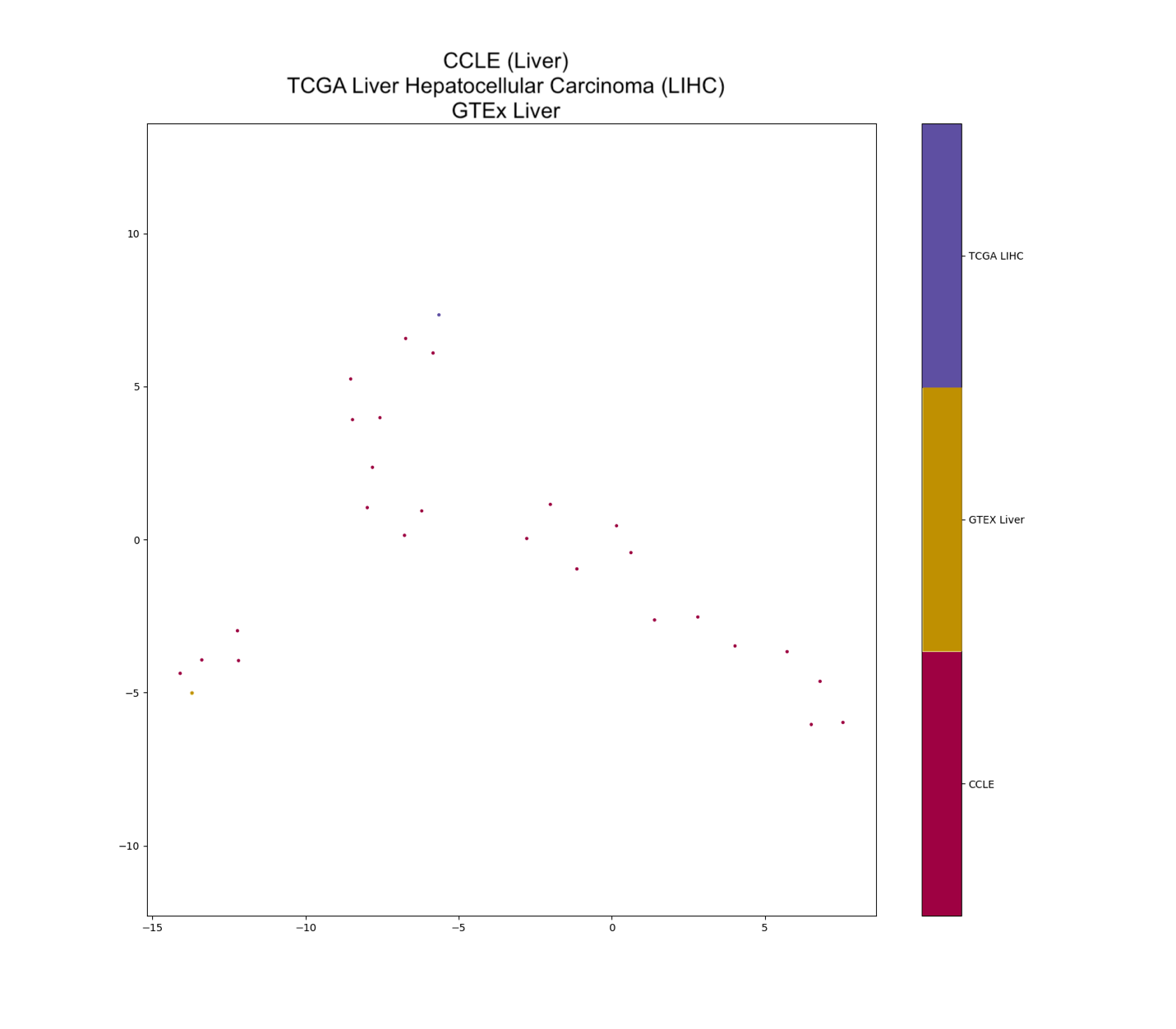

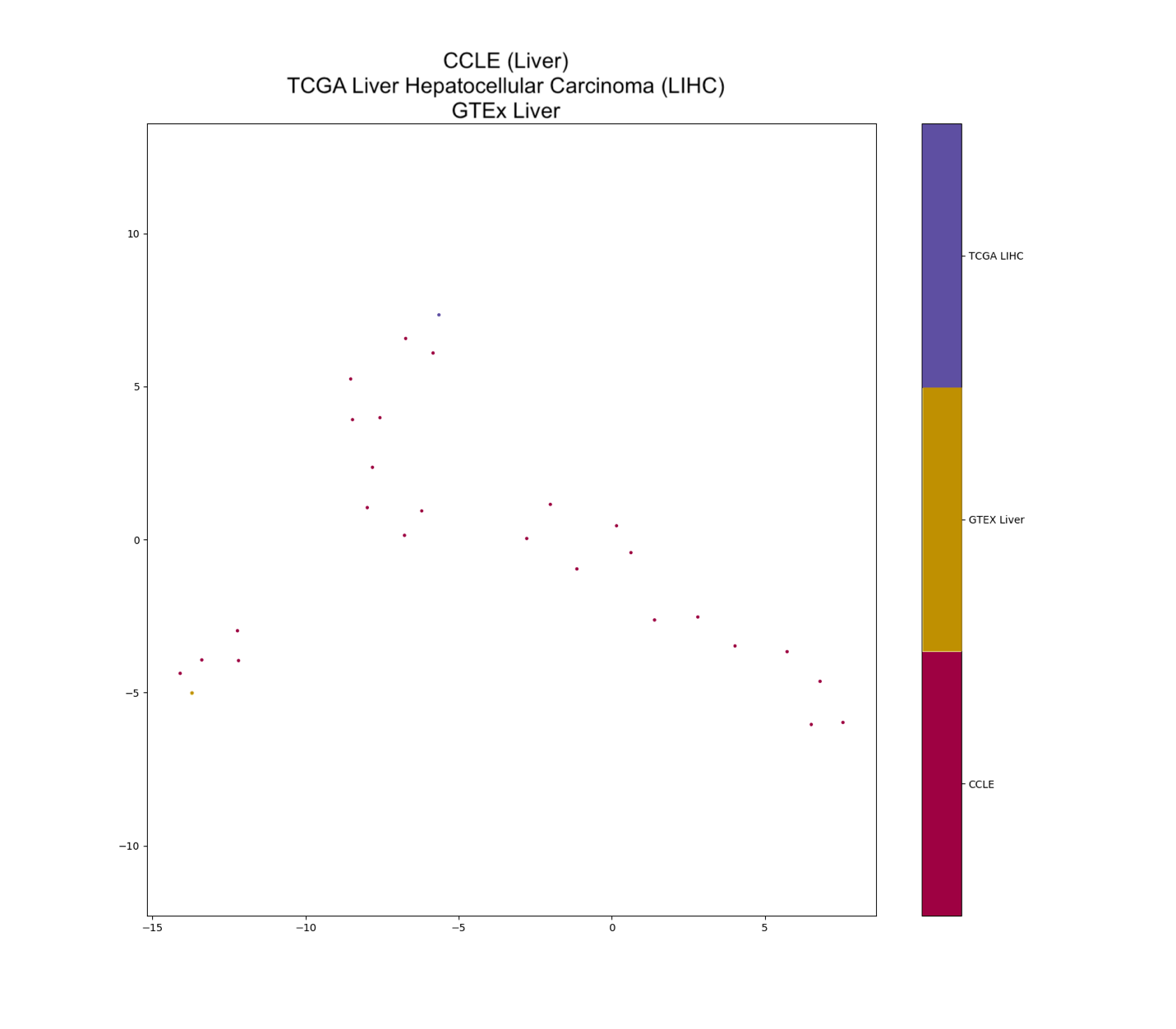


HEPG2^P^

LI7

JHH1^WT^

SNU878

**Figure S20. UMAP projection of liver CCLE cell lines, TCGA liver hepatocellular carcinoma (LIHC) and GTEx liver using TERT isoform expression.** Cell lines HEPG2 and LI7 were closest to average isoform percentage from TCGA LIHC. Cell lines SNU878 and JHH1 were closest to average isoform percentage from GTEx liver. Cell line TERT promoter status taken from *Ghandi et al., 2019.* Superscript “P” indicated TERT promoter mutation, superscript “WT” indicates wild-type TERT promoter and no superscript indicates no data available. Dashed-line box indicates a zoomed in region of interest with text labels of cell lines. Parameters: Manhattan distance, 4 neighbours and 4 components.


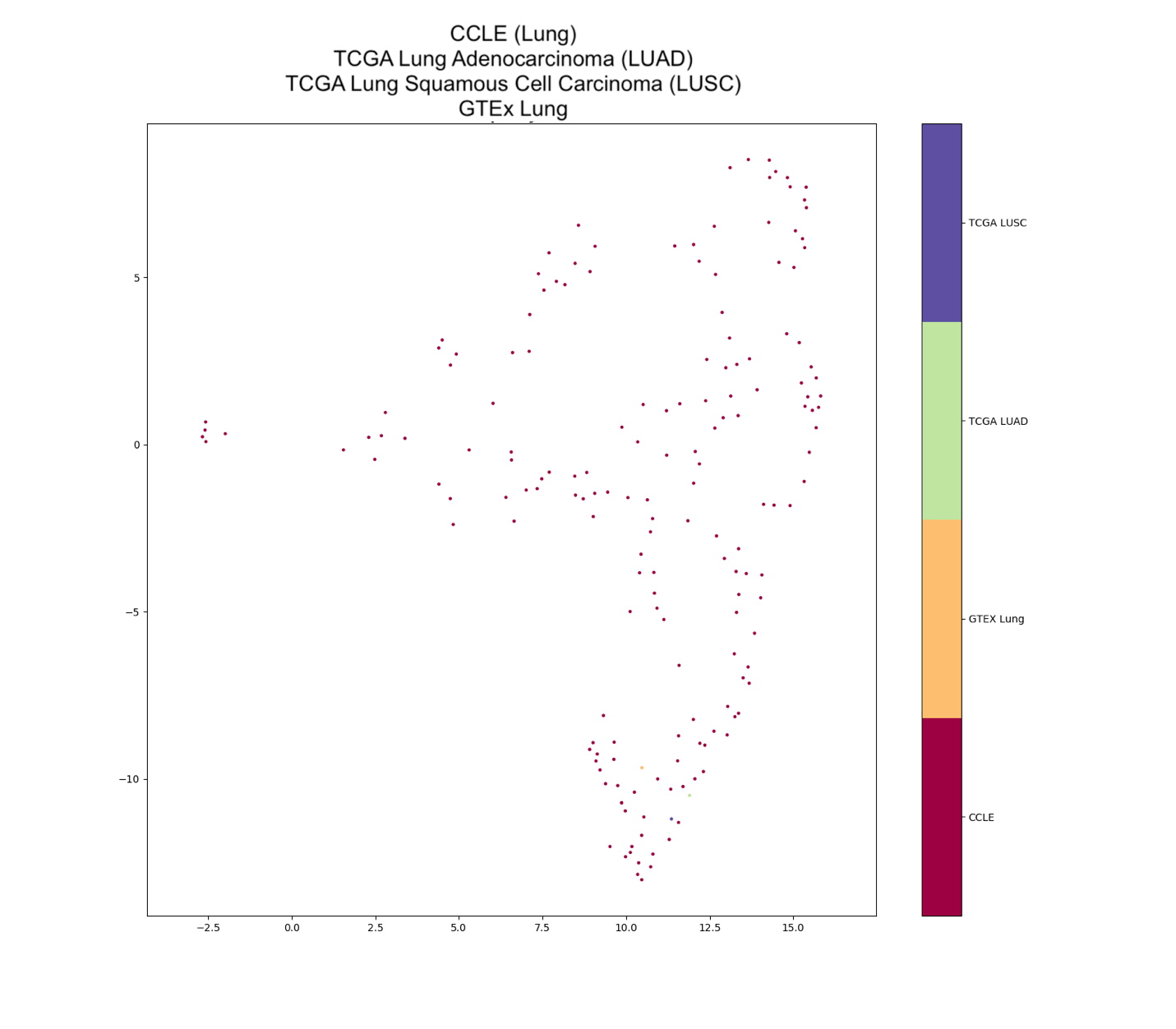

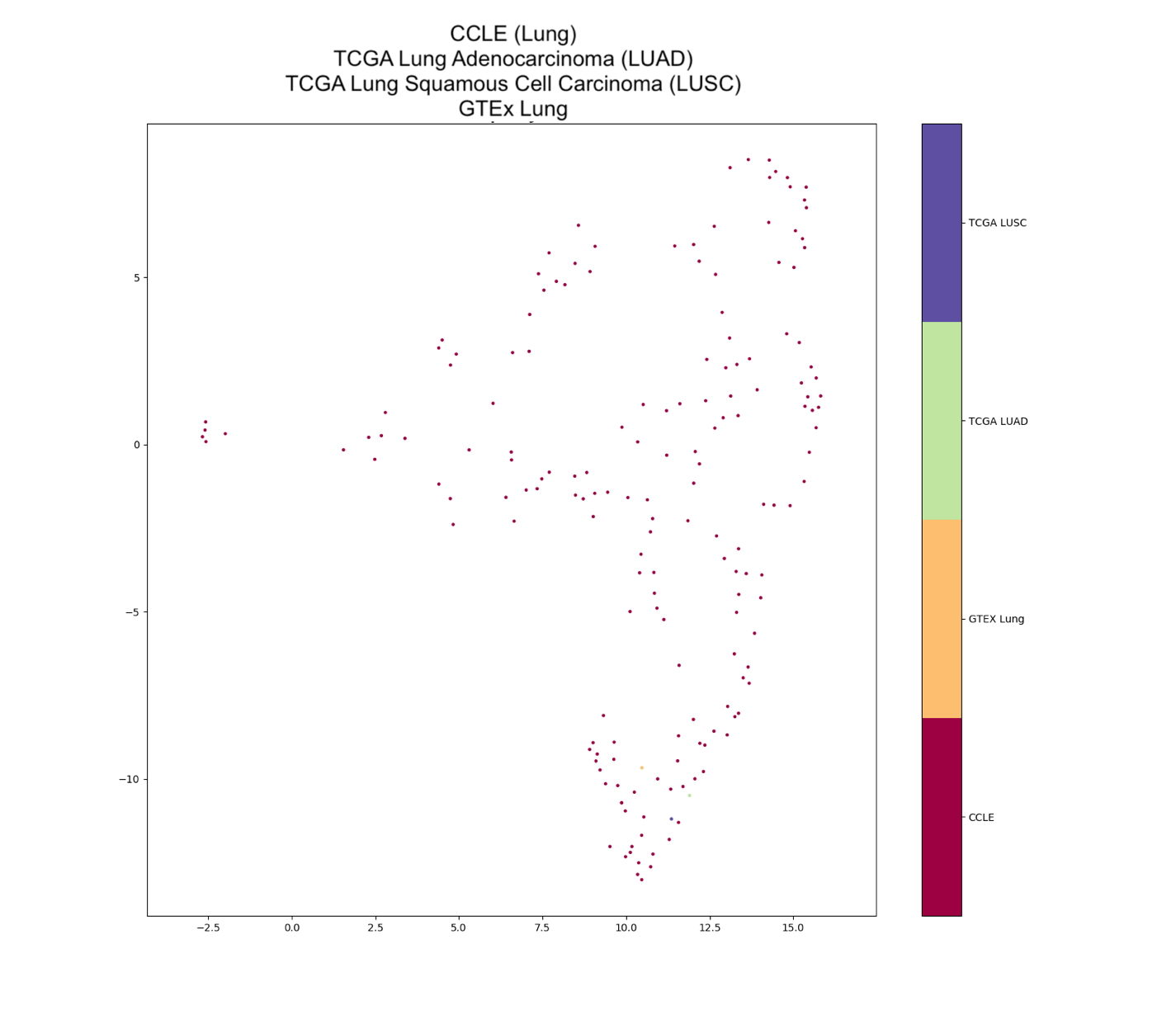


NCIH2228^WT^

NCIH1755^WT^

CORL47^WT^

NCIH596^WT^

NCIH524^WT^

NCIH2030^WT^

NCIH1836^WT^

NCIH727^WT^

NCIH2066^WT^

DMS153

MORCPR

**Figure S21. UMAP projection of lung CCLE cell lines, TCGA lung adenocarcinoma (LUAD), TCGA lung squamous cell carcinoma (LUSC) and GTEx lung using TERT isoform expression.** Cell lines NCIH596, NCIH2228, NCIH1755 and CORL47 were closest to average isoform percentage from TCGA LUSC. Cell lines NCIH2030, DMS152, NCIH727 and MORCPR were closest to average isoform percentage from TCGA LUSC. Cell lines NCIH524, NCIH1836 and NCIH2066 were closest to average isoform percentage from GTEx lung. Cell line TERT promoter status taken from *Ghandi et al., 2019.* Superscript “P” indicated TERT promoter mutation, superscript “WT” indicates wild-type TERT promoter and no superscript indicates no data available. Dashed-line box indicates a zoomed in region of interest with text labels of cell lines. Parameters: Manhattan distance, 16 neighbours and 4 components.


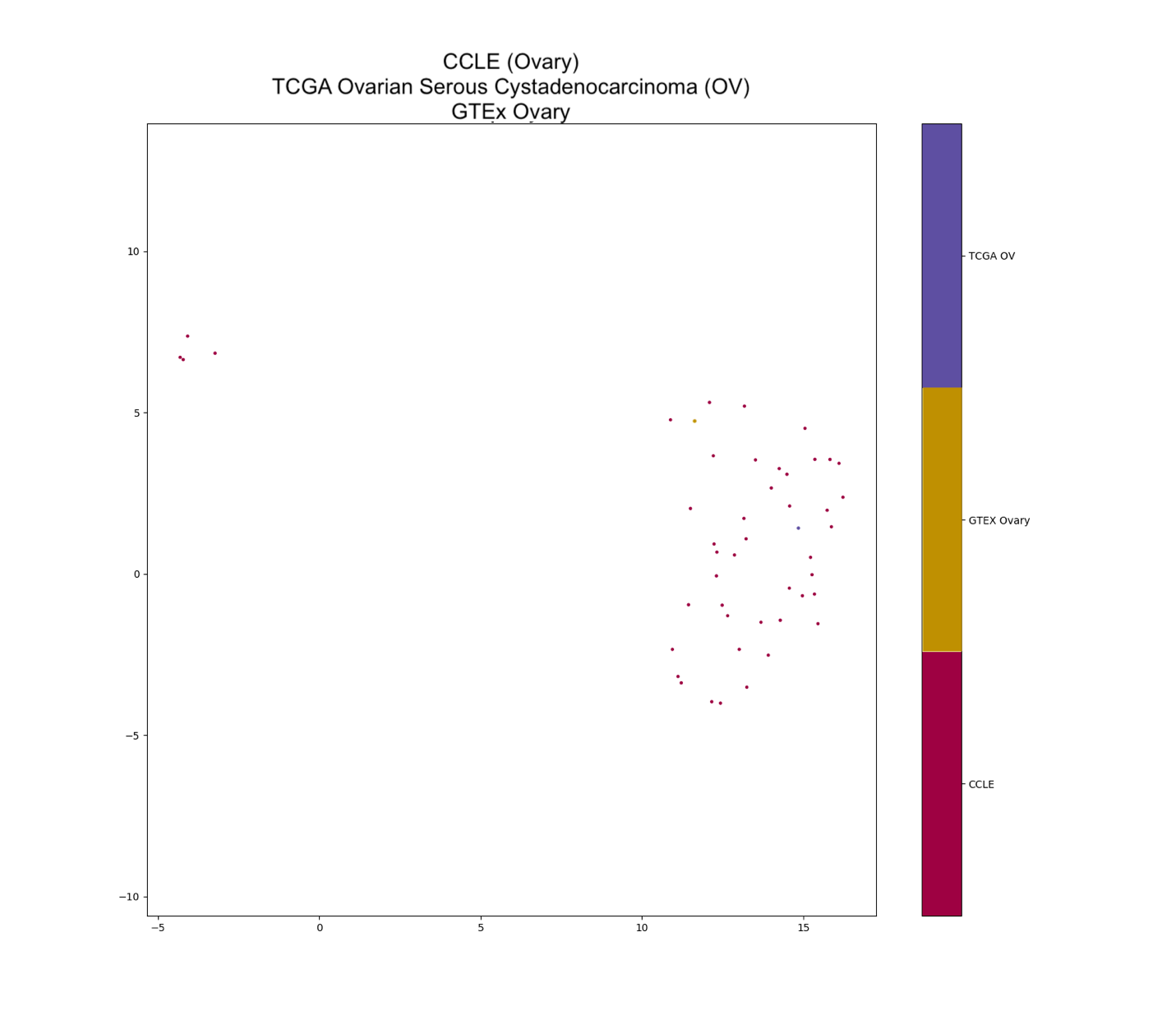

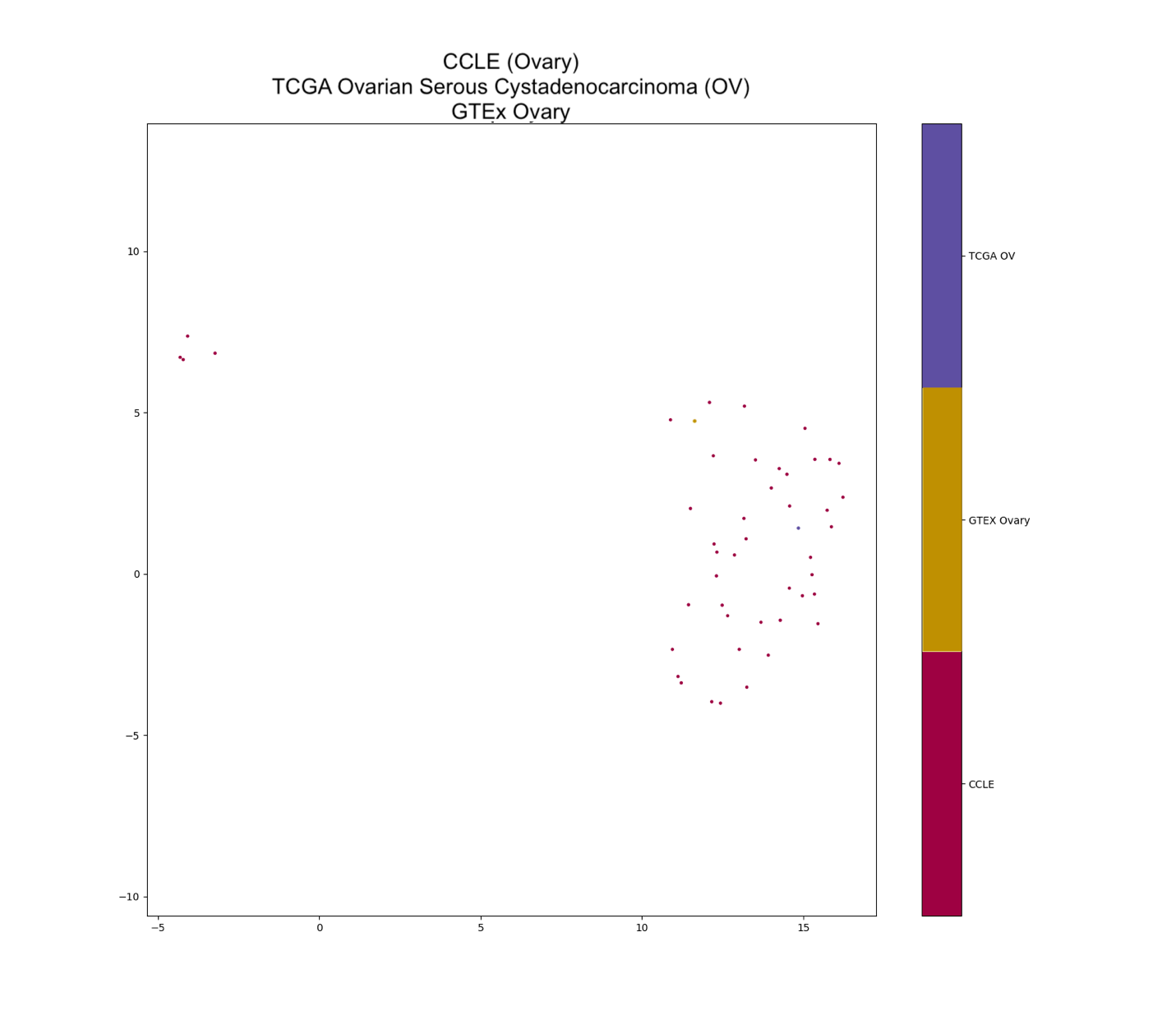

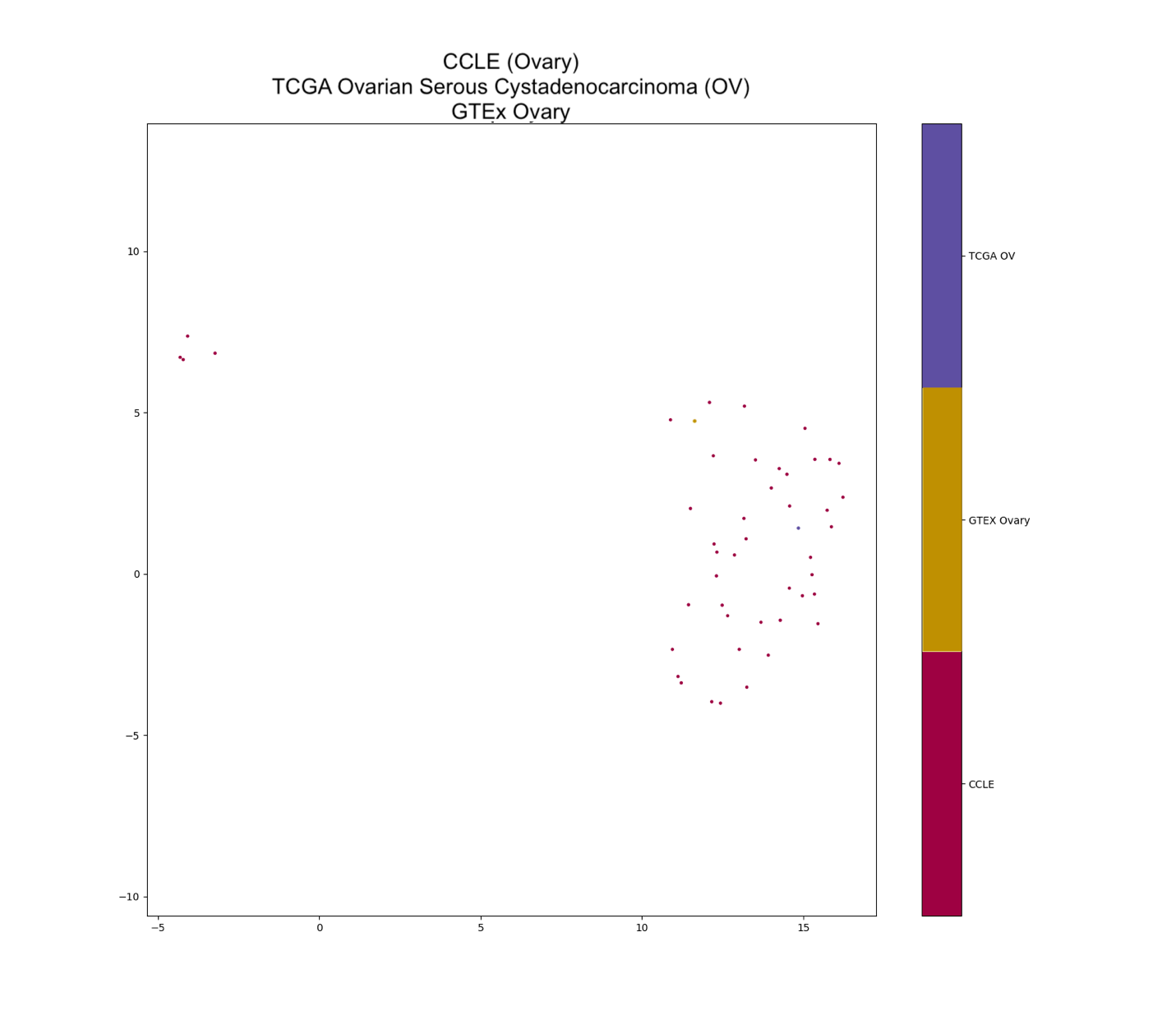


HEYA8

OV90^WT^

OVKATE

TOV112D

JHOM1

COV362^WT^

OVK18^WT^

**Figure S22. UMAP projection of ovarian CCLE cell lines, TCGA ovarian serous cystadenocarcinoma (OV) and GTEx ovary using TERT isoform expression.** Cell lines COV362, JHOM1, TOV112D and OVK18 were closest to average isoform percentage from TCGA OV. Cell lines OVKATE, OV90 and HEYA8 were closest to average isoform percentage from GTEx ovary. Cell line TERT promoter status taken from *Ghandi et al., 2019.* Superscript “P” indicated TERT promoter mutation, superscript “WT” indicates wild-type TERT promoter and no superscript indicates no data available. Dashed-line box indicates a zoomed in region of interest with text labels of cell lines. Parameters: Manhattan distance, 8 neighbours and 4 components.


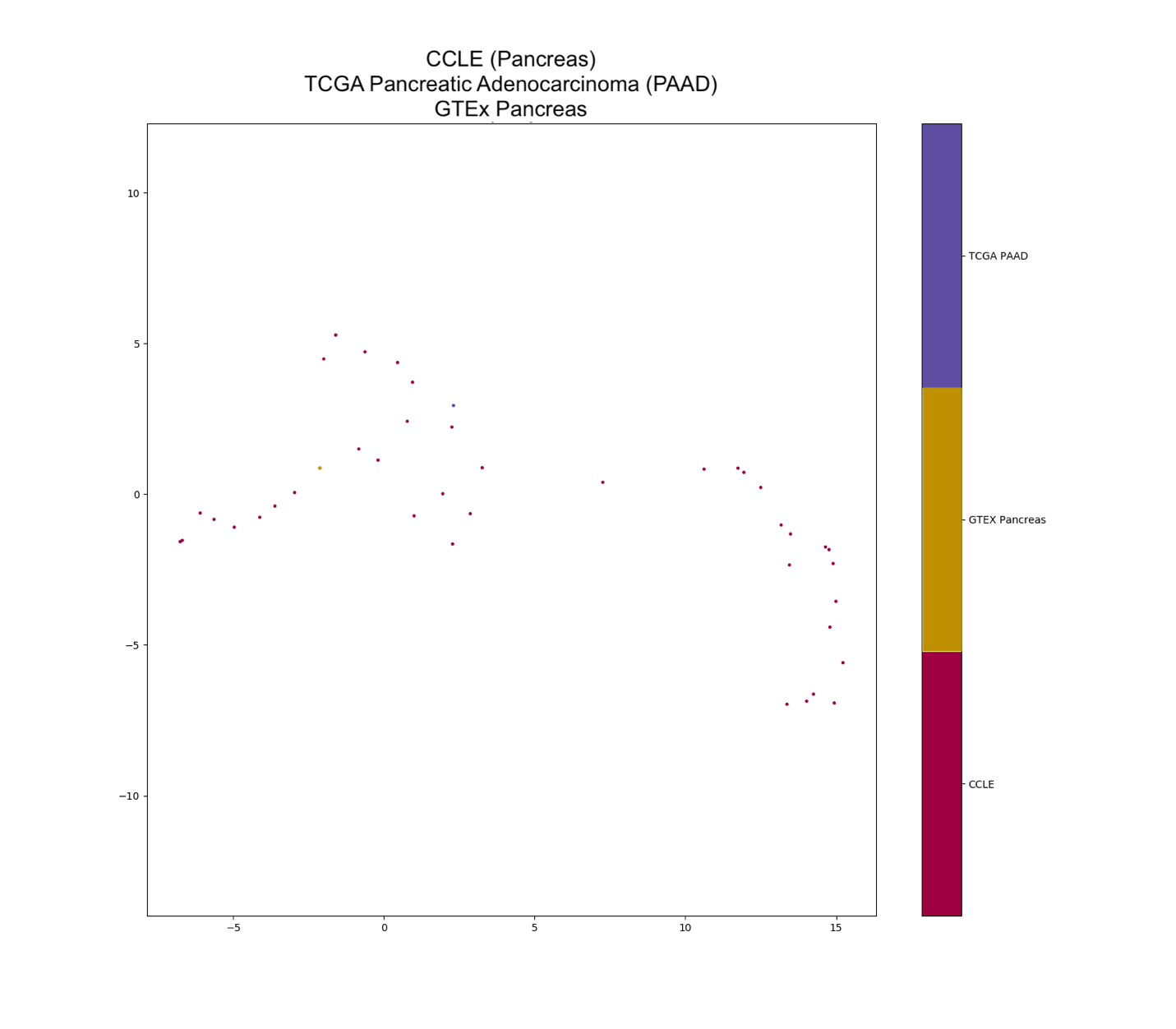

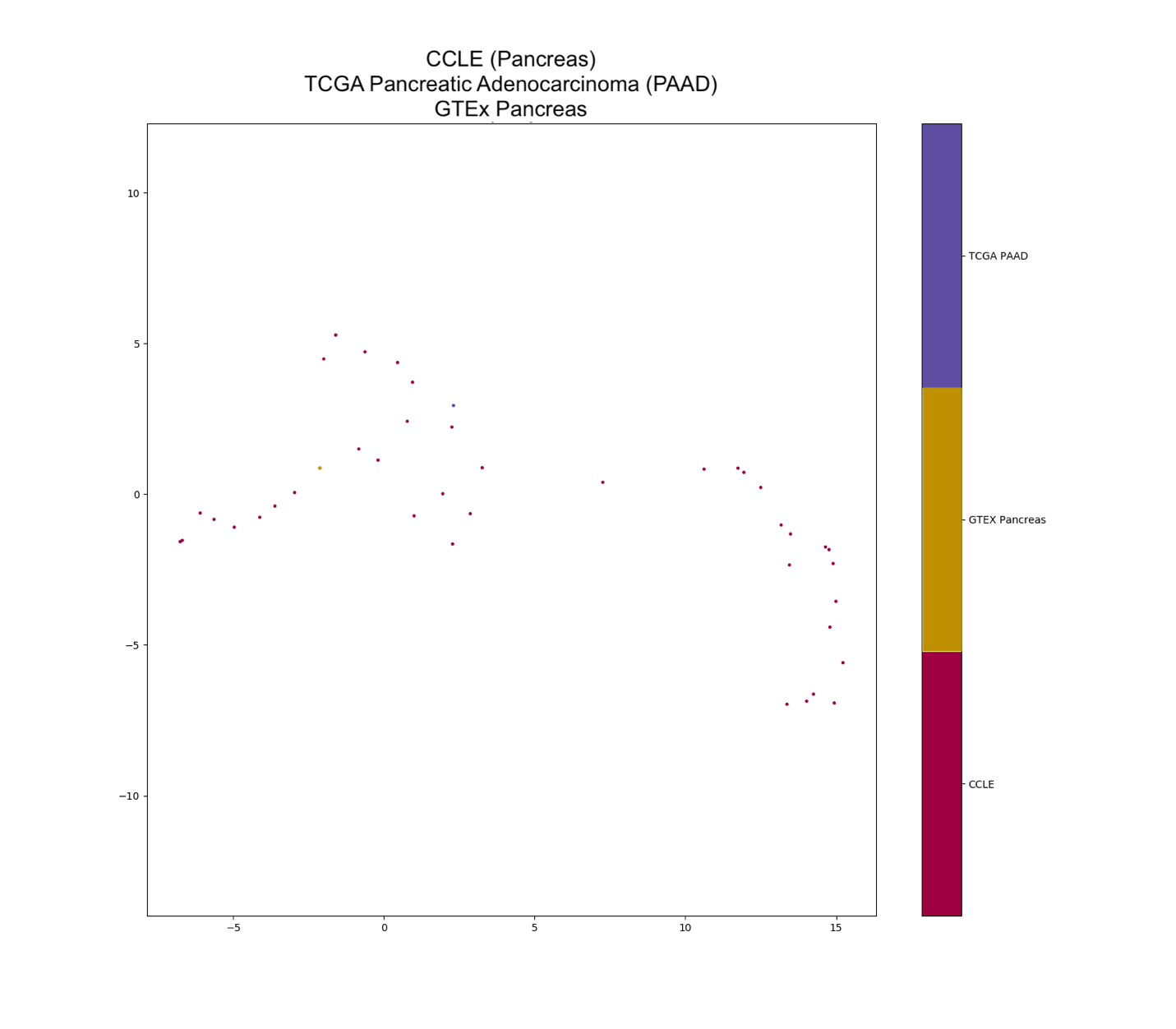

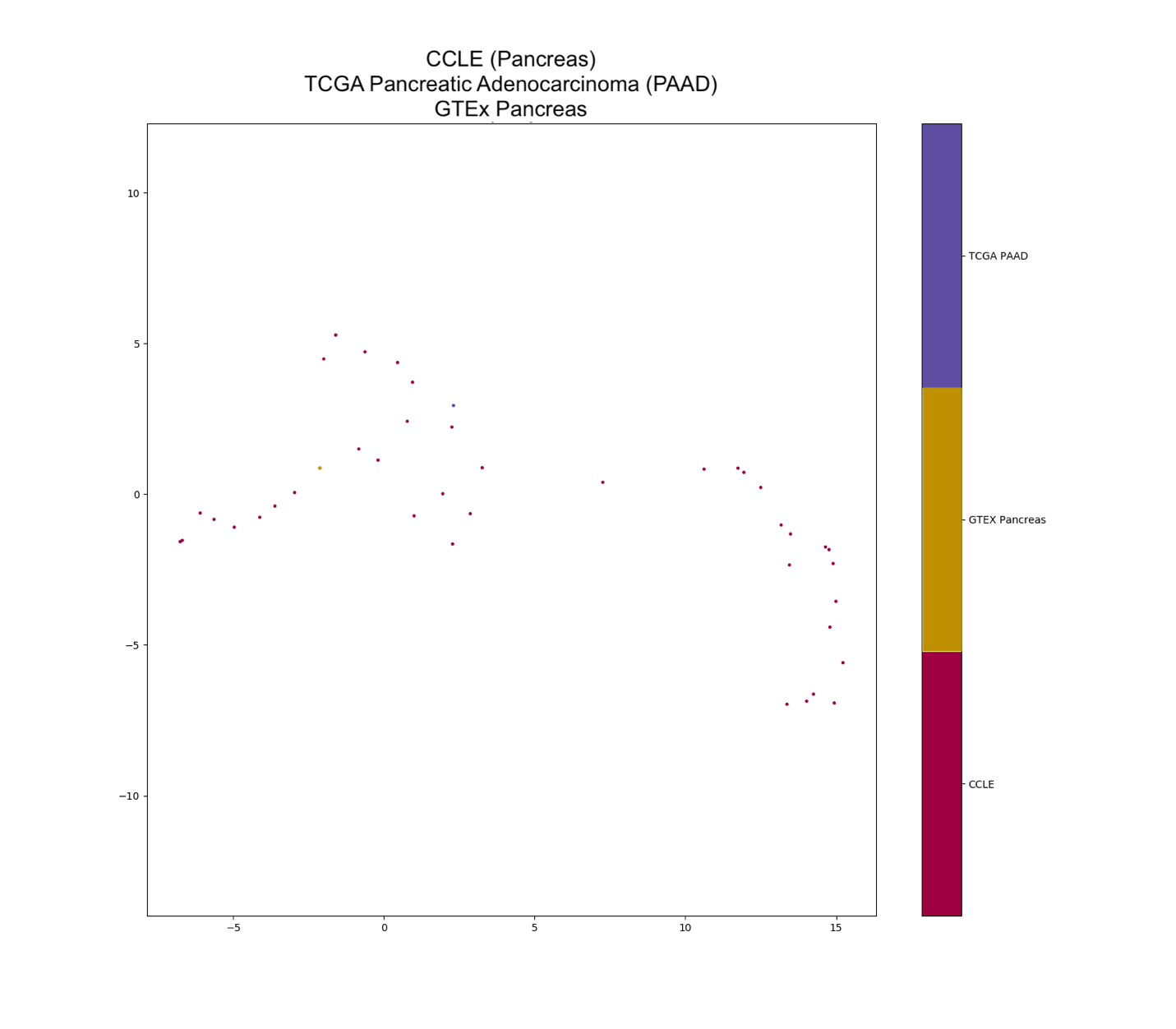


CAPAN1^WT^

MIAPACA2^WT^

PANC1005^WT^

CFPAC1

**Figure S23. UMAP projection of pancreas CCLE cell lines, TCGA pancreatic adenocarcinoma (PAAD) and GTEx pancreas using TERT isoform expression.** Cell line CAPAN1 was closest to average isoform percentage from TCGA PAAD. Cell lines PANC1005, MIAPACA2 and CFPAC1 were closest to average isoform percentage from GTEx pancreas. Cell line TERT promoter status taken from *Ghandi et al., 2019.* Superscript “P” indicated TERT promoter mutation, superscript “WT” indicates wild-type TERT promoter and no superscript indicates no data available. Dashed-line box indicates a zoomed in region of interest with text labels of cell lines. Parameters: Manhattan distance, 8 neighbours and 4 components.


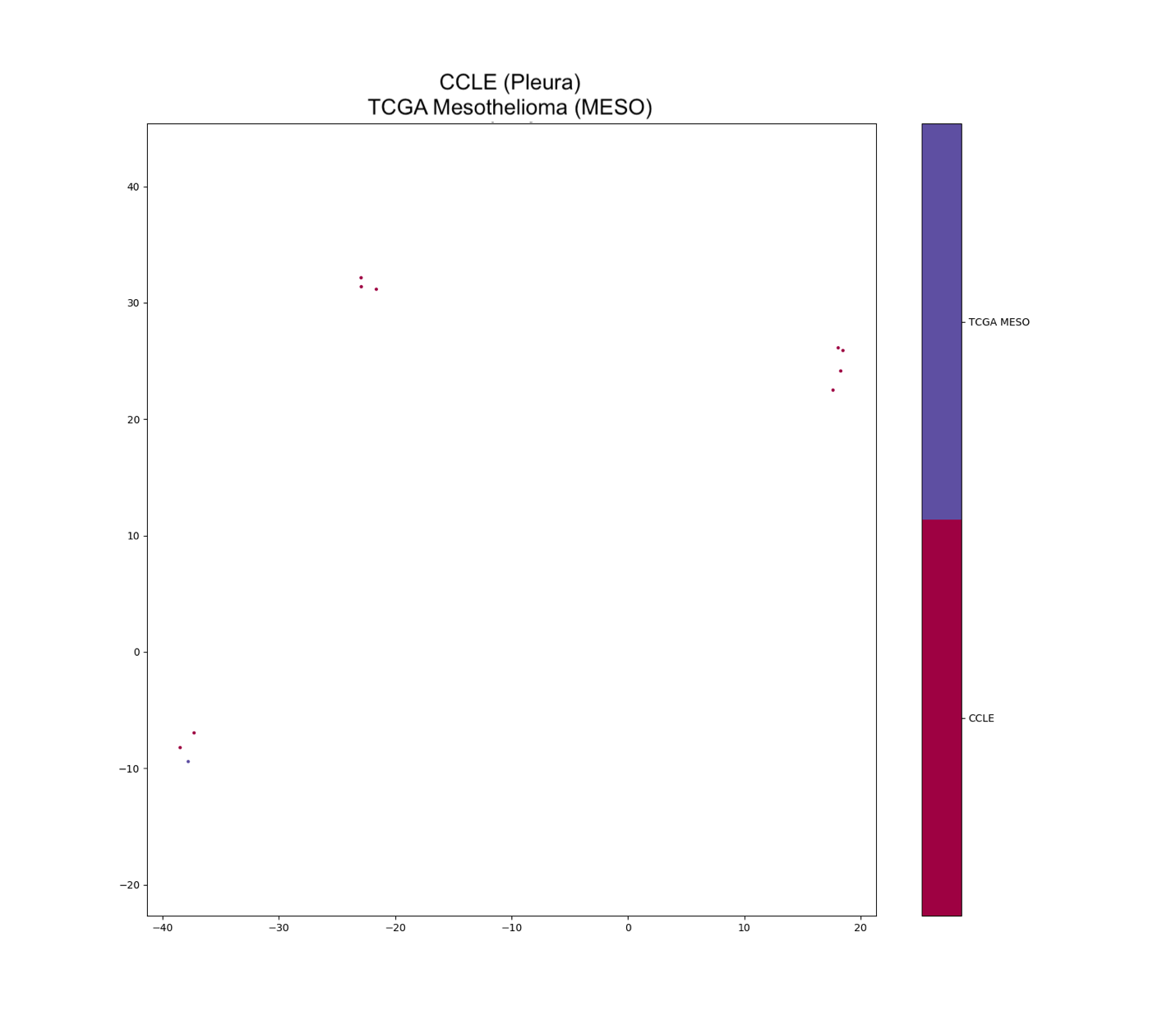

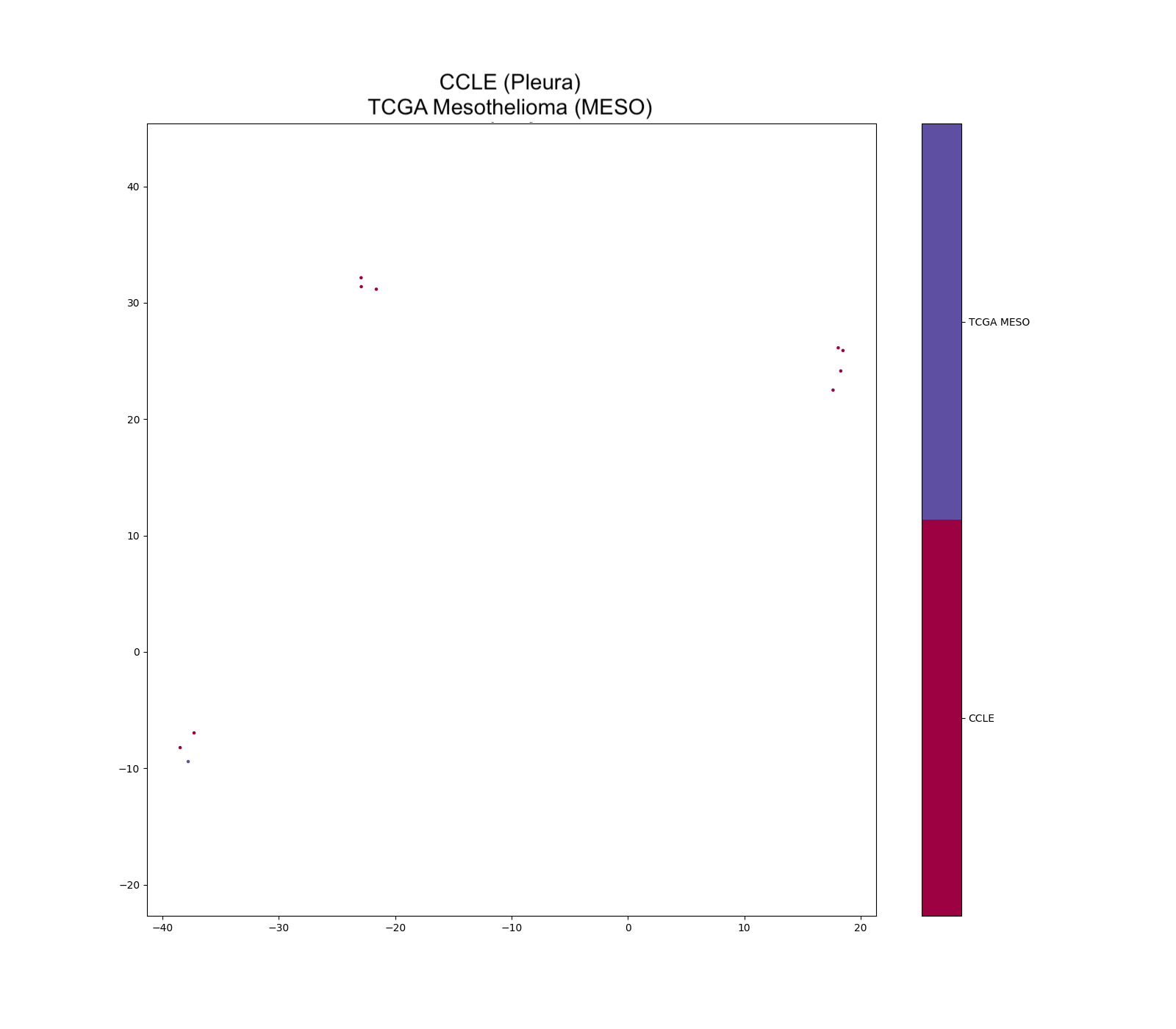


ISTMES2^WT^

NCIH2052^P^

**Figure S24. UMAP projection of pleura CCLE cell lines and TCGA mesothelioma (MESO) using TERT isoform expression.** Cell lines NCIH2052 and ISTMES2 were closest to average isoform percentage from TCGA MESO. Cell line TERT promoter status taken from *Ghandi et al., 2019.* Superscript “P” indicated TERT promoter mutation, superscript “WT” indicates wild-type TERT promoter and no superscript indicates no data available. Dashed-line box indicates a zoomed in region of interest with text labels of cell lines. Parameters: Manhattan distance, 2 neighbours and 3 components.


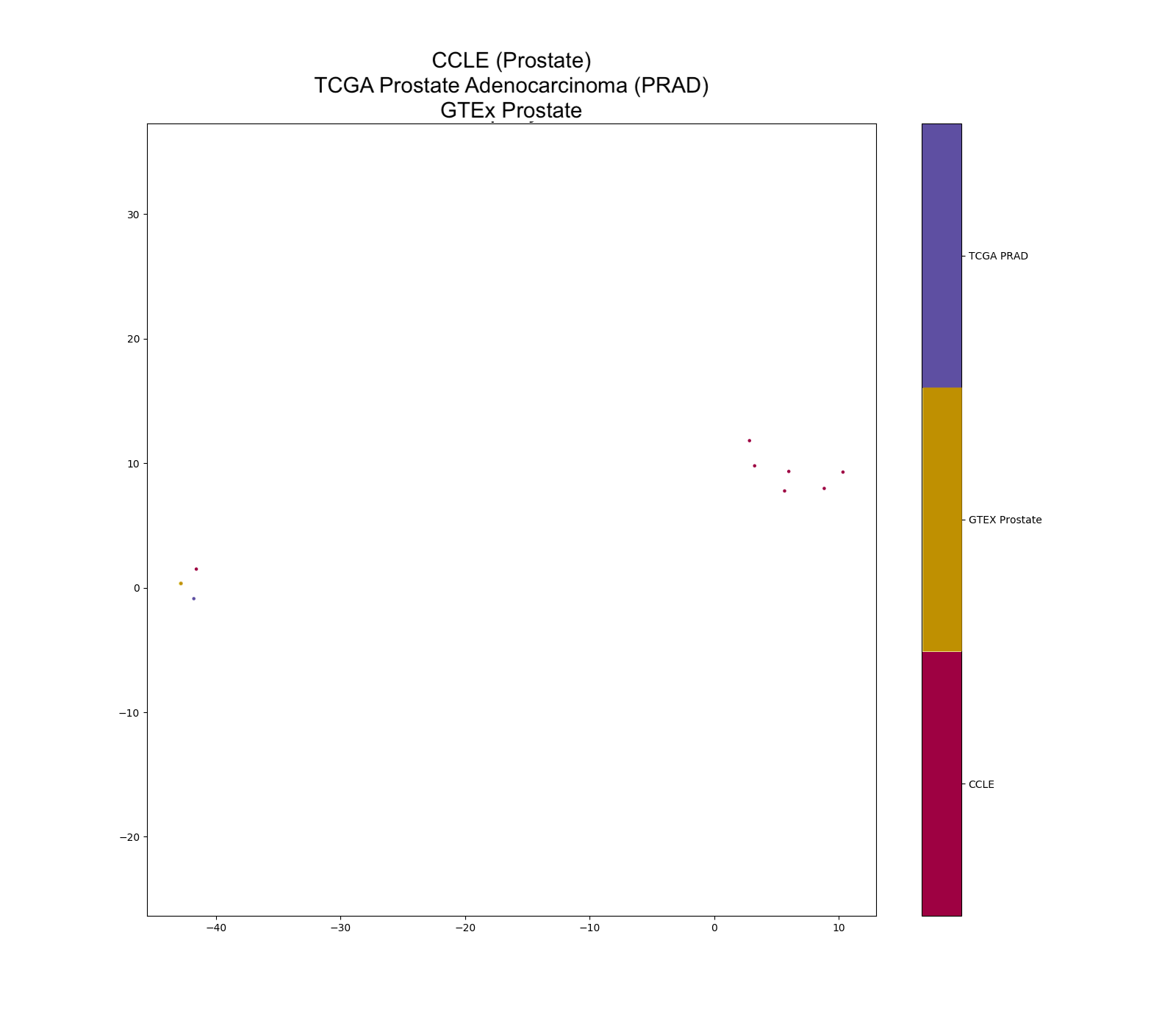

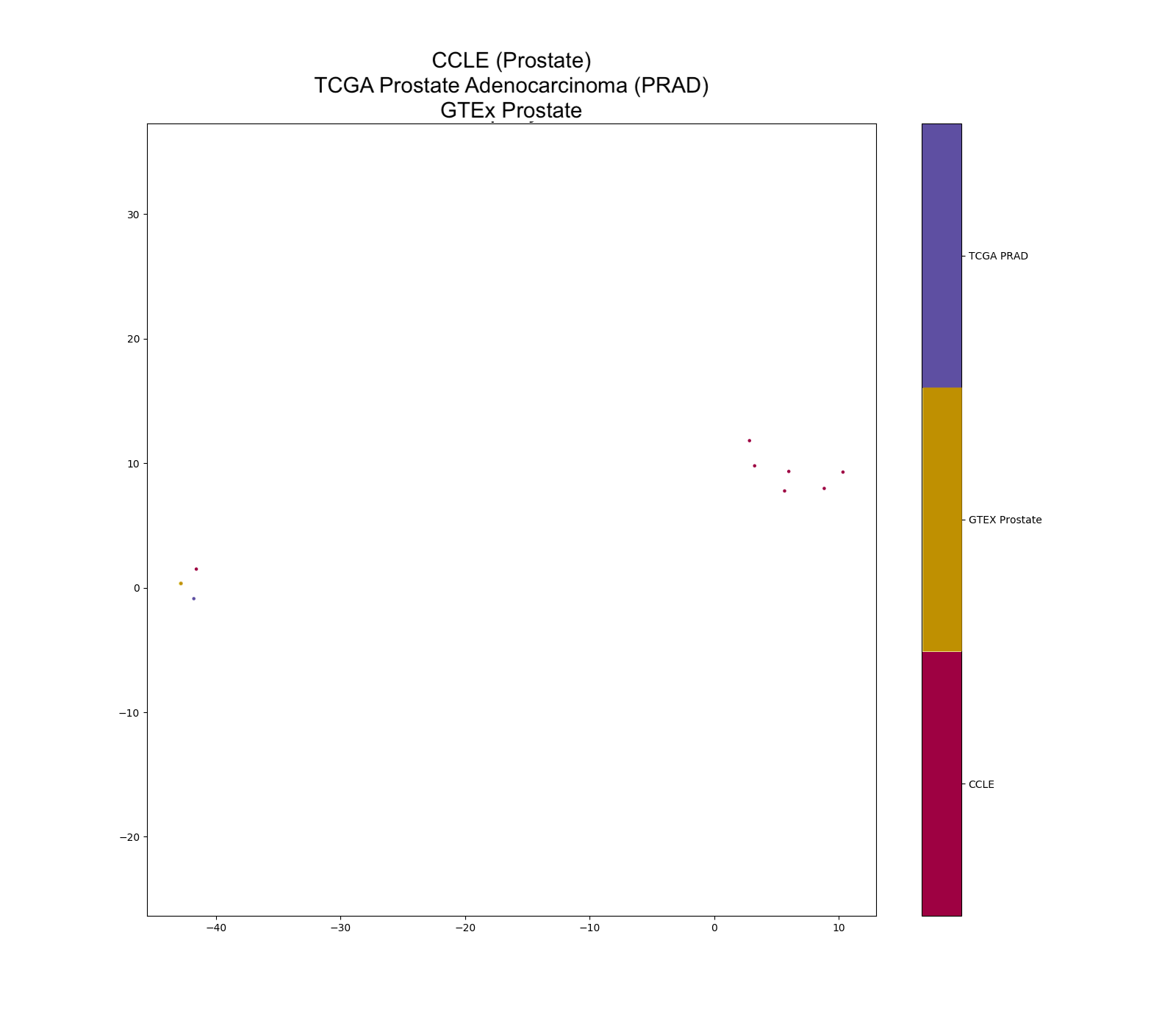


PC3^WT^

**Figure S25. UMAP projection of prostate CCLE cell lines and TCGA prostate adenocarcinoma (PRAD) using TERT isoform expression.** Cell line PC3 was closest to average isoform percentage from tumour and normal prostate tissues. Cell line TERT promoter status taken from *Ghandi et al., 2019.* Superscript “P” indicated TERT promoter mutation, superscript “WT” indicates wild-type TERT promoter and no superscript indicates no data available. Dashed-line box indicates a zoomed in region of interest with text labels of cell lines. Parameters: Manhattan distance, 2 neighbours and 3 components.


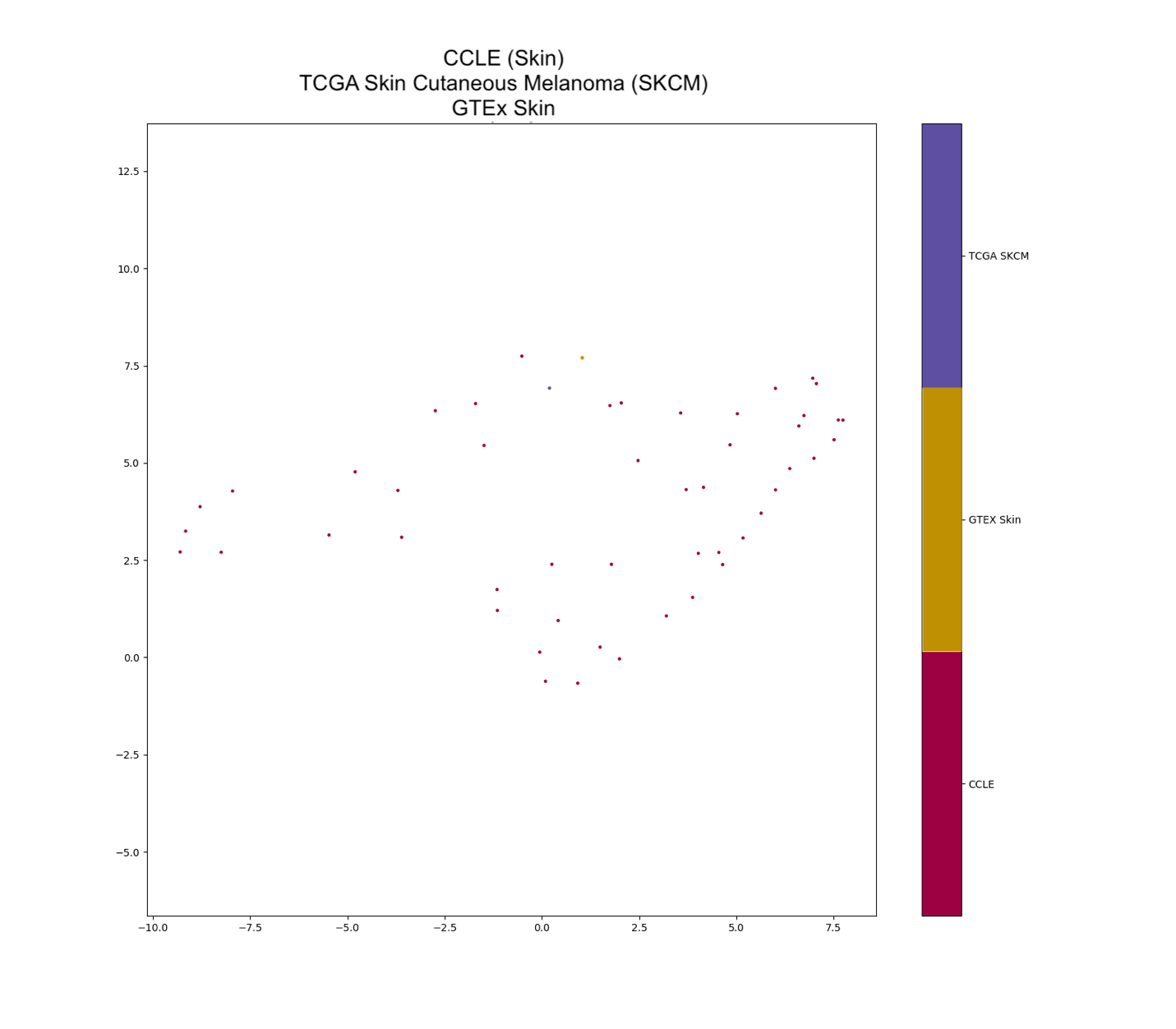

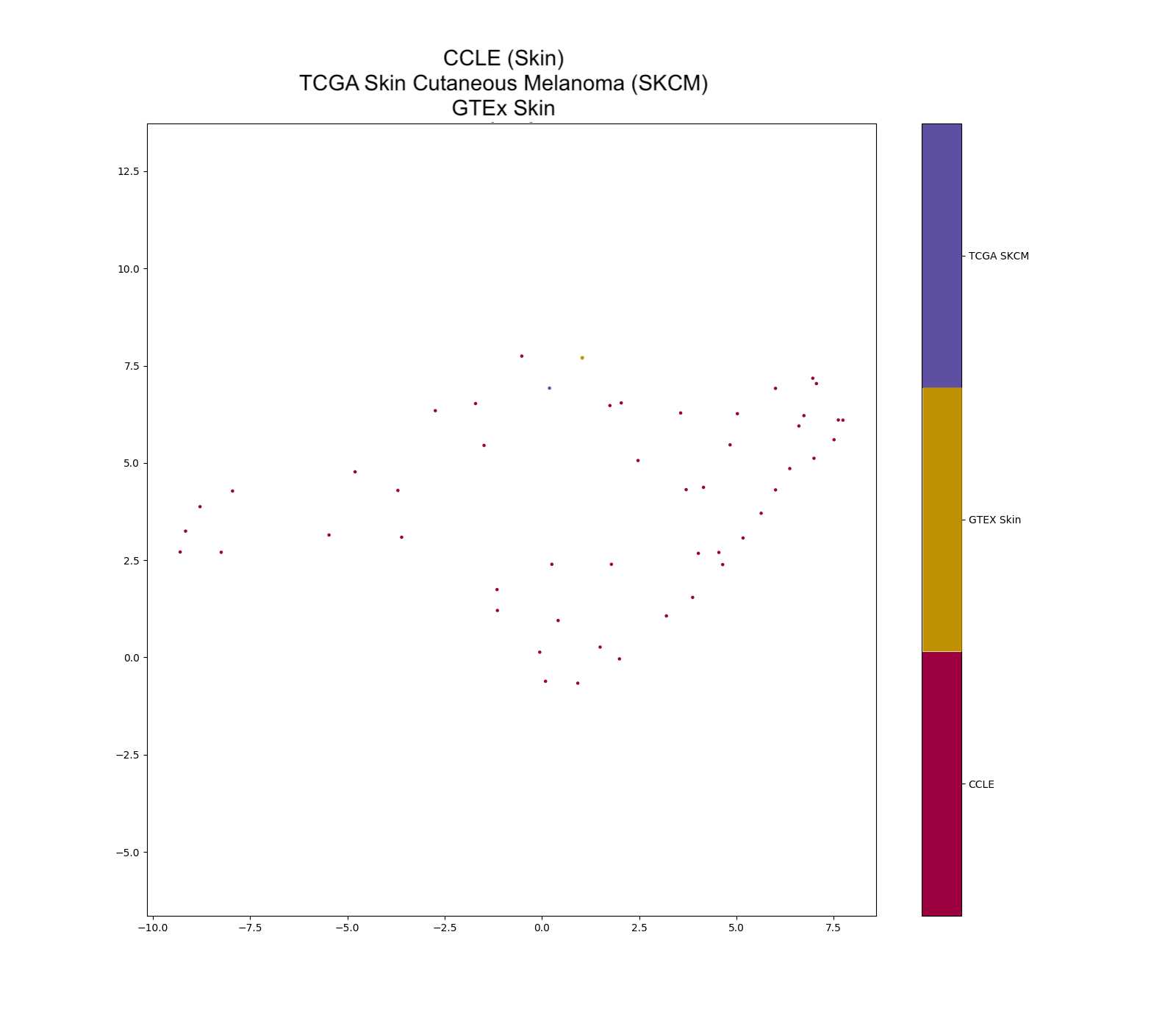


SKMEL1

HT144^P^

SKMEL28^P^

HS695T^P^

**Figure S26. UMAP projection of skin CCLE cell lines, TCGA skin cutaneous melanoma (SKCM) and GTEx skin using TERT isoform expression.** Cell lines SKMEL1, SKMEL28, HT144 and HS695T were closest to average isoform percentage from tumour and normal skin tissues. Cell line TERT promoter status taken from *Ghandi et al., 2019.* Superscript “P” indicated TERT promoter mutation, superscript “WT” indicates wild-type TERT promoter and no superscript indicates no data available. Dashed-line box indicates a zoomed in region of interest with text labels of cell lines. Parameters: Manhattan distance, 8 neighbours and 4 components.


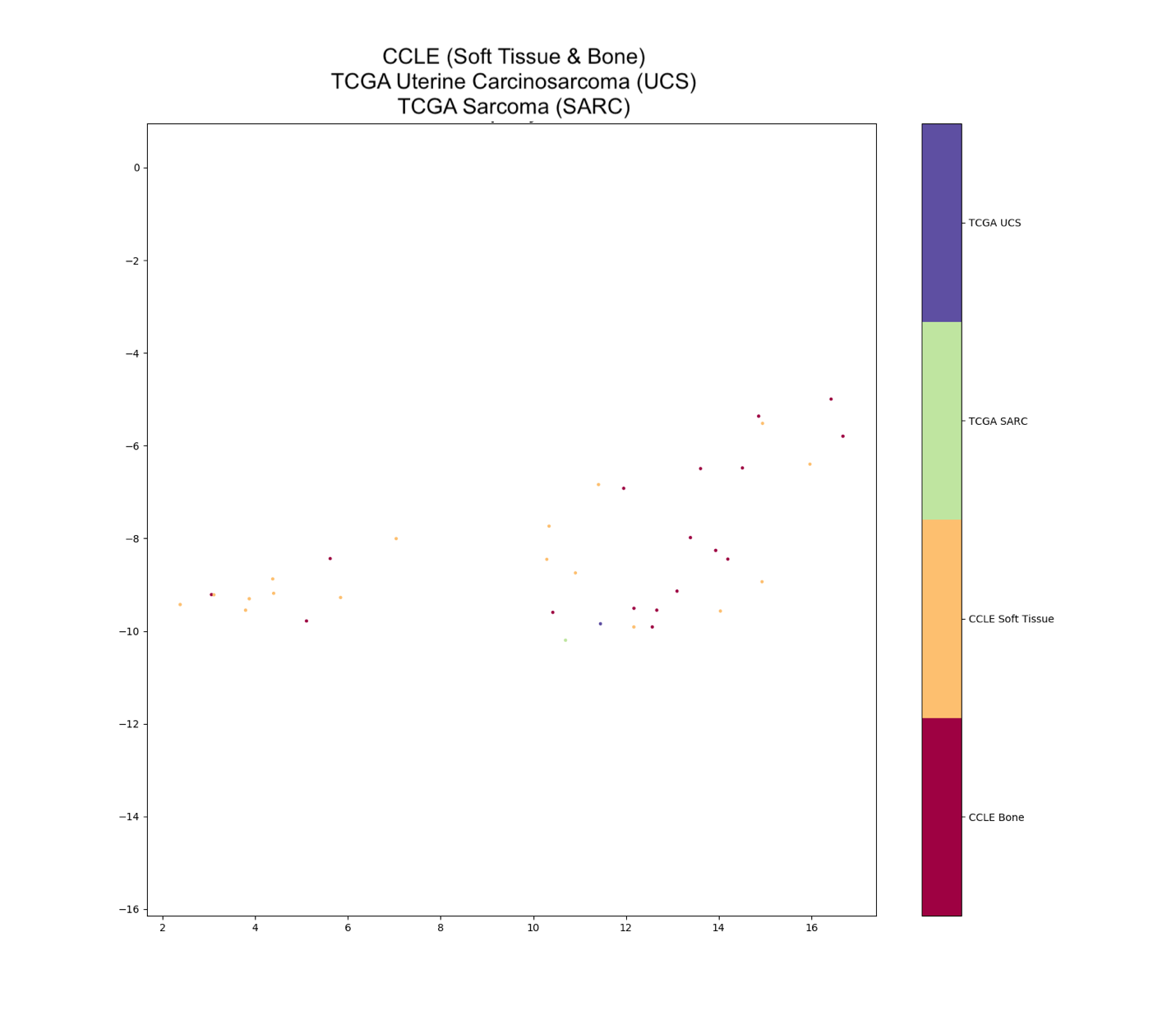

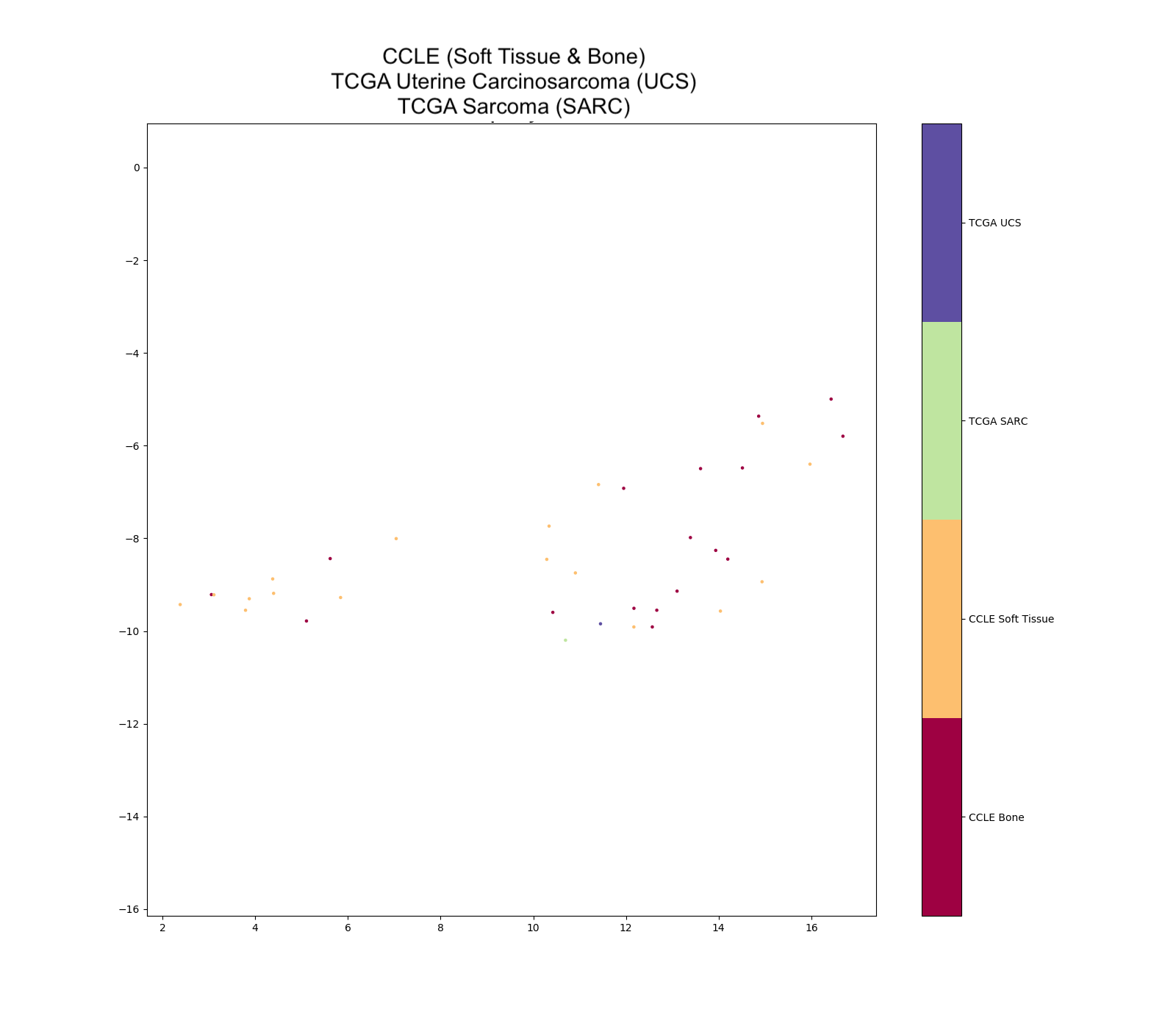


RD

EW8

CADOES1^WT^

**Figure S27. UMAP projection of soft tissue and bone CCLE cell lines, TCGA uterine carcinosarcoma (UCS) and TCGA sarcoma (SARC) using TERT isoform expression.** Bone cell lines EW8 and CADOES1 and soft-tissue cell line RD were closest to average isoform percentage from TCGA UCS and SARC. Cell line TERT promoter status taken from *Ghandi et al., 2019.* Superscript “P” indicated TERT promoter mutation, superscript “WT” indicates wild-type TERT promoter and no superscript indicates no data available. Dashed-line box indicates a zoomed in region of interest with text labels of cell lines. Parameters: Manhattan distance, 8 neighbours and 4 components.


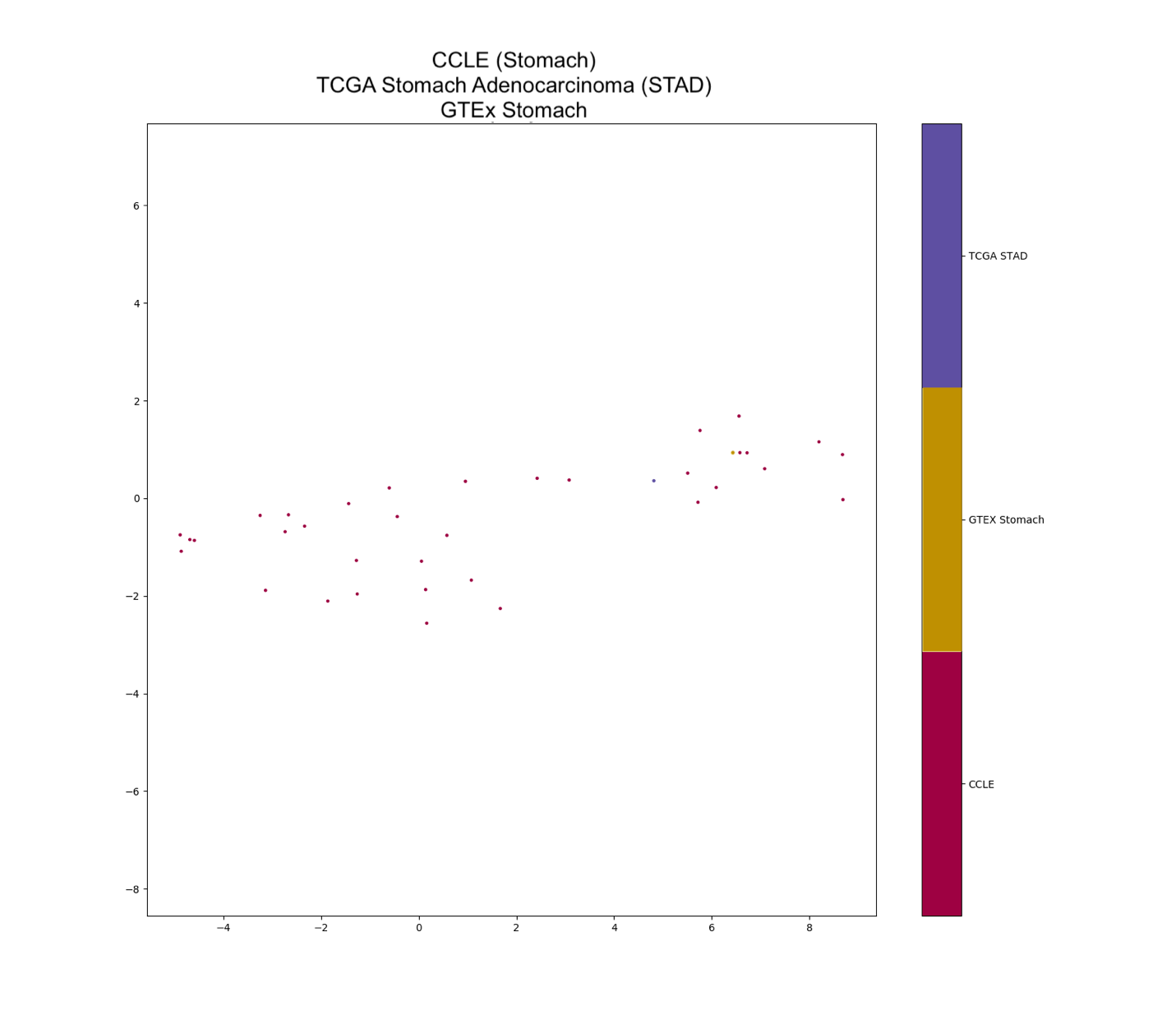

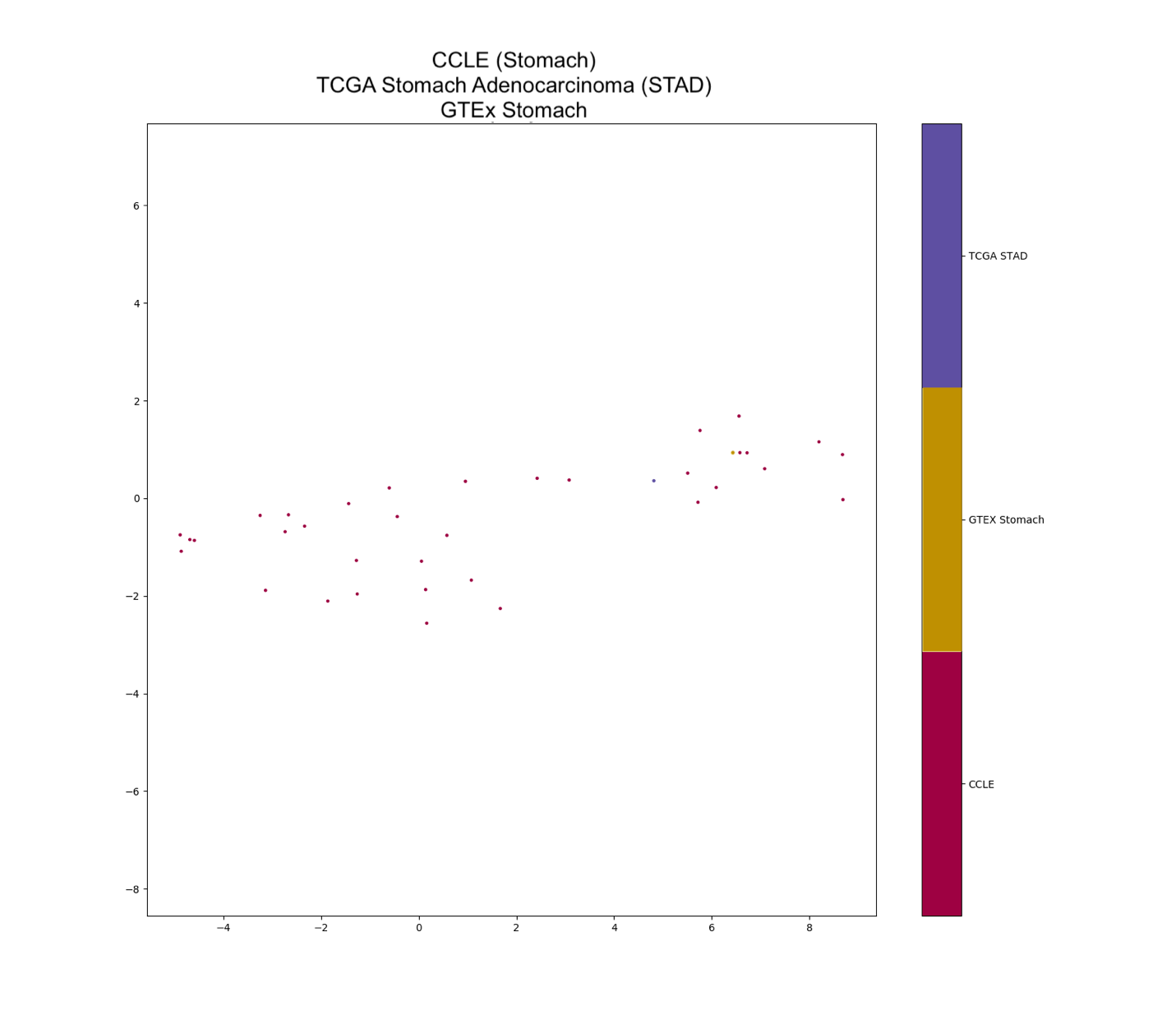


MKN7^WT^

IM95^WT^

OCUM1

SNU216^WT^

FU97^WT^

**Figure S28. UMAP projection of stomach CCLE cell lines, TCGA stomach adenocarcinoma (STAD) and GTEx stomach using TERT isoform expression.** Cell lines MKN7, IM95 and OCUM1 were closest to average isoform percentage from TCGA STAD. Cell lines SNU216 and FU97 were closest to average isoform percentage from GTEx stomach. Cell line TERT promoter status taken from *Ghandi et al., 2019.* Superscript “P” indicated TERT promoter mutation, superscript “WT” indicates wild-type TERT promoter and no superscript indicates no data available. Dashed-line box indicates a zoomed in region of interest with text labels of cell lines. Parameters: Manhattan distance, 8 neighbours and 4 components.


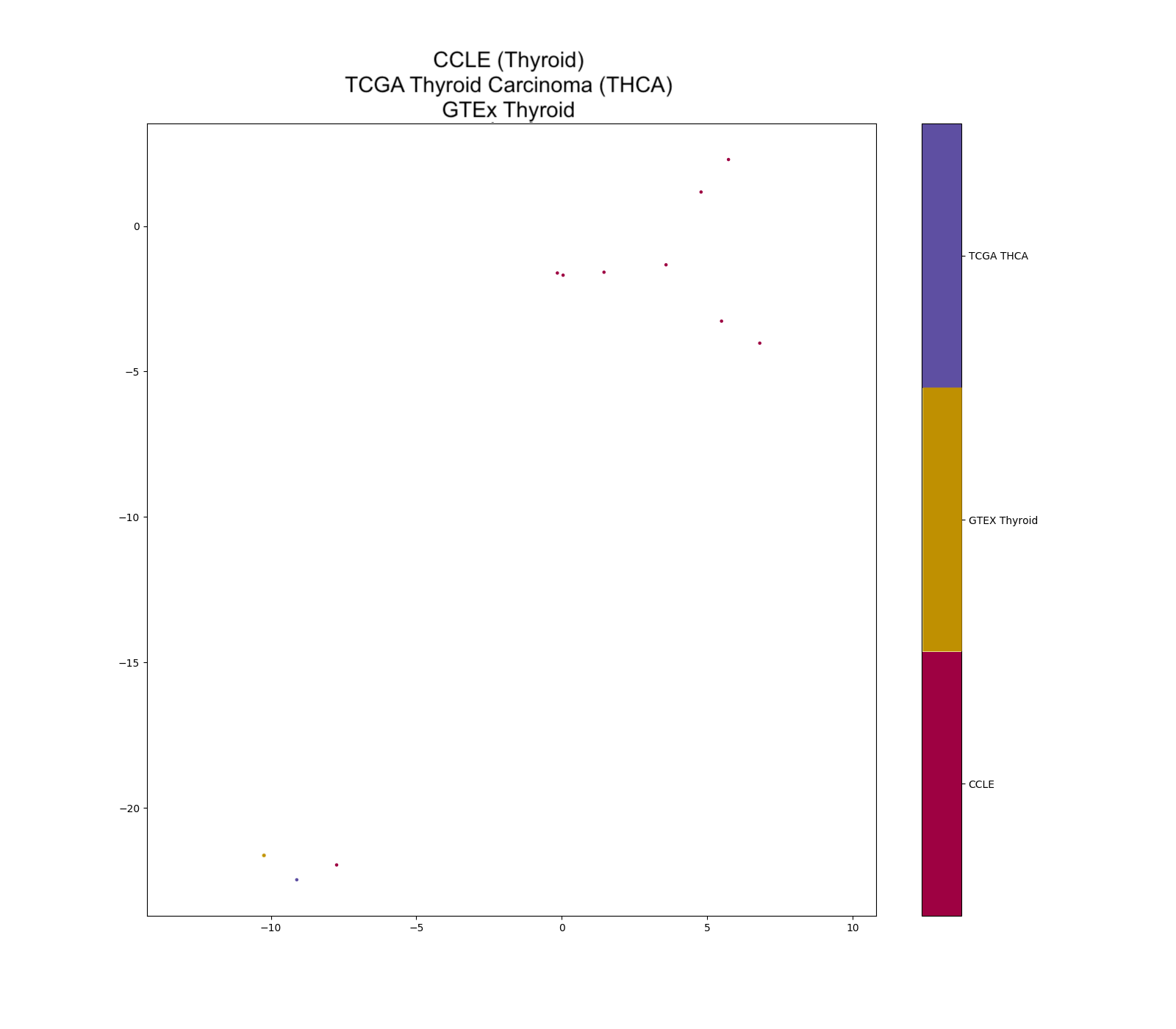

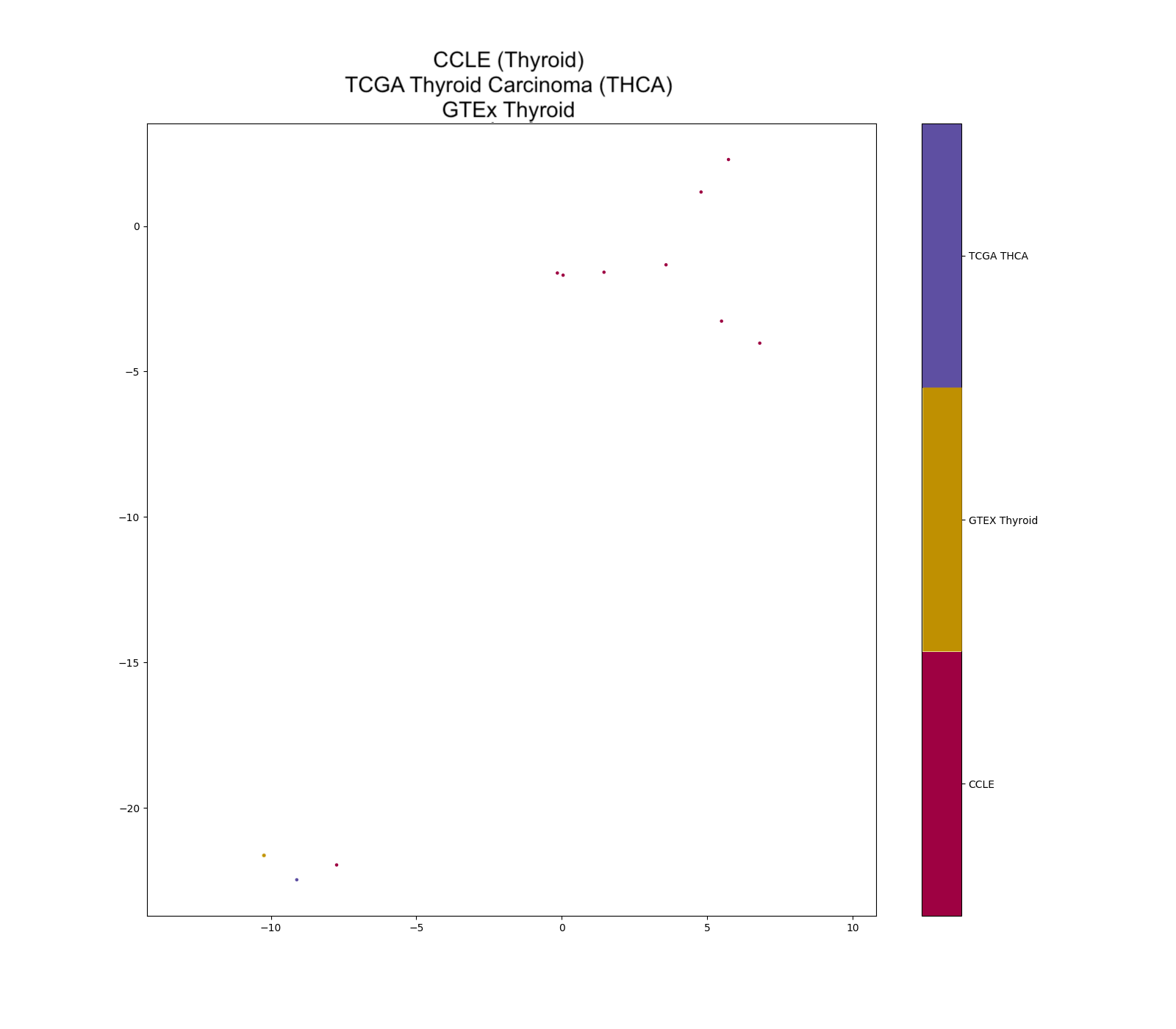


TT2609C02

**Figure S29. UMAP projection of thyroid CCLE cell lines, TCGA thyroid carcinoma (THCA) and GTEx thyroid using TERT isoform expression.** Cell line TT2609C02 was closest to average isoform percentage from tumour and normal thyroid tissues. Cell line TERT promoter status taken from *Ghandi et al., 2019.* Superscript “P” indicated TERT promoter mutation, superscript “WT” indicates wild-type TERT promoter and no superscript indicates no data available. Dashed-line box indicates a zoomed in region of interest with text labels of cell lines. Parameters: Manhattan distance, 2 neighbours and 3 components.


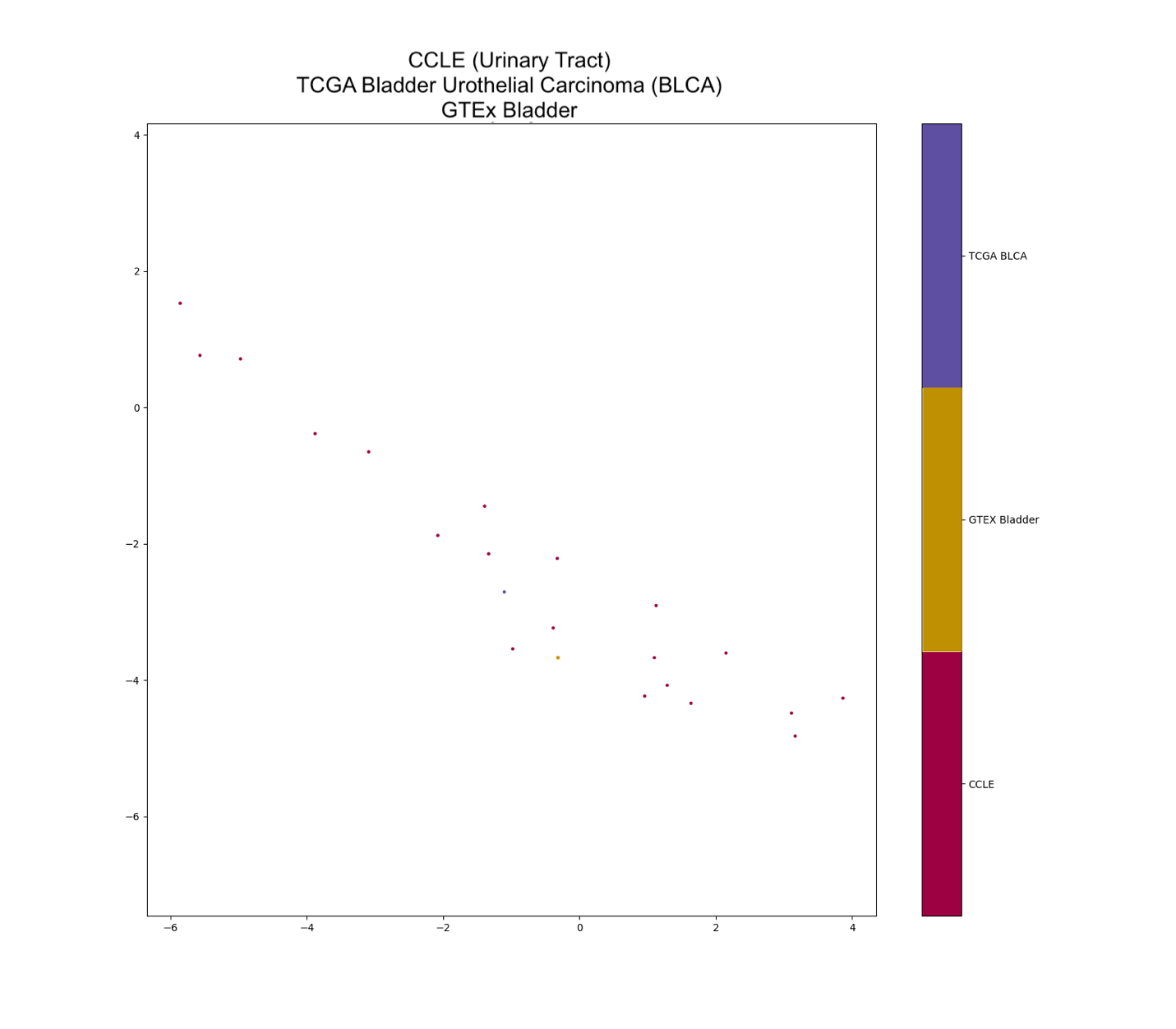

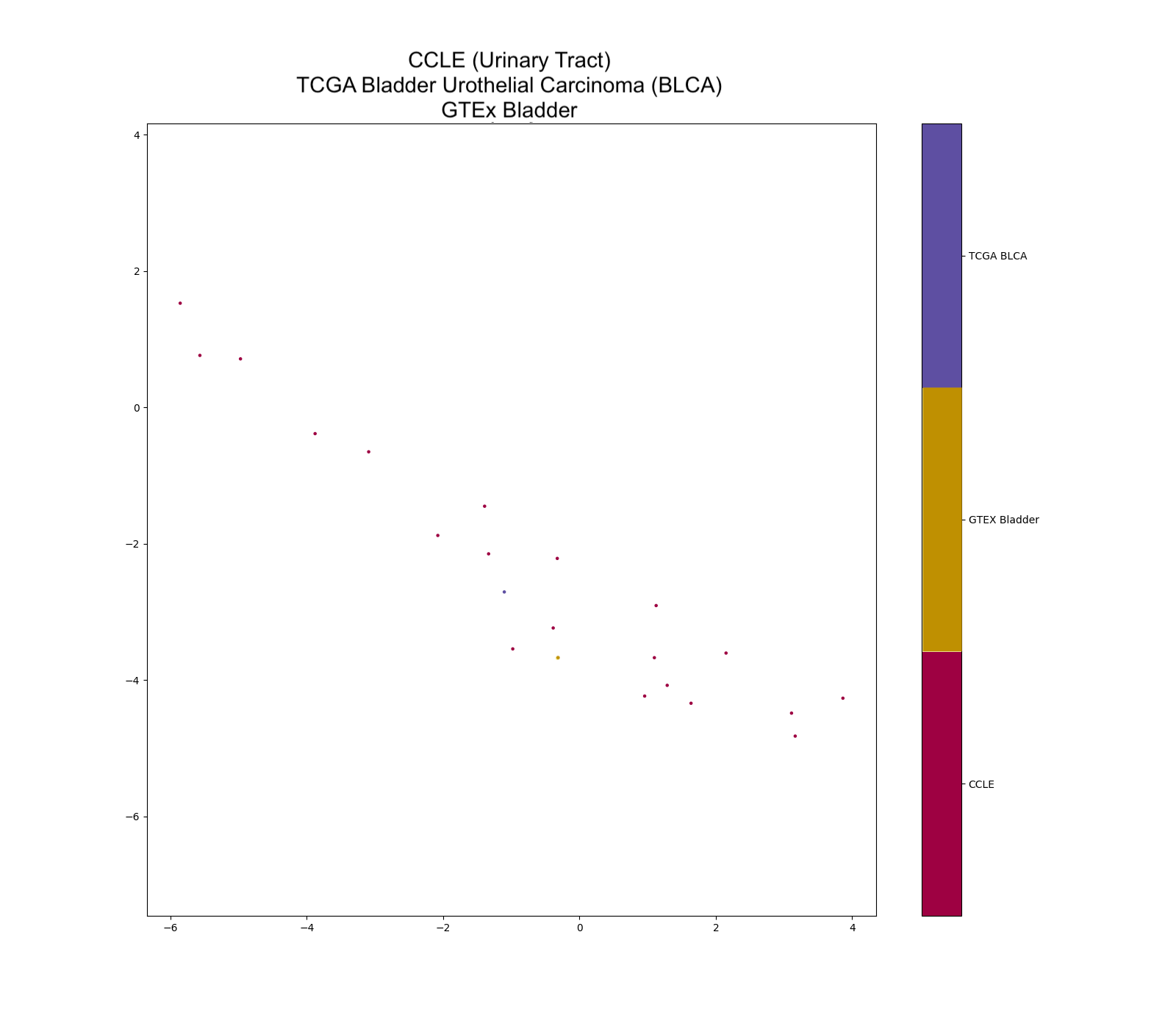


CAL29

RT4

UBLC1

UMUC1

**Figure S30. UMAP projection of urinary tract CCLE cell lines, TCGA bladder urothelial carcinoma (BLCA) and GTEx bladder using TERT isoform expression.** Cell lines UMUC2 and CAL29 were closest to average isoform percentage from TCGA BLCA. Cell lines RT4 and ULBC1 were closest to average isoform percentage from GTEx bladder. Cell line TERT promoter status taken from *Ghandi et al., 2019.* Superscript “P” indicated TERT promoter mutation, superscript “WT” indicates wild-type TERT promoter and no superscript indicates no data available. Dashed-line box indicates a zoomed in region of interest with text labels of cell lines. Parameters: Manhattan distance, 4 neighbours and 4 components.

******

**Figure S31. NOVA1 expression is significantly higher in the FL-TERT expressing group of only LUAD.** NOVA1 expression was significantly different between FL-TERT expressing groups in 3/33 cancer types. Box plots boxes denote the inter-quartile range as well as a bolded line representing the median. Extending from the boxes are minimum and maximum lines calculated from 1.5 times the interquartile range. Points outside this range are considered outliers. Within each box is a red point signifying the mean, as well as lines extending from this point representing a 95% confidence interval. Mean values are presented under each box. Transcript per million (TPM) values were transformed by a log2(TPM + 0.001) equation. A Wilcoxon rank sum test (also known as the Mann-Whitney U-test) was performed with a Bonferroni corrected p-value set at < 0.00152 (0.05/33 comparisons) to determine significance. If significant, the boxplot was given a red border.

******

**Figure S32. PTBP1 expression is significantly higher in the FL-TERT expressing group of many cancer types.** PTBP1 expression was significantly different between FL-TERT expressing groups in 10/33 cancer types. Box plots boxes denote the inter-quartile range as well as a bolded line representing the median. Extending from the boxes are minimum and maximum lines calculated from 1.5 times the interquartile range. Points outside this range are considered outliers. Within each box is a red point signifying the mean, as well as lines extending from this point representing a 95% confidence interval. Mean values are presented under each box. Transcript per million (TPM) values were transformed by a log2(TPM + 0.001) equation. A Wilcoxon rank sum test (also known as the Mann-Whitney U-test) was performed with a Bonferroni corrected p-value set at < 0.00152 (0.05/33 comparisons) to determine significance. If significant, the boxplot was given a red border.

**Figure S33. Data filtering workflow from the total TOIL recomputed dataset to only TERT-positive TCGA and matched GTEx samples.** The total TOIL dataset has 19,131 samples. From here, TARGET (paediatric cancers), normal tissue types without matched tumour tissue types, and TERT-negative samples were removed. In total, there were 2,099 normal (GTEx) samples and 7,887 tumour (TCGA) samples used.

**Table S1. Selected subtypes for each TCGA cancer type identified through independent molecular profiling**

| Cancer Type | Selected | Sample # | Reference Paper |
| --- | --- | --- | --- |
| ACC | DNAmeth | 50 | (Zheng *et al.*, 2016) |
| BLCA | mRNA | 373 | (Robertson *et al.*, 2017*a*) |
| BRCA | COCA | 737 | (Berger *et al.*, 2018) |
| CESC | COCA | 94 | (Berger *et al.*, 2018) |
| CHOL | COCA | 30 | (Farshidfar *et al.*, 2017) |
| COAD | Molecular | 234 | (Liu *et al.*, 2018*c*) |
| ESCA | Molecular | 34 | (Liu *et al.*, 2018*c*) |
| GBM | DNAmeth | 136 | (Ceccarelli *et al.*, 2016) |
| HNSC | mRNA | 233 | (TCGA Network, 2015) |
| KICH | Eosinophilic | 15 | (Davis *et al.*, 2014) |
| KIRC | mRNA | 189 | (TCGA Network, 2013) |
| KIRP | COCA | 30 | (Linehan *et al.*, 2016) |
| LGG | DNAmeth | 312 | (Ceccarelli *et al.*, 2016) |
| LIHC | iCluster | 127 | (TCGA Network, 2017*a*) |
| LUAD | iCluster | 151 | (TCGA Network, 2014*a*) |
| LUSC | mRNA | 42 | (TCGA Network, 2012) |
| OV | COCA | 45 | (Berger *et al.*, 2018) |
| PAAD | Basal | 115 | (TCGA Network, 2017*b*) |
| PCPG | mRNA | 39 | (Fishbein *et al.*, 2017) |
| PRAD | Mutation/Fusion | 264 | (TCGA Network, 2015*b*) |
| READ | Molecular | 76 | (Liu *et al.*, 2018*c*) |
| SARC | iCluster | 80 | (TCGA Network, 2017*c*) |
| SKCM | Mutation | 51 | (TCGA Network, 2015*c*) |
| STAD | Molecular | 337 | (Liu *et al.*, 2018*c*) |
| TGCT | Molecular | 134 | (Shen *et al.*, 2018) |
| THCA | mRNA | 109 | (TCGA Network, 2014*b*) |
| THYM | COCA | 106 | (Radovich *et al.*, 2018) |
| UCEC | COCA | 45 | (Berger *et al.*, 2018) |
| UCS | COCA | 49 | (Berger *et al.*, 2018) |
| UVM | mRNA | 62 | (Robertson *et al.*, 2017*b*) |

COCA = cluster of clusters analysis

1 2 3 4 5 6 7 8 9 10 11 12 13 14 15 16

**1 2 3 4 5 6 7 8 9 10 11 12 13 14 15 16**

TERT_104.2 (Ensembl Build 93)

TERT_820.5 (Ensembl Build 75)

**Figure S34. Ensembl annotations of TERT isoforms used in Toil recomputed dataset (Build 93) and CCLE dataset (Build 75).** The table above indicates the TERT isoforms annotated in both Ensembl Builds – the only difference between the two are the addition of TERT_820.5 in the Ensembl Build 75. While TERT_104.2 and TERT_820.5 have different mRNA sequences, they both only harbour deletions of exons 7 and 8 (beta deletion). This deletion results in a pre-mature stop codon in exon 10, which is why their protein sequences are identical. Full-length (FL)TERT consists of 16 exons. Splicing can involve deletions, which are marked with downward diagonal stripes. ORFs are shown with a light red shade layered over the mRNA structure. Abbreviated names are taken from Ensembl transcript ID. All Ensembl transcripts are either automatically generated from the Ensembl genebuild pipeline or manually annotated by human and vertebrate analysis and annotation (HAVANA); supported by transcriptional evidence either from complementary DNA, expressed sequence tags, or protein sequences.

**Table S2. List of telomerase activity signature score genes and alternative gene IDs used in TOIL recompute.**

| **Signature Score Gene List ID’s from *Barthel et al., 2017*** | **Signature Score Gene List searched in TOIL Recomputed Dataset** |
| --- | --- |
| CECR1 | CECR1 |
| RRAS2 | RRAS2 |
| RARRES2 | RARRES2 |
| CTSC | CTSC |
| LCK | LCK |
| RAD51AP1 | RAD51AP1 |
| KAL1 | ANOS1 |
| FKBP5 | FKBP5 |
| GABRB3 | GABRB3 |
| IFITM1 | IFITM1 |
| ST6GAL1 | ST6GAL1 |
| PIM2 | PIM2 |
| LYPLA1 | LYPLA1 |
| SORL1 | SORL1 |
| ALPL | ALPL |
| NLGN4X | NLGN4X |
| SLC39A10 | SLC39A10 |
| COCH | COCH |
| TXNDC1 | TMX1 |
| GLDC | GLDC |
| C1orf108 | AKIRIN1 |
| DHFR | DHFR |
| PARP1 | PARP1 |
| PASK | PASK |
| POLE2 | POLE2 |
| TRIM22 | TRIM22 |
| PRMT3 | PRMT3 |
| PDCD2 | PDCD2 |
| PAICS | PAICS |
| C1QBP | C1QBP |
| KCNS3 | KCNS3 |
| DCC1 | DSCC1 |
| NFE2L3 | NFE2L3 |
| FRAT2 | FRAT2 |
| ECT2 | ECT2 |
| CCNB1 | CCNB1 |
| C1orf38 | THEMIS2 |
| C14orf106 | MIS18BP1 |
| NUP107 | NUP107 |
| LRRN1 | LRRN1 |
| DSG2 | DSG2 |
| HMMR | HMMR |
| CDC20 | CDC20 |
| CECR1 | CECR1 |
| RRAS2 | RRAS2 |
| RARRES2 | RARRES2 |
| CTSC | CTSC |

******

**Figure S35. Comparison of normal blood leukocyte and normal tissue telomere length.** Telomere length data was taken from *Barthel et al., 2017* to determine which matched normal telomere length estimate to use for normalizing tumour telomere estimates, due to significant relationships with clinical variables such as patient age. Using a Wilcoxon rank sum test we compared telomere length estimates from normal peripheral blood leukocyte (NB) with normal matched adjacent tissue (NT) for each tumour type. We found significantly (p < 0.05) longer telomere length estimates for NT measurements in bladder (BLCA), liver (LIHC), lung (LUSC) and stomach (STAD) cancers.
